# SPL13 controls a root apical meristem phase change by triggering oriented cell divisions

**DOI:** 10.1126/science.ado4298

**Published:** 2024-11-15

**Authors:** Baojun Yang, Yanbiao Sun, Max Minne, Yanhua Ge, Qianru Yue, Vera Goossens, Eliana Mor, Brenda Callebaut, Kevin Bevernaege, Johan M. Winne, Dominique Audenaert, Bert De Rybel

**Affiliations:** 1 Ghent University, Department of Plant Biotechnology and Bioinformatics, Ghent, Belgium; 2 VIB Center for Plant Systems Biology, Ghent, Belgium; 3 State Key Laboratory of Plant Cell and Chromosome Engineering, Institute of Genetics and Developmental Biology, Chinese Academy of Sciences, Beijing, China; 4 University of Chinese Academy of Sciences, Beijing, China; 5 VIB Screening Core, Ghent, Belgium; 6 Ghent University Centre for Bioassay Development and Screening (C-BIOS), Ghent, Belgium; 7 Ghent University, Department of Organic and Macromolecular Chemistry, Ghent, Belgium

## Abstract

Oriented cell divisions are crucial to determine the overall morphology and size of plants, but what controls the onset and duration of this process remains largely unknown. Here, we identified a small molecule which activates root meristem expression of *SQUAMOSA PROMOTER BINDING-LIKE13*, a known player in the shoot juvenile to adult transition. This leads to oriented cell divisions in the root meristem via SHORT ROOT and cell-cycle regulators. We further show that the root meristem has distinct juvenile and adult phases typed by morphological and molecular characteristics and that SPL factors are crucially required for this transition in Arabidopsis and rice. In summary, we provide molecular insights into the age-dependent morphological changes occurring in the root meristem during phase change.

Plants modulate organ size, shape and tissue patterning via oriented cell divisions. These are made possible via differential cell expansion and changes in cell wall anisotropy (1). For brevity, we will refer to the process of modulating cell expansion and cell wall anisotropy resulting in oriented cell divisions as ‘oriented cell divisions’ throughout the text. Several molecular factors that direct these cell proliferation processes in distinct cell populations in the root apical meristem (RAM) have been identified in Arabidopsis over the past few years (2–5). One example operating in the vascular tissues is the heterodimer complex formed by the basic helix-loop-helix transcription factors TARGET OF MONOPTEROS5 (TMO5) and LONESOME HIGHWAY (LHW). This protein complex is required and sufficient to modulate oriented cell divisions by locally activating multiple classes of enzymes all leading to higher levels of active cytokinin in the xylem cells (6–13). This cytokinin is thought to move to the surrounding procambium cells and trigger cell proliferation in the primary root meristem. Downstream of TMO5/LHW, the cytokinin-inducible DOF2.1 transcription factor is also sufficient to affect oriented cell divisions in a subset of procambium cells (14). The closely related mobile *PHLOEM EARLY DOF* (*PEAR*) genes are also induced by cytokinin and the respective proteins diffuse through plasmodesmata forming a short-range concentration gradient peaking in the protophloem sieve elements (15). The PEAR proteins lead to oriented cell divisions in and around the phloem pole sieve elements. Furthermore, in the root apical meristem, the mobile transcription factor SHORT ROOT (SHR) has been elaborately shown to move to the endodermis where it interacts with SCARECROW (SCR) to activate expression of *CYCD6;1*, thereby controlling oriented cell divisions in the ground tissues (16–21). Additionally, TMO5/LHW was shown to induce *SHR* expression in xylem cells. In the neighboring procambium cells, the mobile SHR protein triggers expression of *CYTOKININ OXIDASE3* (*CKX3*), thereby balancing the induction of active cytokinin levels and cell proliferation (9).

Oriented cell divisions are also a prominent feature during vegetative development in the shoot apical meristem (SAM) which can be divided into four phases: a seedling stage, a juvenile stage, and a transitional stage leading to an adult stage. The transition between the juvenile and adult stage is termed vegetative phase change (22) and is controlled by the miR156-dependent SQUAMOSA PROMOTER BINDING PROTEIN-LIKE (SPL) transcription factors (23–25). Amongst the many reported morphological changes occurring during this process, there is a gradual increase in SAM size by oriented cell divisions (26, 27). Recently, SPL9 was shown to directly activate the expression of *CYCD3* family genes, thereby controlling cell cycle duration during the different stages of leaf development (28). More abrupt changes include the appearance of trichomes on the abaxial side and leaf serrations (29). Although the concept of transitions between distinct juvenile and adult phases of development have not been extrapolated to the RAM as such, there are several parallels that can be drawn. For example, there is a very prominent but so far underexplored increase in cell division activity between the first and third week after germination (30). This process is distinct from the general activation of the root meristem during the first week after germination in the seedling stage (31–33), the developmental state generally used to study root biology. Although the morphological changes occurring in the first week of growth have been suggested to represent a phase change in the root apical meristem (34), this process seems unrelated to the dramatic increase in root meristem width which has been observed during the following two weeks of growth of the primary root meristem (30). Although a role for WOX5 has been shown (30) during this later occurring process, the overall surge in cell divisions in the meristem has not been explored in detail and molecular regulators remain unknown. In contrast, a well-studied difference between young and older meristems is the appearance of an additional ground tissue layer called the middle cortex (35–39). This process is controlled by a regulatory network involving gibberellic and abscisic acid (36, 37, 40–43) and a handful of transcription factors including SHR and SCR (17, 36, 37, 40, 43–45), but it remains unclear how the onset of this developmental process is controlled.

Here, we identified a small molecule capable of reorienting cell divisions in the root apical meristem. By analyzing the set of genes induced by this chemical, we identified that the transcription factor *SPL13* and its homologs are necessary and sufficient to control cell division orientation via SHR and cell cycle regulators. These oriented cell divisions are a hallmark of a phase change of the root apical meristem from a juvenile to an adult state which is conserved in both Arabidopsis and rice.

## Results

### A chemical screen to study cell division orientation

**Fig. 1:**
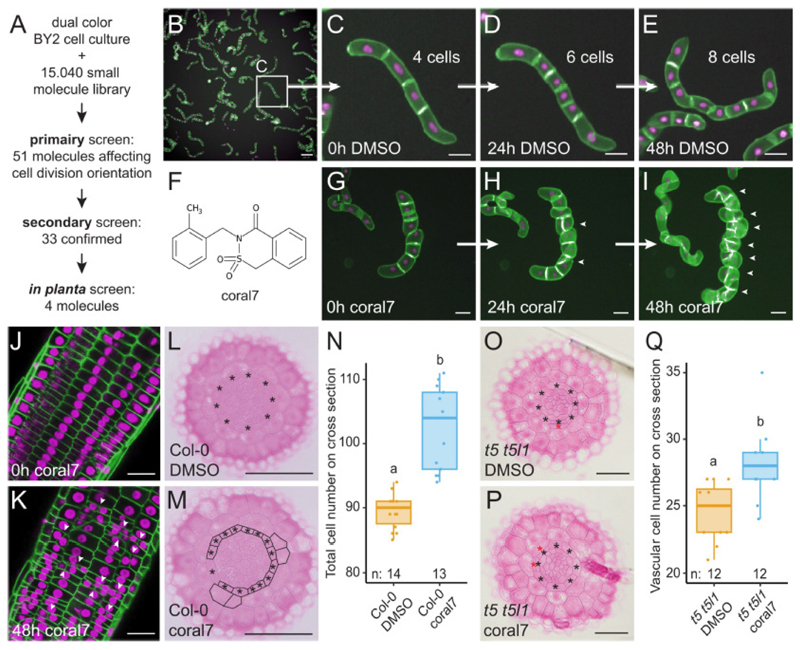
Chemical genetics screen for small molecules affecting cell division orientation. (**A**) Overview of the screening procedure. (**B**) Overview of the dual color BY2 cell culture. Scale bar is 200 μm. (**C**-**E**) A BY2 filament undergoing oriented cell divisions in control conditions (DMSO). Scale bar is 50 μm. (**F**) Chemical structure of coral7. (**G**-**I**) Effect of coral7 treatment on cell division orientation. Scale bar is 50 μm. (**J**-**K**) Confocal images of root meristems of 5 DAG seedlings expressing p*35S*::FH6-GFP (green plasma membrane) and p*35S*::H2B-RFP (magenta nuclei) showing the effect of coral7 in cortex cells. Arrowheads: induced ectopic cell divisions. Scale bar is 25 μm. (**L**-**M**) Histochemical cross sections through the root meristem of 6 DAG seedlings grown on control medium (L) or 50 μM coral7 for 48 hours. Black asterisks: endodermis; red asterisks: additional ground tissue layer. Scale bar is 50 μm. (**N**) Quantification of the total cell numbers in L-M. (**O**-**P**) Histochemical cross sections through the root meristem of 6 DAG seedlings grown on control medium or 50 μM coral7 for 72 hours. Black asterisks: endodermis; red asterisks: additional ground tissue layer. Scale bar is 50 μm. (**Q**) Quantification of the vascular cell numbers in O-P.

To identify chemicals specifically affecting cell division orientation in plants, we engineered a Tobacco Bright Yellow-2 (BY2) cell culture system, which allows tracking cell divisions and their orientation over the course of several days in a miniaturized format. Cell-based systems have been successfully used for chemical genetics approaches in plants for screening, for example molecules affecting protein sorting (46, 47) or the cytoskeleton (48). However, these systems have not been adopted for studying cell division orientation. Thus, to follow the divisions over time, we first generated a dual color reporter in BY2 cell cultures by co-transformation of a nuclear (p*35S*::H2B-mCherry) and a plasma membrane (p*35S*::CPK17-YFP) marker (Fig. S1A). After careful optimization of growth conditions and adapting it to 96-well plate format (see Fig. S1B and materials and methods section for details), this reporter allows cell divisions to be followed over the course of 2 days in an automated screening platform (Fig. 1A-E). In this way, we were able to observe the orientation of the newly formed cell plate in individual BY2 filaments. Based on this system, we conducted screening of a commercial library of 15,040 small molecules at 50 μM final concentration and recorded images at 0, 24 and 48 hours after small molecule addition. We identified 747 molecules that were toxic to the BY2 cells (Fig. S1C-D). After manual analysis of over 45,000 images in a primary screen, we carefully categorized all observed cellular phenotypes for each of the small molecules (Fig. S1E-L and Data S1) and found that 51 molecules affected cell division orientation in BY2 cells. We next performed a secondary screen on these 51 molecules, which we termed coral1 - coral51 for cell division orientation altering chemical, using high-resolution confocal imaging to confirm their effect on cell division orientation. After excluding those molecules affecting cytokinesis, we observed a very specific effect on cell division orientation without affecting division potential in general for 33 small molecules (Data S1).

To determine if any of these molecules were able to affect cell division orientation *in planta*, we transferred *Arabidopsis thaliana* seedlings harboring a dual color reporter line (p*35S*::FH6-GFP and p*35S*::H2B-RFP) (49) to medium supplemented with these molecules at 50 μM concentration and evaluated cell division orientation in the root apical meristem at 24 and 48 hours after transfer. For clarity, we distinguish between three types of oriented divisions in plants: anticlinal, radial and periclinal (Fig. S1M). From our selection, finally four small molecules (coral7, coral8, coral11 and coral13) with unrelated chemical structures were able to trigger periclinal and radial cell divisions both in BY2 cell cultures (Fig. 1F-I and Fig. S2A-D) and in the cortex cell layer of the root apical meristem (Fig. S3A-J) which normally only undergoes anticlinal divisions (14). Given that coral7 treatment resulted in more strict 90 degree switching in cell division orientation (Fig. 1J-K and Fig. S3C-D and K-P) as seen for genetic cues such as e.g. TMO5/LHW (8), DOF2.1 (14) or PEAR1 (15), rather than the more oblique divisions observed for coral8, coral11 and coral13 (Fig. S3E-J), we focused our attention on coral7 for further analyses. Coral7 treatment increased the number of cells in the root meristem (quantified on a radial section halfway between the quiescent center and the elongation zone) (Fig. 1L-N). Coral7 treatment increased auxin signaling (Fig. S4A-G) (50), but we did not observe obvious changes in the expression patterns or levels of well-known reporter lines for microtubules (p*35S*::TUA6-GFP (51)), cell plate formation (p*KNOLLE*::GFP-KNOLLE (52)), actin (p*35S*::lifeact-Venus (53)), polarity protein (p*PIN2*::PIN2-GFP (54) and p*SOK2*::SOK2-GFP (55)), vacuoles (p*RPS5A*::VAMP711-GFP (56)) (Fig. S4H-V), or factors leading to oriented divisions in the vascular cells (Fig. S5A-F). Moreover, coral7 treatment was still able to re-orient cell divisions in vascular cells of the higher order *tmo5* loss-of-function mutant (8, 15) (Fig. 1O-Q, Fig. S5G-H). These results suggest that coral7 affects cell division orientation in the root meristem through a non-canonical pathway. Taken together, our BY2 cell culture-based screening method was successful in identifying small molecules that specifically control cell division orientation in *planta*.

### Coral7 induces *SPL13* expression in the root meristem

Despite the highly specific effect on cell division orientation of coral7, a dose-response experiment showed this molecule was only functional at high micromolar concentrations (50 μM) and after a prolonged treatment of 48 hours (Fig. S6A-E), suggesting that this molecule might not be taken up very efficiently or might need to be metabolized before exerting its activity. We thus performed a structure-activity relationship (SAR) analysis to determine the active sites and potentially generate a more potent analog. We generated 12 structural variants of coral7 named var1-var12 (Fig. S7 and Data S2) and tested the capacity of these modified molecules to induce oriented cell divisions in the root meristem by growing 5 days after germination (DAG) seedlings on 50 μM of each of these variants for 48 hours. The SAR analysis showed that none of the variants was more potent compared to coral7. Most modifications proved deleterious to the bioactivity of the compound, and even a slightly truncated var9 analog lacking only a single methyl group showed no activity. Its isosteric replacement with a bromine atom (var8) did however retain activity. Alkylation of the reactive alpha-position of the sulfonamide was tolerated to some extent, allowing the installation of a clickable group at that position (var12, Fig. S7). This analysis suggested that the chemical structure is overall very sensitive to modifications and that the interaction with its molecular target must be specific. Indeed, biotin labeling at the clickable site in the active analog var12 (Fig. S6F), resulted in a non-functional coral7-biotin conjugate (Fig. S6G-N), as did alkylation with a sterically more demanding group (var12) suggesting that a biochemical assay to find the molecular target of this small molecule is not possible using such modified affinity probes, as its binding is too easily perturbed.

**Fig. 2:**
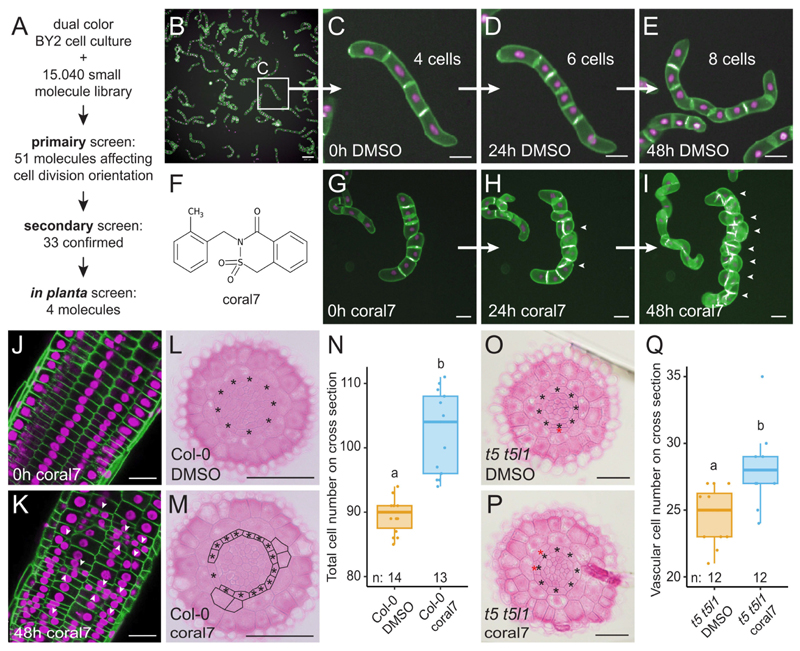
Coral7 induces oriented cell divisions by triggering SPL13 expression. (**A**) RNA-seq strategy. (**B**) Root meristem stained with propidium iodide showing the effect of SPL13 overexpression. Arrowheads: induced reoriented cell divisions. Scale bar is 25 μm. (**C**-**E**) GUS-stained 6 DAG root meristems grown on mock (C-D) medium, or 50 μM coral7 for 48 hours (E). Scale bar in (D) is 50 μm. (**F**-**G**) Sections of 10 DAG root meristems grown on control medium. Scale bar is 100 μm. (**H**) Quantification of the cell numbers in (F-G) (n = 12 for each). (**I**-**J**) 8 DAG root meristems stained with propidium iodide (yellow). Scale bar is 25 μm. (**K**-**L**) 10 DAG root meristems counterstained using propidium iodide. Arrowheads: end of the meristem. Scale bar is 75 μm. (**M**) Quantification of the cell numbers in longitudinal cortex cell files in the root meristem (K-L). (**N**) Quantification of the primary root length in 4 independent p*RPS5A*::SPL13-GFP lines compared to the Col-0 control. (**O**) Relative expression levels of *SPL13* transcripts (n = 3 technical replicates for each genotype). (**P**) Lateral root density in the indicated genotypes (n = 10 for each genotype). Black asterisks in (D) and (F-G): endodermis; red asterisks: additional ground tissue layer.

To understand the mechanism by which coral7 triggers oriented cell divisions in the root apical meristem, we next examined the whole genome transcriptional changes induced by treatment of *Arabidopsis thaliana* root apical meristems with coral7 in a time series experiment for 6, 12, and 24 hours via bulk RNA-sequencing (Fig. 2A and Data S3). We first overlapped the list of differentially expressed genes (DEG) upon coral7 treatment with similar datasets of known transcription factors of oriented cell divisions in the root apical meristem such as TMO5/LHW (7) and DOF2.1 (14). No overlap was found (Data S3), providing further evidence at a molecular level that coral7 affects oriented cell divisions through a non-canonical pathway. After selecting the top 10 DEG upon coral7 treatment (Fig. 2A, Data S3), we constructed ectopic misexpression lines using the strong meristematic *RPS5A* promoter (58) to evaluate the ability of these genes to trigger oriented cell divisions in the root apical meristem as observed upon coral7 treatment. *SQUAMOSA PROMOTER-BINDING PROTEIN LIKE 13A* (*SPL13A/AT5G50570* referred to as *SPL13*) was upregulated around 100-fold at 12 hours in the RNA-seq dataset (Data S3). Misexpression of *SPL13*, but not the other differentially expressed genes tested (Fig. S8A-I, Fig. S9A-I), triggered additional oriented divisions in root meristem cortex cells with (Fig. 2B and Fig. S8J) and without (Fig. S9J) GFP tag, similar to a coral7 treatment (Fig. 1J-K). Fitting with the concept of *SPL13* being one of the genes induced by coral7 treatment, *spl13* mutant backgrounds reduced the effect of coral7 treatment (Fig. S10). Although it is unlikely that SPL13 is a direct target protein of coral7, these results suggest that SPL13 likely acts downstream of coral7 to trigger oriented cell divisions in the root apical meristem.

### *SPL13* misexpression affects cell division orientation

SPL transcription factors are well-studied for their important role in the aerial parts of the plant during the transitions from a juvenile to adult shoot apical meristem and from a vegetative to a flowering meristem (25, 59–62). In the root, SPL9, SPL10 and miR156 have been shown to be involved in root branching and root meristem length determination (34, 63, 64). To analyze a putative role for SPL13 in the cellular changes leading to cell division orientation in the root meristem, we first examined SPL13 levels in the root. No localization was observed in the root meristem of a miR156 sensitive translational reporter line (p*SPL13*::sSPL13-GUS (60) (Fig. S11A) or in a previously generated scRNA-seq expression dataset of the root meristem (65). Given SPL transcripts are repressed by miR156 (59), we next analyzed a miR156 resistant version of the translational SPL13 reporter line (p*SPL13*::rSPL13-GUS (60)) and observed most pronounced localization in the procambium cells (Fig. 2C-D). Upon treatment with coral7, both sSPL13 and rSPL13 protein levels were induced (Fig. S11A-B, Fig. 2E compared to Fig. 2C), which is consistent with our bulk RNA-seq results (Data S3). Taken together, these results confirm that both transcripts and SPL proteins are detected in the root meristem and levels are increased by coral7 treatment.

We next analyzed the root phenotype of the newly generated p*RPS5A*::SPL13-GFP misexpression line in more detail. Consistent with previous reports on the function of SPLs (34, 63, 64), increasing *SPL13* expression levels led to longer root meristems (Fig. 2K-M), an increased root length (Fig. 2N and Fig. S11G-H) and reduction in lateral root density (Fig. 2P). In addition to these phenotypes, the most striking effect of increasing *SPL13* expression levels were ectopic radial and/or periclinal divisions in all cell types of the root apical meristem (Fig. 2F-H, K-L), leading to a strong increase in root meristem width. Notably, an additional ground tissue layer was observed (Fig. 2F-H) as seen using the p*SCR*::H2B-YFP (Fig. 2I-J) and p*CO2*::H2B-YFP (Fig. S11C-D) reporter lines for endodermis and cortex, respectively. Most likely, this additional ground tissue layer corresponds to middle cortex formation (37–39). The combination of increased root meristem size, increased root length and reduction in the lateral root density modified the overall root system architecture into a steeper and deeper branching pattern (Fig. S11E-F). These root-related phenotypes were largely correlated to the relative expression levels of *SPL13* (Fig. 2O and Fig. S11I), showing that *SPL13* expression levels need to be tightly controlled during normal development. Congruent with this conclusion, *mir156/157* loss-of-function lines phenocopied the effects of increasing SPL13 levels (Fig. S12). Moreover, like a coral7 treatment, raising SPL13 levels affected auxin signaling as reported previously (66), but caused no obvious changes in the expression patterns or levels of well-known reporter lines (Fig. S13). Taken together, we found that increasing *SPL13* expression levels leads to changes in the normal cell division orientation patterns in root meristem cells resulting in a strong increase in the root apical meristem width. Given these *SPL13* misexpression phenotypes are still present in a *tmo5 tmo5-like1* double mutant (*t5t5l1*) background (Fig. S14A-I) and triggering TMO5/LHW does not induce an *SPL13* reporter line (Fig. S14J-K), it is likely that SPL13 functions independent from the TMO5/LHW pathway (8).

### SPL13 controls a root apical meristem phase change

**Fig. 3:**
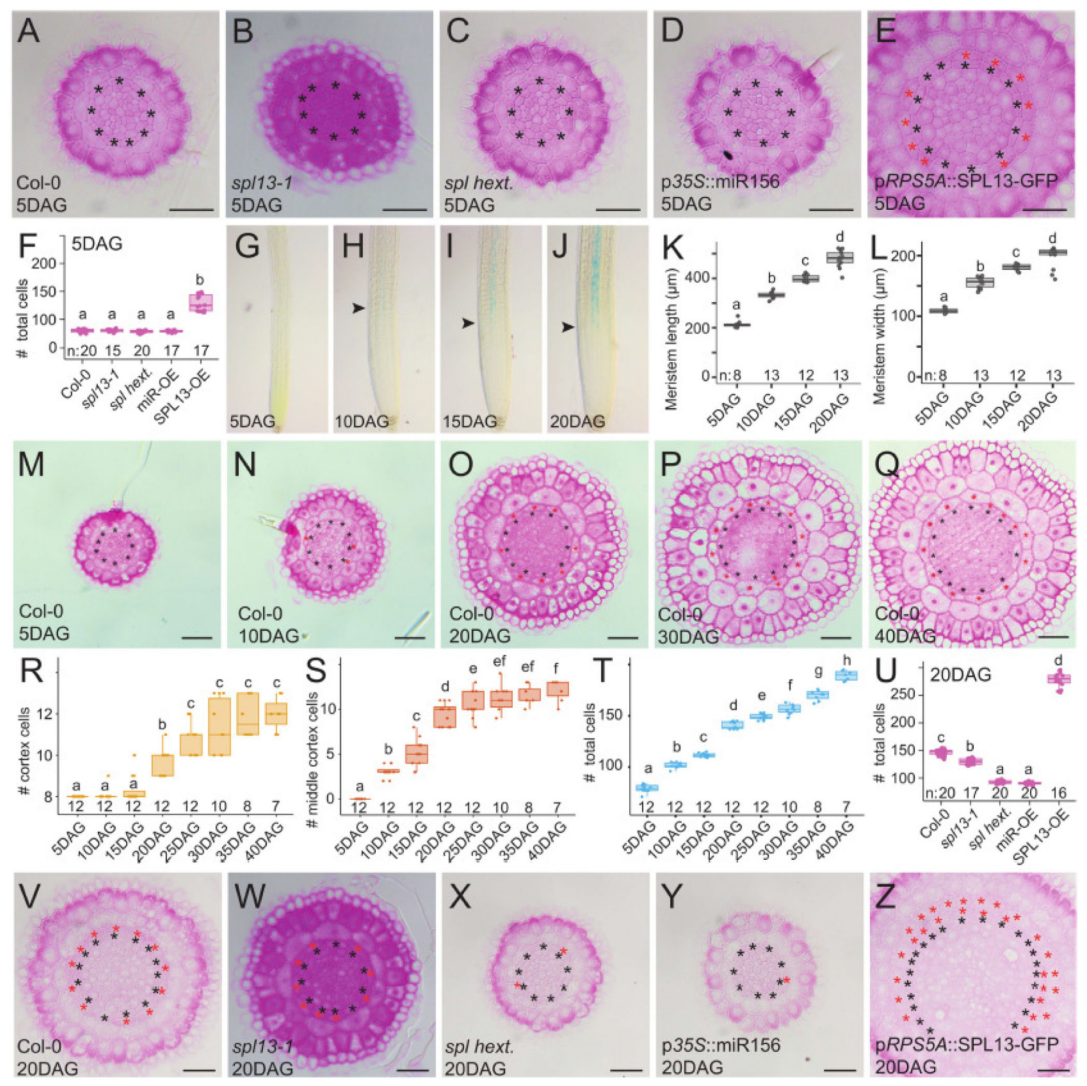
SPL transcription factors control the transition from a juvenile to an adult state of the primary root meristem. (**A**-**E**) Histochemical cross sections through the root meristem of seedlings grown on control medium for 5 days for the indicated genotypes. Scale bar is 25 μm. (**F**) Quantification of the total cell numbers in (A-E). (**G**-**J**) Expression of p*SPL13*::sSPL13-GUS in root meristems grown on mock medium for the indicated time. Arrowheads indicate the first visible expression. (**K**) Quantification of the meristem length in (G-J). (**L**) Quantification of the meristem width in (G-J). (**M**-**Q**) Histochemical cross sections of root meristems of Col-0 grown for the indicated time. Scale bar is 25 μm. (**R**-**T**) Quantification of the number of cortex (R), middle cortex (S) and total (T) cell numbers from cross section in Col-0 control plants grown from 5 to 40 DAG (see also Fig. S22). (**U**) Quantification of the total cell numbers in (V-Z). (**V**-**Z**) Histochemical cross sections through the root meristem of seedlings grown on control medium for 20 days for the indicated genotypes. Black asterisks in (A-E), (M-Q) and (V-Z) indicate the endodermis; red asterisks middle cortex. Scale bar is 25 μm.

To further understand the function of SPL13 during root meristem development, we analyzed roots of seedlings at 5 DAG of the available *spl13-1* single mutant for cell division related phenotypes (60). No significant changes were observed in the overall root growth, nor the number of vascular cells compared to Col-0 wild type plants (Fig. 3A-B, F and Fig. S15). Considering that SPL13 belongs to a family with functional redundancy (60), it is possible other SPL proteins have similar functions. We thus analyzed the localization of root expressed SPL proteins (60) in more detail. SPL2, SPL9, rSPL10, SPL11, SPL13 and SPL15 were all localized in root vascular cells or more broadly in the root meristem under normal growth conditions (Fig. 2C-D, Fig. 3G-J and Fig. S16). To examine functional redundancy due to a similar function in cell division orientation, we generated p*RPS5A*-driven misexpression lines for seven additional SPL transcription factors, fused to GFP. Optical confocal cross sections through the root meristem showed that misexpression of all tested SPL transcription factors are able to trigger cell division orientation changes, resulting in an increased root thickness (Fig. S17). Based on the above results, SPL family members have redundant functions during this developmental process. Hence, we analyzed 5 DAG seedlings of an available *spl2 spl9 spl10 spl11 spl13 spl15* (*spl hextuple* or *spl hext*.) mutant (60) in more detail. Even in this higher order mutant or in the miR156 overexpression line in which all miR156 sensitive *SPL* transcripts are repressed (p*35S*::*MIR156A* (60)), no significant changes were observed at 5 DAG compared to the wild type Col-0 plants when quantifying several phenotypical characteristics in the root meristem (Fig. 3C-D, F and Fig. S15), likely due to miR156 levels being high at this developmental stage (34, 63). We confirmed this by measuring transcript levels of *MIR156* in root meristems over time and evaluating expression of a miR156 sensor (67, 68) (Fig. S18). As this sensor was not affected by a coral7 treatment, coral7 is unlikely to be involved in the miRNA156 silencing function (Fig. S18K-O).

Considering that SPL13 transcripts are targeted by miR156 in early stages of plant growth in the shoot meristem (60), we investigated the tissue specific expression domains of the different miR156 isoforms (*MIR156A, MIR156B, MIR156C* and *MIR156D*) in more detail by generating transcriptional reporter lines (p*MIR156A-D*::nYFP-GUS) using previous described promoter sequences (69) and analyzing their spatiotemporal expression patterns in 5 DAG root meristems. The different miR156 isoforms exhibited tissue-specific expression with *MIR156A* being expressed in phloem cells, *MIR156C* in root epidermis, cortex, QC and vascular initial cells, and *MIR156D* in xylem and columella cells (Fig. S19). QC and columella expression was detected in the root meristem for *MIR156B* (Fig. S20B). We however cannot exclude that sequences in the transcribed region of miR156 isoforms might affect these expression patterns (70). Moreover, miRNA mobility might make the precise expression domains less decisive for the final location of their function. To understand whether the SPL13 protein itself might also be mobile, we drove SPL13 from multiple tissue specific promoters (Fig. S21). In all lines, we observed SPL13 protein in cells neighboring to those where the promoter was active, suggesting SPL13 protein is mobile between cells. Considering the overlapping expression patterns and the cell-to-cell mobility, these results imply a complex spatial interaction between miR156 isoforms and SPLs will eventually define the expression levels of *SPL13* at a specific location in the root meristem.

To investigate the temporal dynamics of this complex regulation, we next examined SPL13 levels and expression of *MIR156* in root meristems over time. Similar to previous reports (63), expression levels of *MIR156* isoforms decreased over time which was shown to influence timing of meristem growth and lateral root emergence (34) (Fig. S20). During this process, levels of SPL13 (Fig. 3G-J) and other miR156 sensitive SPL reporters (Fig. S16), increased over time and became strongly expressed at 20 DAG. This increase in SPL13 and other SPL levels was accompanied by a gradual increase in the number of vascular, endodermis and epidermis cells in a wild-type background (Fig. 3M-Q and Fig. S22). Although cortex cell divisions and differentiation of the middle cortex are suppressed in the first week after germination, the number of cortex cells and the newly emerged middle cortex cells showed a more discrete increase starting around 15-20 DAG and 10 DAG, respectively, and plateauing at later time points (Fig. 3R-S and Fig. S22). These growth patterns observed in a wild type Col-0 root meristem both in *in vitro* and soil conditions (Fig. S22) resemble the transition from the juvenile to the adult phase of shoot development which is also characterised by both discrete and continuous patterns of development (29, 71). All the additional divisions contributed in a general increase in meristem size in both length and width (Fig. 3K-L). The increase in meristem size over time has been reported before (30, 34, 37, 72), but the exact molecular mechanisms triggering this event remained unexplored. Collectively, these results suggest that root apical meristems undergo a growth transition over time from a juvenile state towards an adult state which is characterized by an increase in the number of cells, middle cortex formation and a general increase in both width and length of the meristem.

**Fig. 4:**
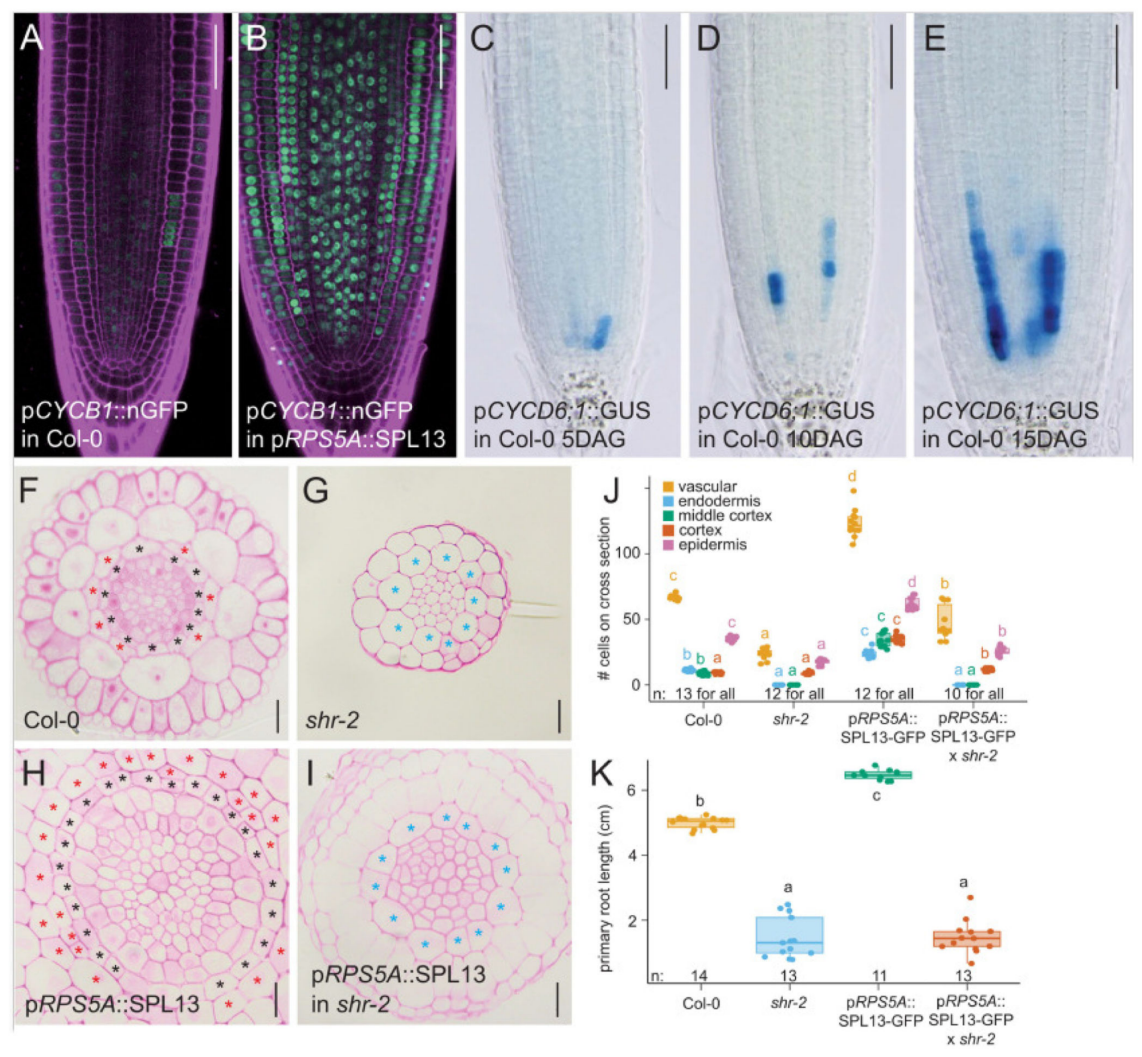
Pathways acting downstream of SPL13. (**A**-**B**) Confocal images of 6 DAG root meristems expressing p*CYCB1;1*::nGFP (green nuclei) and counterstained with propidium iodide (magenta). (**C**-**E**) Light microscopy images of root meristems expressing p*CYCD6;1*::GUS-GFP, grown for the indicated time. Scale bars are 50 μm. (**F-I**) Histochemical cross sections through the root meristem of seedlings, grown for 20 days for the indicated genotypes. Black asterisks indicate the endodermis; red asterisks indicate the middle cortex cells. Note that since *shr-2* mutant has a single ground tissue layer with cortical cell identity we mark it here with a blue asterisk. Scale bar is 20 μm. (**J**) quantification of the number of cells in (F-I) for indicated genotypes. (**K**) quantification of primary root length for indicated genotypes.

Based on its expression during meristem maturation, SPL13 likely plays an important role in the later stages of root meristem development, we next analyzed mature root meristems of the *spl13-1* single mutant, the *spl hextuple* mutants and the miR156 overexpression line at 20 DAG in comparison to the wild type Col-0 control and the *SPL13* misexpression line. In contrast to the wild type situation, the *spl hextuple* mutant and the miR156 overexpression line did not show any of the above mentioned features characterizing the juvenile to adult transition (Fig. 3U-Y). Indeed, the number of cells in the *spl hextuple* mutant and the miR156 overexpression line did not show the strong increase at 20 DAG compared to 5 DAG as seen for the Col-0 control lines (Fig. 3F, U and **Fig. 15B**). Moreover, primary root length was reduced in these lines (Fig. S15A). This indicates that SPL factors are required for juvenile to adult transition in the root meristem. Increasing the relative amount of SPL proteins in the *SPL13* misexpression line further increased the characteristics associated with the juvenile to adult transition, resulting in even more cells and an overall increase in meristem size at any of the analyzed timepoints (Fig. 3E-F, U, Z and Fig. S15). Fitting with these results, and similar to previous reports (28), increased SPL13 levels induced expression of cell cycle regulators (Fig. 4A-E and Fig. S23A-D). Collectively, this indicates that SPL transcription factors are not only required for, but also sufficient to convey a juvenile to adult transition in the root apical meristem of *Arabidopsis thaliana*. The onset of this transition is most notably marked by the formation of the middle cortex (37–39). Although the pathways controlling middle cortex formation have been well characterized (17, 36, 37, 40, 44, 45, 73, 74), the molecular networks controlling the onset of these divisions remained unclear. Our results suggest that the SPL13 is required for middle cortex formation via the canonical SHR pathway (Fig. 4F-K). Similar to previous observations (37), the first ground tissue periclinal divisions always originate at those endodermal cells at the protoxylem poles and next extended throughout the endodermis until an entire additional layer was formed (Fig. S24A-D). From 25 DAG onwards, even a third cortex layer started to form (Fig. S24E-F).

### SPL-dependent root phase change is conserved in rice

**Fig. 5:**
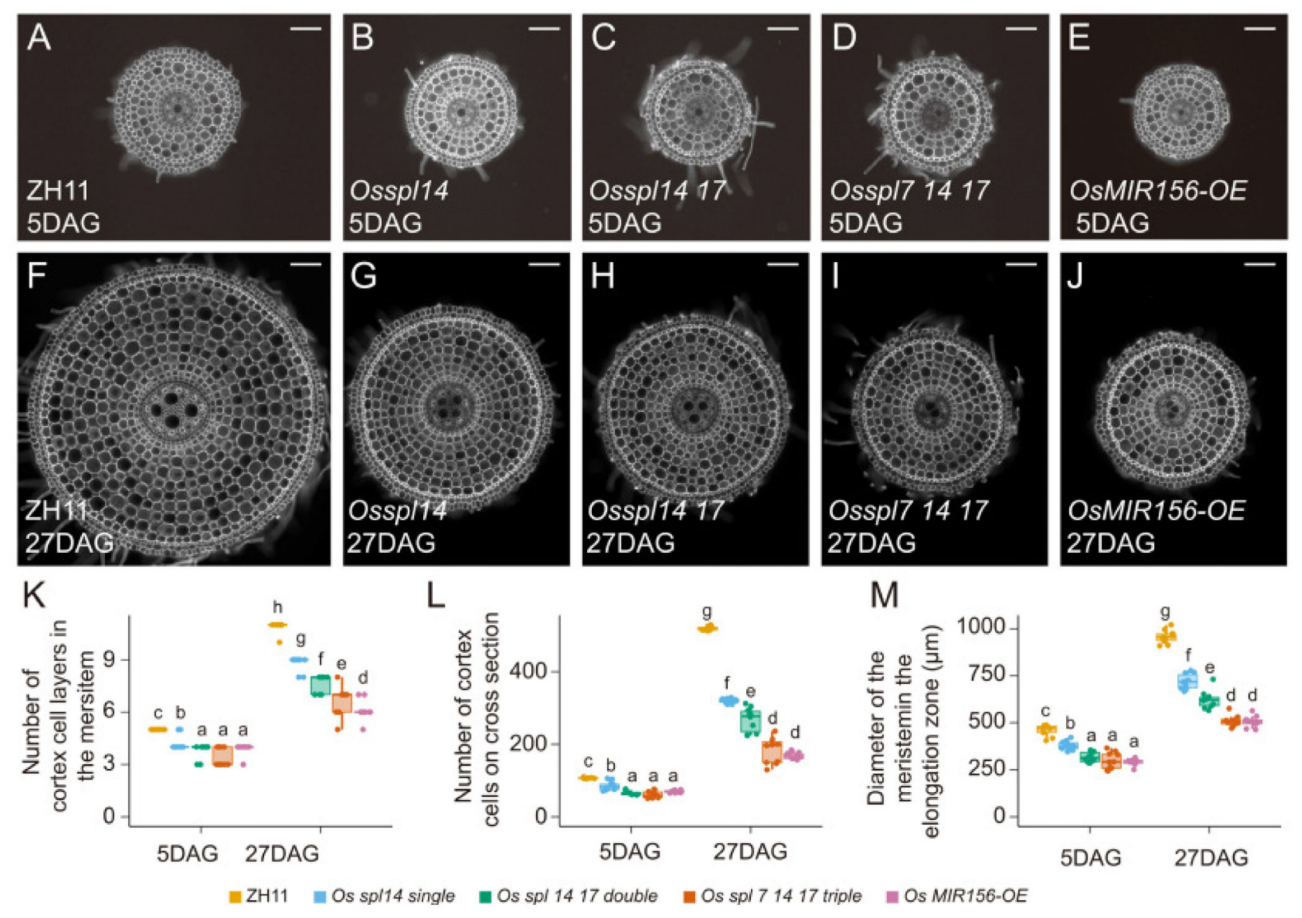
SPL function in the root apical meristem is conserved in rice. (**A-J**) Cross sections of rice root meristems of the control (ZH11), *Osspl14 single, Osspl14 17 double, Osspl7 14 17 triple* mutants and the OsmiR156 overexpression line grown for the indicated time and counterstained with calcofluor white (white). Scale bar is 100μm. (**K**) Quantification of the number of cortical cell files on a cross section in (A-J). (**L**) Quantification of the total number of cells in the cortex cell files in (A-J). (**M**) Quantification of the diameter of the meristem in (A-J). n = 10 for each genotype in (K-M).

To examine whether vascular plants might share a similar mechanism in root juvenile-to-adult transitions, we examined the changes in the meristematic zone and its relationship with SPLs in the monocot crop rice (*Oryza sativa*). We first observed that, similar to our observations in *Arabidopsis thaliana* (Fig. 3M-Q), wild type rice (ZH11) root meristems undergo a transition characterized by an enlarged root meristem and an increase in the number of cells at 27 DAG in comparison to 5 DAG (Fig. 5A, F, K-M). Additionally, overexpression of SPL transcription factors in multiple plant species have been shown to lead to a larger tissue size (66, 75, 76). The rice *SPL* gene family contains 19 members (77), of which *OsSPL14* belongs to the class II of miR156 sensitive SPL proteins (78, 79). Unlike the taproot system in Arabidopsis, rice has as fibrous root system characterized by many adventitious roots. We thus analyzed the newly emerged adventitious root meristem characteristics associated with the juvenile to adult transition in the *Osspl14* single (80), the *Osspl14 17* double, the *Osspl7 14 17* triple mutants and the OsmiR156 overexpression line (66) at 5 DAG and 27 DAG and found that already in the *Osspl14* single mutant, but more pronounced in the higher order mutants and the OsmiR156 overexpression line, these meristem phase transition characteristics are significantly reduced (Fig. 5B-E, G-J). These results show that also in the rice root apical meristematic, cell proliferation during aging is controlled through miR156-SPL module.

## Discussion

The miR156-SPL pathway has been implicated in a wide range of developmental processes in plants (22, 81–83). In most cases, these are linked to age-related processes during vegetative phase transitions in the areal part of the plant, which makes them important targets for breeding and biotechnological applications (84–86). In recent years, it has become clear that the functions of the miR156-SPL pathway are not limited to the shoot region. Indeed, lateral root development, root length and overall root system architecture seem to be dependent on miR156 and *SPL* expression levels as well (34, 63, 64). Here, we show that plant root apical meristems undergo a marked transition from a juvenile state (first week of growth) to a mature state (reached around 3 weeks after growth) which can be typed by specific characteristics, including an increase in the number of cells and middle cortex formation. We thus provide the first molecular insights into this poorly studied conserved developmental process in the root apical meristem (30) by showing that SPL transcription factors are crucially required for and sufficient to control the onset of the transition from a juvenile to an adult state of the root meristem by triggering oriented cell divisions. This is highly similar to the vegetative transition from a juvenile to an adult shoot apical meristem (24, 60, 87). In both cases, there is a clear transition from one phase of development to another, each typed by specific morphological and molecular characteristics related to expression levels of *MIR156* and *SPL* transcription factors. As such, perhaps this gradual transition from a juvenile to a mature vegetative state after the first week of growth comprises a true phase change in the root apical meristem. Despite the obvious similarities between the phase change transitions in root and shoot apical meristems, increased SPL activity has been reported to decrease shoot apical meristem size (88–90), while it leads to a larger root apical meristem. This suggests some level of divergence in the SPL regulatory networks between the shoot and root apical meristems. It would be intriguing to investigate whether the onset of transitions in the shoot and root apical meristem are connected via a shoot-to-root signal (34).

Our results also show that precise control of the expression levels of *SPL* transcription factors, previously characterized as having an important role in vegetative phase change in the shoot apical meristem, appears also crucial to determine the root apical meristem size and the juvenile to adult characteristics in the root. The exact amount of SPL13 is defined by an age-dependent temporal control mechanism in the root meristem driven by miR156 isoforms and cell-to-cell mobility. The *SPL13* misexpression phenotypes characterized by an increased root meristem size and activity can in this context be interpreted as a premature or faster transition from the juvenile to the adult state. In the same line of thought, the reduced *SPL13* transcripts in loss-of-function mutants or the miR156 overexpression line can be considered as a delayed or absent transition, respectively. A final important consideration is that SPL13 localization is not visible in the juvenile root meristems, although this is the most common stage for root biologists to perform experiments on, as the meristem is already considered steady-state at this moment in time (91–94). As such, perhaps other regulators currently hallmarked as controlling shoot apical meristem or vegetative phase change have additional roles during root apical meristem development, but they are simply not expressed during the juvenile state. Indeed, our results imply that understanding root meristem development will require a more holistic approach taking into account these dynamic changes in gene expression associated with the juvenile to adult state transition and might require re-evaluating previous results at later stages of development. Moreover, given the evolutionary conservation of SPL function, precise spatiotemporal modulation of *SPL* expression levels might be an interesting avenue to modulate root growth in general and root system architecture in crop species specifically.

## Materials and Methods

### Plant materials and Growth conditions

Suspension cultures of *Nicotiana tabacum* Bright Yellow-2 cells were grown in liquid medium containing 4.6g/L M&S salts, 30 g/L sucrose, 0.2 g/L KH_2_PO_4_, 1 mg/L Thiamine, 0.2 mg/L 2, 4-D and kept in the dark at 24°C with constant shaking. *Arabidopsis thaliana* ecotype Columbia-0 (Col-0) was used in all transformation and phenotype analysis. Plant lines and mutants used in this study are *spl13-1* (60), *spl2/9/10/11/13/15* (60), *shr-2* (18), *tmo5 tmo5-like1* (8), *mir156ac/157ac* (95), *mir156acd/157ac* (95), p35S::*MIR156A* (60), p*SPL*::sSPL-GUS and p*SPL*::rSPL-GUS *(60)*, p35S::FH6-GFP *and* p35S::H2B-RFP (49), p*TMO5*::TMO5:YFP (8), p*TMO5*::n3GFP (8), p*LOG4*::n3GFP (7), p*PEAR1*::erVENUS (15), p*SCR*::H2B-YFP (96), p*CO2*:H2B-YFP (96), dGR (14), R2D2 (50), p*KNOLLE*::GFP-KNOLLE (52), p*35S*::lifeact-Venus (53), p*PIN2*::PIN2-GFP (54), p*SOK2*::SOK2-GFP (55), p*RPS5A*::VAMP711-GFP (56). All seeds were germinated on 1/2 MS (Murashige and Skoog, Duchefa) medium containing 0.8% agar without sucrose after 3 days stratification at 4 °C. Seedlings and plants were grown vertically at 22 °C in continuous light conditions. *Oryza sativa* japonica rice variety Zhonghua 11 (ZH11), *Osspl14, Osspl7 14 and Osspl7 14 17 CRISPR* mutants (80) and OsmiR156f overexpression line (66) plants were cultivated in a growth chamber with a 12-h-day (30°C)/12-h-night (22°C) photoperiod, with a 50% humidity. The Arabidopsis Genome Initiative identifiers for the genes used in this study are as follows: *SPL2*: *AT5G43270*; *SPL3*: *AT2G33810*; *SPL6*: *AT1G69170*; *SPL9*: *AT2G42200*; *SPL10*: *AT1G27370*; *SPL11*: *AT1G27360; SPL13A: AT5G50570* and *SPL15*: *AT3G57920*.

### Microscopy-based chemical screening

BY2 cells expressing a nuclear (p*35S*::H2B-mCherry) and plasma membrane-specific (p*35S*::CPK17-YFP) reporter fluorescent proteins were prepared for imaging with Operetta CLS™ (Perkin Elmer (now Revvity)). Liquid BY2 culture containing cells sufficient for one 96-well plate was obtained from a 300mL culture, which was maintained for 2-3 days to ensure the presence of young cells. The culture was filtered through a 100μm mesh into individual 50mL tubes, with 50mL of culture allocated to each tube. The tubes were then centrifuged at 1000g for 2 minutes. After centrifugation, the supernatant was discarded, and the cells were carefully recovered from the pellet. The optical density at 600nm (OD600) of the cell pellet was measured and adjusted to a value of 0.06. Subsequently, 100μL of the appropriately adjusted cell suspension was dispensed to each well of a 96-well plate CellCarrier Ultra plates (Perkin Elmer (now Revvity)). To facilitate cell settling at the bottom of the wells, the plate was centrifuged at 1000g for 2 minutes. Nine batches/runs of 20 to 28 assay plates, were prepared and treated with compounds at 50μM final concentration (the Agro-like Library; Enamine, https://enamine.net/). Column 1 of each plate was treated with DMSO as a negative control. Cells were imaged in 3 different channels, brightfield, mCherry (ex: 530-560nm; em: 570-650) and YFP (ex: 460-490nm; em: 500-550nm) with an Olympus 5x, Air, NA0.16 objective, 1 field-of-view per well and 5 Z-stacks with 10μm distance. Cells were imaged 1-4h, 24-28h and 48-52h after compound treatment. Quantitative image analysis included the cell count (number of nuclei, segmented on the mCherry channel). The evaluation of morphological and cell division parameters was performed visually. The coral compounds used in this study are as follows: coral7 (Cat# Z968461102, Enamine), coral8 (Cat#Z45545195, Enamine), coral11 (Cat#Z1150402732, Enamine), coral13 (Cat#Z1139216234, Enamine).

### BY2 marker line cloning and transformation

The dual color BY2 cell line was generated by two step transformation including a PM-YFP marker (Kan) and a nuclear H2B maker (Hyr). The PM-YFP construct was generated by fusing a plasma membrane sequence CPK17 (97) into vector pBIN-PMYA(Kan) and then transformed to BY2 cells. The selected positive PM-YFP BY2 cells was further used for second H2B-mCherry nuclear transformation. To generate the H2B-mcherry expression vector, coding sequences of the H2B (AT5G22880) were cloned into the pDONR221 entry vector using the BP clonase system. The CaMV35S constitutive promoter (pDONRP4P1R-35S) fused to the H2B (pDONR221) and mcherry (pDONRP2RP3) were generated via an LR clonase reaction with the pH7m34GW vector. BY2 cell transformation was performed as described by (98, 99).

### Compound synthesis and biotin labelled coral7

Synthetic protocols and NMR spectra for coral7, the coral7 variants and the biotin-labelled version of coral7 are described in detail in Data S2.

### Coral7 treatment and bulk RNA-seq analysis

Col-0 seeds were bleach sterilized and stratified for 24h at 4°C. Seeds were sown on ½ MS medium containing 0.8% agar without sucrose and grown vertically in a growth room at 22°C in continuous light conditions. 5 DAG Col-0 plants were transferred to ½ MS plates containing 50μM coral7 and solvent control plates and were sampled at the following time points: 0h, 6h, 12h and 24h. 300 individual root tips were sampled per sample and three biological repeats per time point were used. Root tips were harvested directly into liquid nitrogen, RNA was extracted using the RNeasy kit (QIAGEN). The *Arabidopsis thaliana* Col-0 TAIR10 Genome data was used for mapping and annotation. The analysis was performed with R software package edgeR (R version 3.5.1). Genes with expression values higher than 0.35 cpm (5 read counts) on at least 3 samples were retained for the analysis. TMM normalization was applied using the calcNormFactors function. Variability in the dataset was assessed with a MDSplot. The treatment and genotype factors were collapsed into one factor. Trended negative binomial dispersion parameters were estimated based on an additive no intercept model with the collapsed factor and a batch effect using the estimateDisp function and down-weighting outlying genes. A quasi-likelihood negative binomial regression model was then used to model the over dispersed counts for each gene separately as implemented in the function glmQLFit. The interest was in the simple tests of effect: testing for a treatment effect in the genotype and testing for a genotype effect under treatment conditions. Contrasts were estimated using empirical Bayes quasi-likelihood F-tests. P-values were corrected using FDR method described by Benjamini and Hochberg (1995) for each contrast separately. All edgeR functions were applied with default values. A gene was called differentially expressed when the FDR value was < 0.05. The differentially expressed genes used for further analysis were selected from all three timepoints compared to the 0h control.

### Plasmid construction and transformation

The constructs were generated on the basis of the Multisite Gateway system (Invitrogen) or Golden Gate system, as indicated in Data S4. For Multisite gateway construction, in order to generate transcriptional reporters, up to 4 kb promoters amplified using Col-0 genomic DNA as template were cloned into pDONRP4P1R entry vectors by BP reaction (see detailed information in Data S4). The entry vectors containing the promoter regions were next recombined into the binary vector pH7m34GW along with pDONR221-nYFP and pDONRP2RP3-GUS. Protein fusion reporters were obtained by cloning the respective promoter fragments into pDONRP4P1R, the respective coding sequence without stop codon into the entry clone pDONR221, and a fluorescence tag (GFP or YFP) into pH7m34GW destination vector using Gateway cloning. For p*RPS5A* driven misexpression constructs, coding sequences were PCR-amplified from root cDNA using Q5 polymerase (NEB, the Netherlands) (primers used are shown in Supplementary Data S4) and introduced into the pDONR221 or pGGC000 entry vectors. For the miR156-resistant SPL entry vector, we designed overlapping primers at the miR156 target site to change the coding sequence but not the amino acid sequence, and then used the miR156-sensitive SPL entry vector as a template to perform rolling circle PCR. All entry vectors were constructed into pK7m34GW,0 or pGGK_A-G destination vectors. All constructs were verified by Sanger sequencing and were transformed into wild-type Col-0 *Arabidopsis* plants using floral dipping (100). All primer sequences used for cloning and sequencing are listed in Data S4. The resulting constructs were transformed into *Agrobacterium tumefaciens* strain GV3101.

### Imaging and processing

GUS staining of the relative GUS lines was performed using GUS staining methods (49) and was observed using differential interference contrast (DIC) microscopy by mounting samples in a solution of 20% glycerol and 60% lactic acid, and imaged using an Olympus BX53 microscope equipped with DIC optics or APX100 microscopy. Cell wall staining for optical confocal cross sections was done using the ClearSee protocol including cell wall staining with 0.1% Calcofluor White. Confocal laser scanning microscopy was performed using a Leica SP8X device or Zeiss LSM900 device. Leica SP8X confocal was adapted with 20X or 40X water-corrected objectives (NA 1.2) using an argon laser excitation wavelength of 405 nm for Calcofluor White, 488nm for GFP, 514nm for YFP and 561nm for nuclear tdTomato (tdT)-and propium iodide (PI)-stained samples. Calcofluor White, GFP, YFP, tdT and PI were visualized at an emission of 425–475 nm, 500–535nm, 520–550nm, 580–630nm and 600–700nm, respectively. For histological sections, roots were fixed overnight and embedded as described previously (101). Images were then processed using Leica Application Suit X or Zen 3.6. All (optical) cross sections were made in the middle of the root apical meristem, defined by the quiescent center on one end and the first elongating cortex cell on the other end. This corresponds to a position in the root where typically one lateral root cap cell file is present. This visual cue was used in the histochemical cross section images to always find the same location in the root. Cell numbers were counted on these cross sections. For quantifications, we considered all cells derived from the provascular initial cells during embryogenesis as vascular cells; which thus comprises pericycle, xylem, phloem and procambium.

### Quantitative RT-PCR

Total RNAs were extracted from roots using Trizol reagent (Invitrogen) with RNeasy Kit (QIAGEN) as previously described (14) and reversely transcribed to first-strand complementary DNA by qScript cDNA SuperMix (QUANTABIO). qRT-PCR analysis was performed with SYBR GREEN I Master kit (Roche) and data were collected on a LightCycler480 apparatus (Roche) detection system in accordance with the manufacturer’s instructions. Data analysis was performed with Q-Base+ and transcript levels were normalized to *EEF1a4* and *CDKA1;1*. All qRT-PCR primers used in this study are listed in Data S4.

### Statistical analysis

All analyses were performed in R (version 4.0.3). All box plots were generated using ggplot2 (version 3.4.3). In all plots, center lines represent the medians; box limits indicate the 25th and 75th percentiles as determined by R software; whiskers extend 1.5 times the interquartile range from the 25th and 75th percentiles, individual measurements are represented by dots. The number of samples analyzed is indicated on the top of the x axis for each sample when relevant. Count data was analyzed by fitting generalized linear (mixed) models following poisson (stats package, version 4.0.3), quasi-poisson (stats package, version 4.0.3), negative binomial (stats package, version 4.0.3), or generalized poisson distribution (glmmTMB package, version 1.1.7). Continuous data was analyzed by fitting a linear model (stats package, version 4.0.3). Residual diagnostics of the models were performed using the simulation-based approach of the DHARMa package (version 0.4.6) to select the best fitting model. Inference from the models was done with the emmeans package (version 1.5.5-1) and p-values for the pairwise contrasts were adjusted using the Holm-Bonferroni method. Compact letter display of pairwise comparisons was generated using the multcomp package (version 1.4-16), using a significance level of 0.05. All data points and respective statistical analyses are summarized in Data S5.

## Supplementary Material

Data S1

Data S2

Data S3

Data S4

Data S5

Figures S1-24

## Data Availability

All quantitative data supporting the findings of this study and respective statistics are available as Data S5. Further information and requests for resources and reagents should be directed to Bert De Rybel (bert.derybel@psb.vib-ugent.be) or Baojun Yang (bjyang@genetics.ac.cn). Raw data for the bulk RNA-seq analysis can be accessed at NCBI with GEO number: GSE266817.

## One sentence summary

SPL13-dependent oriented cell divisions control the onset of a juvenile to adult phase change in the root apical meristem.
