# Supplementary material for: SPL13 controls a root apical meristem phase change by triggering oriented cell divisions
Source: Science. Author manuscript; Available in PMC 2025 May 15. (PMC7616863; doi:10.1126/science.ado4298)

# 1. General info for the synthetic protocols

Unless otherwise stated, all reactions were performed in oven-dried glassware, using a magnetically-stirred Teflon stir bar under argon atmosphere. All the reagents and solvents (HPLC grade) were used as without any further purification, with the exception of dichloromethane and triethylamine that have been distilled over $CaH_2$ prior to use, toluene which was distilled from sodium prior to use and THF which was distilled over sodium prior to use. $NaHCO_3$ that was used for quenching refers to a saturated solution in $H_2O$. Synthesized products and intermediates were stored under argon atmosphere and protected from light at -20 °C.

Reactions were monitored by thin layer chromatography (TLC), with SIL G25 $UV_{254}$ TLC plates with silica gel of 0.25 mm in thickness. Visualisation was obtained by irradiation with UV light (254 nm) and by staining with an anisaldehyde solution (5% para-anisaldehyde and 1% sulfuric acid in ethanol), a ceric ammonium molybdate solution (2.5% $(NH_4)_6Mo_7O_{24}.4H_2O$, 1% $Ce(NH_4)_4(SO_4)_4.2H_2O$ and 10% sulfuric acid in water), phosphomolybdic acid (PMA) solution (20 g PMA in 200 mL EtOH) or a potassium permanganate solution (3 g $KMnO_4$, 20 g $K_2CO_3$, 5 mL 5% aqueous NaOH and 300 mL $H_2O$). Column chromatography was performed with ROCC N.V. silica (particle size of from 0.060 to 0.200 mm). Technical grade pentane was used as the standard apolar mobile phase, typically enriched with a gradient of ethyl acetate.

$^{1}H$ Nuclear Magnetic Resonance (NMR) spectra were recorded with a resolution of 700, 500, 400 or 300 MHz. The 300 MHz measurements were performed on a 300 MHz Avance I (Bruker) with a 5 mm dual channel probe head - $^{1}H$ and Broadband (BBO-type) equipped with an ultrashield type magnet. Measurements with 400 MHz resolution were carried out on a 400 MHz Avance II (Bruker) with a 5 mm dual channel probe head - $^{1}H$ and Broadband (BBO-type) and an ultrashield type magnet. Samples measured with 500 MHz resolution were realised with the 500 MHz Avance II (Bruker) with a 5 mm triple channel probe head - $^{1}H$ $^{13}C$ $^{19}F$ (TXO type) provided with an Oxford type magnet. Measurements with 700 MHz resolution were caried out on the 700 MHz Avance II (Bruker) with a 5 mm triple channel probe head - $^{1}H$ $^{13}C$ $^{19}F$ (TXI type) and equipped with an Ultrashield + type magnet. The chemical shifts ($\delta$) are expressed in ppm and the residual solvent peak was used as the internal standard ($CDCl_3$: $\delta$H = 7.26 ppm; $\delta$C = 77.16 ppm, $C_6D_6$: $\delta$H = 7.16 ppm; $\delta$C = 128.06 ppm, Acetone-$d_6$: $\delta$H = 2.05 ppm; $\delta$C = 29.84 ppm,DCM-$d_2$: $\delta$H = 5.32 ppm; $\delta$C = 53.84 ppm,). Scalar couplings ($J$ ) are reported in Hertz (Hz). The multiplicity of the signals were designated by the following abbreviations: s, singlet; d, doublet; t, triplet; q, quadruplet; p, quintuplet; sx, sextet; sept, septuplet; m, multiplet; br broadened; band, several overlapping signals; AB, AB system with strongly skewed signals; app., apparent multiplicity of a signal.

LC-MS analysis was performed on an Agilent 1100 series HPLC connected to an Agilent G1956B single quadrupole MS, using an ESI-ionization source. A Phenomenex Kinetex C18 column (150 x 4.6 mm, particle size 5 µm) was used at 35 °C with a linear solvent gradient 100% A to 100%B in 6 min or 75% B to 100% B in 6 min (A: 5 mM NH4OH in H2O; B: MeCN). HRMS was recorded with direct injection by an Agilent 1100 Series HPLC on an Agilent 6220A time of flight HRMS using ESI/APCI-multimode ionization source. GCMS spectra were recorded on an Agilent 6890GC connected to an Agilent 5973 single quadrupole MS using EI ionization. The column used for the GCMS is an Agilent DB-5 (60 m x 0.25 mm, film thickness 0.25 µm) with He as carrier gas and going from 70 °C to 320 °C with a 17.5 °C/min gradient.

Infrared (IR) spectra were obtained with a Perkin-Elmer 1000 FT-IR spectrometer equipped with a HATR module. The intensity of the peaks were designated by the following abbreviations: s: strong (> 50% absorption); m: medium (between 20% and 50% absorption); w: weak (less then 20% absorption); br: broadened.

# 2. Synthetic protocols

## 2.1. 3-((2,2-dioxido-4-oxo-1,4-dihydro-3H-benzo[*d*][1,2]thiazin-3-yl)methyl)benzonitrile (3)

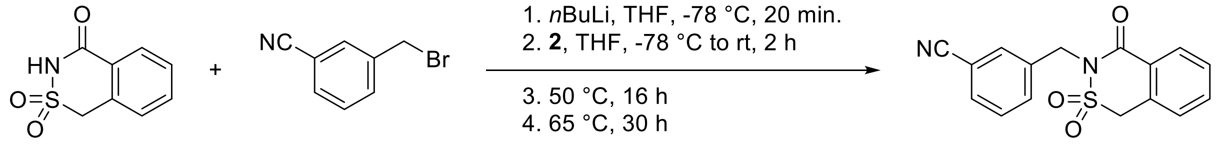


A solution of *n*-BuLi in hexane (2.5 M, 0.05 mL, 0.13 mmol, 1.25 equiv.) is slowly added to a solution of lactam **1** (21.2 mg, 0.11 mmol, 1 equiv.) in THF (0.55 mL), which is cooled to -78 °C. This solution is stirred for 20 minutes at -78 °C. Then, a solution of 3- (bromomethyl)benzonitrile **2** (21.0 mg, 0.11 mmol, 1 equiv.) in THF (0.55 mL) is slowly added to the lithiated amide solution. The resulting solution is allowed to warm to room temperature over a period of 2 h and is subsequently heated to 50 °C for a period of 16 h. After a period of 16 h the conversion rate is checked via TLC analysis (30% EtOAc in petroleum ether). Only a limited conversion can be observed, therefore it is decided to increase the temperature to 65 °C. After 30 h, the reaction mixture is cooled to room temperature and quenched with a saturated aqueous solution of ammonium chloride (1.5 mL), even though there is no complete conversion of the starting materials. The aqueous phase is extracted with DCM (3 × 15 mL) and the combined organic phases are dried over anhydrous magnesium sulphate. The solvents are removed under reduced pressure and gradient flash chromatography (eluent: 20% to 25% EtOAc in petroleum ether) gives enriched fractions of the title compound **3**. Reversed-phase preparative HPLC of these enriched fractions (0 to 100% MeCN in water with 0.1% TFA; gradient: 5%/min over 5 min followed by 2%/min over 5 min followed by 1%/min over 30 min followed by 5%/min over 7 min; the product elutes at ~ 30.1 min) eventually gives pure title compound **3** (12.3 mg, 37%) as a white solid. A conversion of 57% for title compound **3** was deduced from the crude $^{1}H$ NMR spectrum.

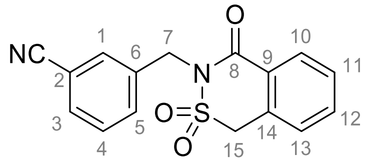

**Formula:** $C_{16}H_{12}N_2O_3S$
**MW:** 312.343 g/mol
**$R_f$ (20% EtOAc in petroleum ether):** 0.21
**IR: $\nu_{max}$ (cm$^{-1}$)**: 2971 (w), 2226 (m), 1679 (s), 1338 (s), 1283 (s), 1139 (s), 1046 (m), 795 (m), 735 (s)
**HRMS (ESI)**: calcd for $C_{16}H_{13}N_2O_3S^+$ [M+H]+: 313.06414; found: 313.0647
**$^{1}H$ NMR (400 MHz, $CDCl_3$):** $\delta$8.23 (dd, *J* = 7.5, 1.2 Hz, 1H, *C10H*), 7.82 (s(br) 1H, *C1H*), 7.76 (d(br), *J* = 7.7 Hz, 1H, *C5H*), 7.65 (td, *J* = 7.5, 1.5 Hz, 1H, *C12H*), 7.59 (d(br), *J* = 7.7 Hz, 1H, *C3H*), 7.58 (td, *J* = 7.5, 1.1 Hz, 1H, *C11H*), 7.46 (t, *J* = 7.7 Hz, 1H, *C4H*), 7.34 (d(br), *J* = 7.5 Hz, 1H, *C13H*), 5.12 (s, 2H, *C7H*$_2$), 4.76 (s, 2H, *C15H*$_2$)
**$^{13}C$ NMR (100 MHz, $CDCl_3$):** $\delta$163.7 (C), 137.6 (C), 134.3 (CH), 133.4 (CH), 132.4 (CH), 131.7 (CH), 131.0 (CH), 129.8 (CH), 129.5 (CH), 129.2 (CH), 128.0 (C), 126.8 (C), 118.5 (C), 112.8 (C), 54.6 ($CH_2$), 43.5 ($CH_2$)
**HSQC:** 134.3 × 7.65, 133.4 × 7.76, 132.4 × 7.82, 131.7 × 7.59, 131.0 × 8.23, 129.8 × 7.58, 129.5 × 7.46, 129.2 × 7.34, 54.6 × 4.67, 43.5 × 5.12

## 2.2. 3-(4-bromobenzyl)-1H-benzo[*d*][1,2]thiazin-4(3*H*)-one 2,2-dioxide (5)

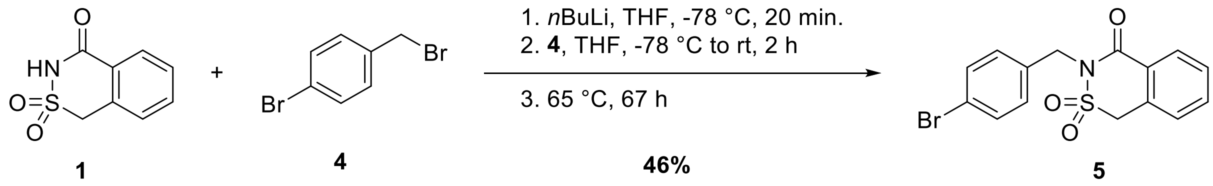


A solution of lactam **1** (20.0 mg, 0.10 mmol, 1 equiv.) in THF (0.5 mL) is cooled to -78 °C and a solution *n*-BuLi in hexane (2.5 M, 0.05 mL, 0.13 mmol, 1.25 equiv.) is added dropwise to this solution. The resulting mixture is stirred for 20 minutes at -78 °C. Then, a solution of 1-bromo-4-(bromomethyl)benzene **4** (25.2 mg, 0.10 mmol, 1 equiv.) in THF (0.5 mL) is slowly added to the lithiated amide solution. The resulting solution is allowed to warm to room temperature over a period of 2 h and is subsequently heated to 65 °C for a period of 67 h. In the meantime, the conversion rate of the reaction is regularly monitored via TLC analysis (30% EtOAc in petroleum ether). After 67 h, the cooled reaction mixture is quenched with a saturated aqueous solution of ammonium chloride (2 mL). The aqueous phase is extracted with DCM (3 × 20 mL) and the combined organic phases are dried over anhydrous magnesium sulphate. Removal of the solvents in vacuo and purification of the crude compound via gradient flash chromatography (eluent: 20% to 25% EtOAc in petroleum ether) gives title compound **5** (17.1 mg, 46%) as a yellowish white solid. A conversion rate of 72% for title compound **5** was deduced from the crude $^{1}$H NMR spectrum.

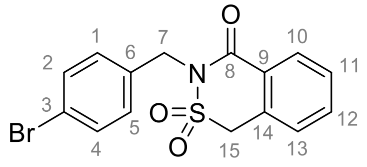

**Formula:** $C_{15}H_{12}BrNO_3S$
**MW:** 366.229 g/mol
**$R_f$ (20% EtOAc in Petroleum ether):** 0.17
**IR:** $\nu_{\max}$ (cm$^{-1}$): 2918 (w), 1677 (s), 1345 (s), 1282 (s), 1165 (s), 1138 (s), 1046 (m), 858 (m), 836 (m), 734 (s), 688 (m)
**HRMS (ESI):** calcd for $C_{15}H_{13}BrNO_3S^+$ $[M+H]^+$: 365.97940; found: 365.9800
**$^{1}$H NMR (300 MHz, CDCl$_3$):** $\delta$8.21 (dd, *J* = 7.5, 1.5 Hz, 1H, *C10H*), 7.63 (dt, *J* = 7.5, 1.5 Hz, 1H, *C12H*), 7.56 (dt, *J* = 7.5, 1.5 Hz, 1H, *C11H*), 7.46 (dt(AB), *J* = 8.7, 2.1 Hz, 2H, *C2H* and *C4H*), 7.40 (dt(AB), *J* = 8.7, 2.1 Hz, 2H, *C1H* and *C5H*), 7.32 (d(br), *J* = 7.5 Hz, 1H, *C13H*), 5.06 (s, 2H, *C7H$_2$*), 4.63 (s, 2H, *C15H$_2$*)
**$^{13}$C NMR (75 MHz, CDCl$_3$):** $\delta$163.7 (C), 135.1 (C), 134.1 (CH), 131.7 (CH), 130.9 (CH), 130.7 (CH), 129.7 (CH), 129.1 (CH), 128.0 (C), 127.0 (C), 122.1 (C), 54.6 (CH$_2$), 43.8 (CH$_2$)
**HSQC:** 134.1 × 7.63, 131.7 × 7.46, 130.9 × 8.21, 130.7 × 7.40, 129.7 × 7.56, 129.1 × 7.32, 54.6 × 4.63, 43.8 × 5.06

## 2.3. 3-(3,5-dimethylbenzyl)-1H-benzo[*d*][1,2]thiazin-4(3*H*)-one 2,2-dioxide (7)

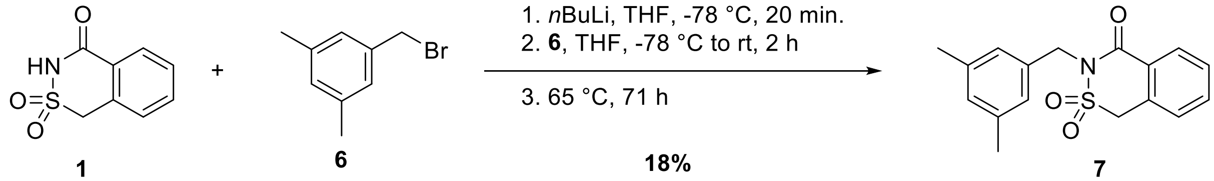


**1** **6** **18%** **7**

A solution of *n*-BuLi in hexane (2.5 M, 0.05 mL, 0.13 mmol, 1.25 equiv.) is slowly added to a solution of lactam **1** (20.0 mg, 0.10 mmol, 1 equiv.) in THF (0.5 mL), which is cooled to -78 °C. This solution is stirred for 20 minutes at -78 °C. Then, a solution of 1-(bromomethyl)-3,5-dimethylbenzene **6** (18.7 mg, 0.10 mmol, 1 equiv.) in THF (0.5 mL) is slowly added to the lithiated amide solution. The resulting solution is warmed to room temperature over a period of 2 h and is subsequently heated to 65 °C for a period of 71 h. During this period, the conversion rate is regularly checked via TLC analysis (30% EtOAc in petroleum ether). Even though only a small amount of new spots can be observed via TLC analysis, it is still decided to terminate the reaction. The reaction mixture is cooled to room temperature and quenched with a saturated aqueous solution of ammonium chloride (1.5 mL). The aqueous phase is extracted with DCM (3 × 15 mL) and the combined organic phases are dried over anhydrous magnesium sulphate. The organic solvents are removed via evaporation and the crude mixture is purified via flash chromatography (eluent: 20% EtOAc in petroleum ether) yielding title compound **7** (5.6 mg, 18%) as a white solid. A conversion rate of 27% for title compound **7** was deduced from the crude $^{1}H$ NMR spectrum.

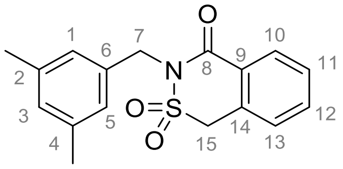

**Formula:** C17H17NO3S
**MW:** 315.387 g/mol
**$R_f$ (20% EtOAc in petroleum ether):** 0.24
**IR:** $\nu_{max}$(cm$^{-1}$): 2922 (w), 1682 (s), 1345 (s), 1278 (s), 1162 (s), 1139 (s), 1046 (m), 860(m), 740 (s), 720 (m)
**HRMS (ESI):** calcd for $C_{17}H_{18}NO_3S^+$ [M+H]$^+$: 316.10019; found: 316.0998
**$^{1}H$ NMR (400 MHz, $CDCl_3$):** $\delta$8.23 (dd, *J* = 7.6, 1.4 Hz, 1H, *C10H*), 7.62 (td, *J* = 7.6, 1.4 Hz, 1H, *C12H*), 7.55 (td, *J* = 7.6, 1.4 Hz, 1H, *C11H*), 7.32 (d(br), *J* = 7.6 Hz, 1H, *C13H*), 7.11 (s(br), 2H, *C1H* and *C5H*), 6.91 (s(br), 1H, *C3H*), 5.05 (s, 2H, *C7H*$_2$), 4.63 (s, 2H, *C15H*$_2$), 2.30 (s, 6H, C2-*CH*$_3$ and C4-*CH*$_3$)
**$^{13}C$ NMR (100 MHz, $CDCl_3$):** $\delta$163.7 (C), 138.1 (C), 136.0 (C), 133.9 (CH), 130.9 (CH), 129.6 (CH), 129.0 (CH), 128.2 (C), 127.3 (C), 126.5 (CH), 54.6 ($CH_2$), 44.4 ($CH_2$), 21.3 ($CH_3$)
**HSQC:** 133.9 × 7.62, 130.9 × 8.23, 129.6 × 7.55, 129.6 × 6.91, 129.0 × 7.32, 126.5 × 7.11, 54.6 × 4.63, 44.4 × 5.05, 21.3 × 2.30

## 2.4. 3-(3-bromobenzyl)-1H-benzo[*d*][1,2]thiazin-4(3*H*)-one 2,2-dioxide (9)

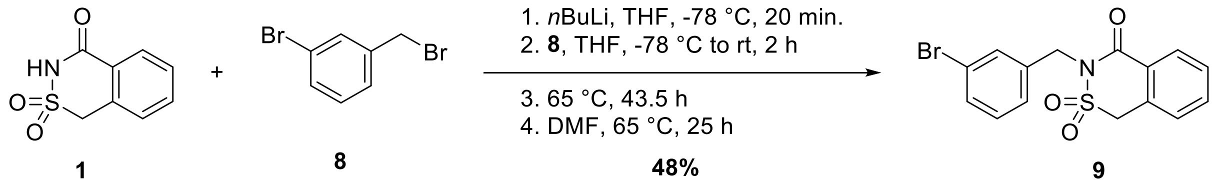


To a solution of lactam **1** (20.0 mg, 0.10 mmol, 1 equiv.) in THF (0.5 mL), cooled to -78 °C, a solution of *n*-BuLi in hexane (2.5 M, 0.05 mL, 0.13 mmol, 1.25 equiv.) is slowly added. This solution is stirred for 20 minutes at -78 °C. Then, a solution of 1-bromo-3- (bromomethyl)benzene **8** (25.2 mg, 0.10 mmol, 1 equiv.) in THF (0.5 mL) is slowly added to the lithiated amide solution. The resulting solution is warmed to room temperature over a period of 2 h and is subsequently heated to 65 °C for a period of 43 h. In the meantime the conversion rate is monitored via TLC (30% EtOAc in petroleum ether). After 43 h, no conversion can be observed, therefore DMF (0.2 mL) is added. The reaction mixture changes from a milky, turbid solution to a clear, transparent solution. The resulting mixture is stirred for 25 h at 65 °C. After 25 h, no starting material **8** can be detected anymore, therefore the reaction mixture is cooled to room temperature and quenched with a saturated aqueous solution of ammonium chloride (1 mL). The aqueous phase is extracted with DCM (3 × 15 mL) and the combined organic phases are dried over anhydrous magnesium sulphate. Removal of the solvents in vacuo and purification of the crude compound via flash chromatography (eluent: 20% EtOAc in petroleum ether) gives title compound **9** (17.8 mg, 48%) as a white solid.

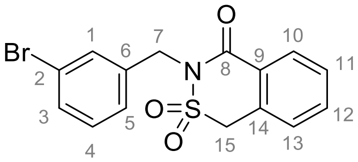

**Formula:** $C_{15}H_{12}BrNO_3S$
**MW:** 366.229 g/mol
**$R_f$ (30% EtOAc in petroleum ether):** 0.35
**IR:** $\nu_{max}$(cm$^{-1}$): 2923 (w), 1666 (s), 1355 (s), 1284 (m), 1154 (m), 1043 (m), 786 (m), 739 (s), 712 (s), 688 (m)
**HRMS (ESI):** calcd for $C_{15}H_{13}BrNO_3S^+$ $[M+H]^+$: 365.97940; found: 365.9778
**$^1$H NMR (300 MHz, CDCl$_3$):** $\delta$8.23 (dd, *J* = 7.5, 1.5 Hz, 1H, *C10H*), 7.66 (s, 1H, *C1H*), 7.64 (td, *J* = 7.5, 1.5 Hz, 1H, *C12H*), 7.56 (td, *J* = 7.5, 1.5 Hz, 1H, *C11H*), 7.45 (d(br), *J* = 7.8 Hz, 1H, *C5H*), 7.42 (d(br), *J* = 7.8 Hz, 1H, *C3H*), 7.33 (d(br), *J* = 7.5 Hz, 1H, *C13H*), 7.21 (t, *J* = 7.8 Hz, 1H, *C4H*), 5.07 (s, 2H, *C7H$_2$*), 4.65 (s, 2H, *C15H$_2$*)
**$^{13}$C NMR (75 MHz, CDCl$_3$):** $\delta$163.7 (C), 138.3 (C), 134.2 (CH), 131.8 (CH), 131.2 (CH), 131.0 (CH), 130.1 (CH), 129.7 (CH), 129.1 (CH), 128.1 (C), 127.5 (CH), 127.0 (C), 122.6 (C), 54.6 (CH$_2$), 43.7 (CH$_2$)
**HSQC:** 134.2 × 7.64, 131.8 × 7.66, 131.2 × 7.42, 131.0 × 8.23, 130.1 × 7.21, 129.7 × 7.56, 129.1 × 7.33, 127.5 × 7.45, 54.6 × 4.65, 43.7 × 5.07

## 2.5. 3-(2-bromobenzyl)-1H-benzo[*d*][1,2]thiazin-4(3*H*)-one 2,2-dioxide (11)

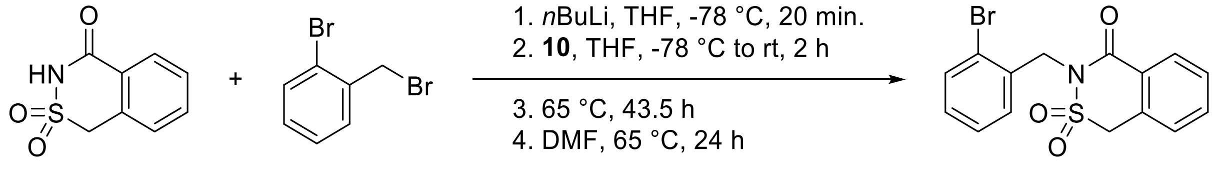


**1** **10** **48%** **11**

To a solution of lactam **1** (20.0 mg, 0.10 mmol, 1 equiv.) in THF (0.5 mL), cooled to -78 °C, a solution of *n*-BuLi in hexane (2.5 M, 0.05 mL, 0.13 mmol, 1.25 equiv.) is slowly added. This solution is stirred for 20 minutes at -78 °C. Then, a solution of 1-bromo-2-(bromomethyl)benzene **10** (25.2 mg, 0.10 mmol, 1 equiv.) in THF (0.5 mL) is slowly added to the lithiated amide solution. The resulting solution is warmed to room temperature over a period of 2 h and is subsequently heated to 65 °C for a period of 43 h. In the meantime the conversion rate is regularly checked via TLC (30% EtOAc in petroleum ether). After 43 h, no conversion can be observed, therefore DMF (0.2 mL) is added. The reaction mixture changes from a milky, turbid solution to a clear, transparent solution. The resulting mixture is stirred for 24 h at 65 °C. After 24 h, no starting material **10** can be observed anymore, therefore the cooled reaction mixture is quenched with a saturated aqueous solution of ammonium chloride (1 mL). The aqueous phase is extracted with DCM (3 × 15 mL) and the combined organic phases are dried over anhydrous magnesium sulphate. Removal of the solvents in vacuo and purification of the crude compound via gradient flash chromatography (eluent: 20% to 25% EtOAc in petroleum ether) gives title compound **11** (20.7 mg, 62%) as a white solid.

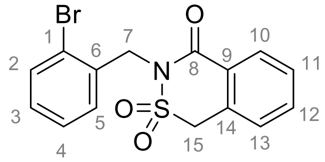

**Formula:** $C_{15}H_{12}BrNO_3S$
**MW:** 366.229 g/mol
**$R_f$ (30% EtOAc in petroleum ether):** 0.22
**IR:** $\nu_{max}$(cm$^{-1}$): 2921 (w), 1687 (s), 1316 (s), 1286 (s), 1169 (s), 1022 (m), 877 (m), 735 (s), 688 (m)
**HRMS (ESI):** calcd for $C_{15}H_{13}BrNO_3S^+$ [M+H]$^+$: 365.97940; found: 365.9780
**$^1$H NMR (300 MHz, CDCl$_3$):** $\delta$8.24 (dd, *J* = 7.5, 1.5 Hz, 1H, *C10H*), 7.67 (td, *J* = 7.5, 1.5 Hz, 1H, *C12H*), 7.59 (td, *J* = 7.5, 1.5 Hz, 1H, *C11H*), 7.58 (dd, *J* = 7.5, 1.1 Hz, 1H, *C2H*), 7.37 (d(br), *J* = 7.5 Hz, 1H, *C13H*), 7.32 - 7.23 (band, 2H, *C4H* and *C5H*), 7.13 (td(br), *J* = 7.5, 2.4 Hz, 1H, *C3H*), 5.25 (s, 2H, *C7H$_2$*), 4.72 (s, 2H, *C15H$_2$*)
**$^{13}$C NMR (75 MHz, CDCl$_3$):** $\delta$163.7 (C), 134.8 (C), 134.2 (CH), 132.9 (CH), 131.1 (CH), 129.8 (CH), 129.1 (CH), 128.9 (CH), 128.2 (C), 127.7 (CH), 127.6 (CH), 127.1 (C), 122.4 (C), 54.6 (CH$_2$), 45.3 (CH$_2$)
**HSQC:** 134.2 × 7.67, 132.9 × 7.58, 131.1 × 8.24, 129.8 × 7.59, 129.1 × 7.37, 128.9 ×7.13, 127.7 × 7.32 - 7.23, 127.6 × 7.32 - 7.23, 54.6 × 4.72, 45.3 × 5.25

## 2.6. 3-(2-methylbenzyl)-1H-benzo[*d*][1,2]thiazin-4(3*H*)-one 2,2-dioxide (13)

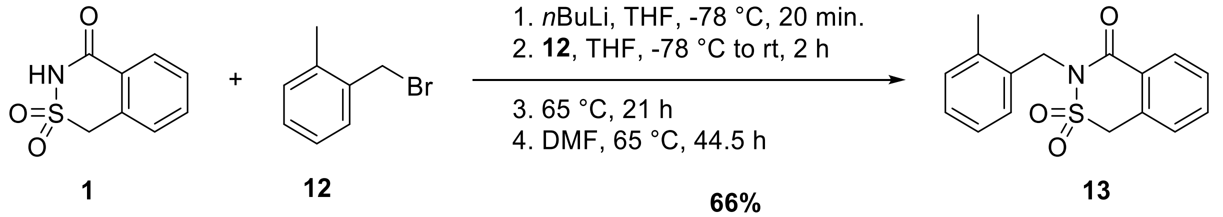


To a solution of lactam **1** (20.0 mg, 0.10 mmol, 1 equiv.) in THF (0.5 mL), cooled to -78 °C, a solution of *n*-BuLi in hexane (2.5 M, 0.05 mL, 0.13 mmol, 1.25 equiv.) is slowly added. This solution is stirred for 20 minutes at -78 °C. Then, a solution of 1-(bromomethyl)-2-methylbenzene **12** (18.7 mg, 0.10 mmol, 1 equiv.) in THF (0.5 mL) is slowly added to the lithiated amide solution. The resulting solution is allowed to warm to room temperature over a period of 2 h and is subsequently heated to 65 °C for a period of 21 h. After a period of 21 h the conversion rate is checked via TLC analysis (30% EtOAc in petroleum ether). No conversion can be observed, therefore DMF (0.2 mL) is added. The reaction mixture changes from a milky, turbid solution to a clear, transparent solution. The resulting mixture is stirred for 44.5 h at 65 °C. After 44.5 h, no starting material **12** can be detected anymore, therefore the reaction mixture is cooled to room temperature and quenched with a saturated aqueous solution of ammonium chloride (1 mL). The aqueous phase is extracted with DCM (3 × 15 mL) and the combined organic phases are dried over anhydrous magnesium sulphate. Removal of the solvents in vacuo and purification of the crude compound via flash chromatography (eluent: 20% EtOAc in petroleum ether) gives title compound **13** (20.1 mg, 66%) as a white solid.

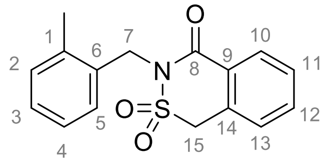

**Formula:** $C_{16}H_{15}NO_3S$
**MW:** 301.360 g/mol
**R$_f$ (20% EtOAc in petroleum ether):** 0.20
**IR:** $\nu_{max}$(cm$^{-1}$): 2924 (m), 1685 (s), 1346 (s), 1317 (s), 1282 (s), 1167 (s), 1137 (s), 736(s)
**HRMS (ESI):** calcd for $C_{16}H_{16}NO_3S^+$ [M+H]$^+$: 302.08454; found: 302.0850
**$^1$H NMR (300 MHz, CDCl$_3$):** $\delta$8.23 (dd, *J* = 7.5, 1.5 Hz, 1H, *C10H*), 7.64 (td, *J* = 7.5, 1.5 Hz, 1H, *C12H*), 7.57 (td, *J* = 7.5, 1.5 Hz, 1H, *C11H*), 7.35 (d(br), *J* = 7.5 Hz, 1H, *C13H*), 7.32 - 7.27 (m, 1H, *C5H*), 7.19 - 7.14 (band, 3H, *C2H*, *C3H* and *C4H*), 5.15 (s, 2H, *C7H$_2$*), 4.69 (s, 2H, *C15H$_2$*), 2.46 (s, 3H, C1-*CH$_3$*)
**$^{13}$C NMR (75 MHz, CDCl$_3$):** $\delta$163.8 (C), 135.6 (C), 134.0 (CH), 133.9 (C), 131.0 (CH), 130.4 (CH), 129.7 (CH), 129.0 (CH), 128.2 (C), 127.5 (CH), 127.2 (C), 126.8 (CH), 126.2 (CH), 54.5 (CH$_2$), 42.1 (CH$_2$), 19.3 (CH$_3$)
**HSQC:** 134.0 × 7.64, 131.0 × 8.23, 130.4 × 7.19 - 7.14, 129.7 × 7.54, 129.0 × 7.35, 127.5 × 7.19 - 7.14, 126.8 × 7.32 - 7.27, 126.2 × 7.19 - 7.14, 54.5 × 4.69, 42.1 × 5.15, 19.3 × 2.46

## 2.7. 3-(3-methylbenzyl)-1H-benzo[*d*][1,2]thiazin-4(3*H*)-one 2,2-dioxide (15)

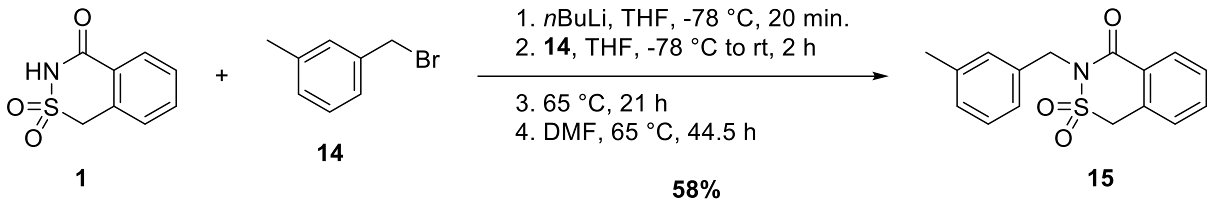


A solution of *n*-BuLi in hexane (2.5 M, 0.05 mL, 0.13 mmol, 1.25 equiv.) is slowly added to a solution of lactam **1** (20.0 mg, 0.10 mmol, 1 equiv.) in THF (0.5 mL), which is cooled to -78 °C. This solution is stirred for 20 minutes at -78 °C. Then, a solution of 1-(bromomethyl)-3-methylbenzene **14** (18.7 mg, 0.13 mmol, 1 equiv.) in THF (0.5 mL) is added dropwise to the lithiated amide solution. The resulting solution is allowed to warm to room temperature over a period of 2 h and is subsequently heated to 65 °C for a period of 21 h. After a period 21 h the conversion rate is checked via TLC analysis (30% EtOAc in petroleum ether). A complete lack of conversion can be deduced from the TLC analysis, therefore, DMF (0.2 mL) is added to push the reaction to completion. The reaction mixture changes from a milky, turbid solution to a clear, transparent solution. This mixture is stirred for another 44 h at 65 °C. After 44 h, no starting material **14** can be detected anymore, therefore the reaction mixture is cooled to room temperature and subsequently quenched with a saturated aqueous solution of ammonium chloride (1.5 mL). The aqueous phase is extracted with DCM (3 × 15 mL) and the combined organic phases are dried over anhydrous magnesium sulphate. Evaporation of the solvents under reduced pressure and purification of the crude compound via gradient flash chromatography (eluent: 20% to 30% EtOAc in petroleum ether) gives title compound **15** (18.3 mg, 58%) as a white solid.

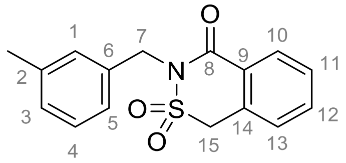

**Formula:** $C_{16}H_{15}NO_3S$
**MW:** 301.360 g/mol
**R$_f$ (30% EtOAc in petroleum ether):** 0.35
**IR:** $\nu_{max}$(cm$^{-1}$): 2921 (w), 1666 (s), 1351 (s), 1286 (m), 1155 (m), 1042 (m), 792 (m), 740 (s), 709 (s)
**HRMS (ESI):** calcd for $C_{16}H_{16}NO_3S^+$ [M+H]$^+$: 302.08454; found: 302.0836
**$^1$H NMR (300 MHz, CDCl$_3$):** $\delta$8.23 (dd, *J* = 7.5, 1.5 Hz, 1H, *C10H*), 7.62 (td, *J* = 7.5, 1.5 Hz, *C12H*), 7.55 (td, *J* = 7.5, 1.5 Hz, 1H, *C11H*), 7.34 - 7.28 (band, 3H, *C1H*, *C5H* and *C13H*), 7.22 (t, *J* = 7.5 Hz, 1H, *C4H*), 7.09 (d(br), *J* = 7.5 Hz, 1H, *C3H*), 5.09 (s, 2H, *C7H$_2$*), 4.63 (s, 2H, *C15H$_2$*), 2.34 (s, 3H, C2-*CH$_3$*)
**$^{13}$C NMR (75 MHz, CDCl$_3$):** $\delta$163.7 (C), 138.2 (C), 136.0 (C), 134.0 (CH), 130.9 (CH), 129.6 (CH), 129.5 (CH), 129.0 (CH), 128.7 (CH), 128.5 (CH), 128.1 (C), 127.3 (C), 125.8 (CH), 54.6 (CH$_2$), 44.5 (CH$_2$), 21.4 (CH$_3$)
**HSQC:** 134.0 × 7.62, 130.9 × 8.23, 129.6 × 7.55, 129.5 × 7.34 - 7.28, 129.0 × 7.34 - 7.28, 128.7 × 7.09, 128.5 × 7.22, 125.8 × 7.34 - 7.28, 54.6 × 4.63, 44.5 × 5.09, 21.4 × 2.34

## 2.8. 3-(2-methoxybenzyl)-1H-benzo[*d*][1,2]thiazin-4(3*H*)-one 2,2-dioxide (17)

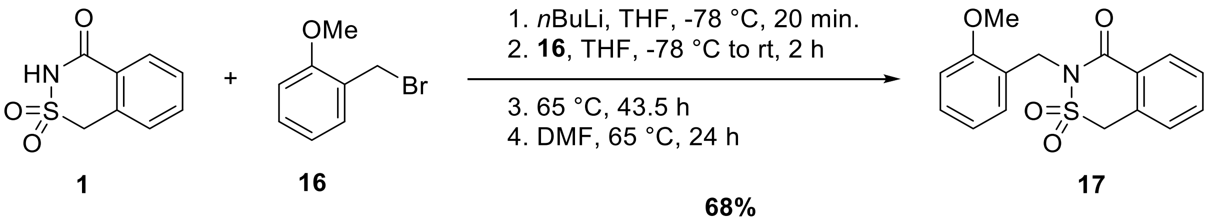


A solution of *n*-BuLi in hexane (2.5 M, 0.05 mL, 0.13 mmol, 1.25 equiv.) is slowly added to a solution of lactam **1** (20.0 mg, 0.10 mmol, 1 equiv.) in THF (0.5 mL), which is cooled to -78 °C. This mixture is stirred for 20 minutes at -78 °C. Next, a solution of 1-(bromomethyl)-2-methoxybenzene **16** (20.3 mg, 0.10 mmol, 1 equiv.), in THF (0.5 mL) is slowly added to the lithiated amide solution. The resulting solution is warmed to room temperature over a period of 2 h and is subsequently heated to 65 °C for a period of 43 h. The conversion rate is regularly checked via TLC (30% EtOAc in petroleum ether). After 43 h, no new spots can be observed via TLC, therefore DMF (0.2 mL) is added. The reaction mixture changes from a milky, turbid solution to a clear, transparent solution. The resulting mixture is stirred for 24 h at 65 °C. After 24 h, complete conversion can be observed via TLC, therefore the reaction is terminated through the quick addition of a saturated aqueous solution of ammonium chloride (1 mL). The aqueous phase is extracted with DCM (3 × 15 mL) and the combined organic phases are dried over anhydrous magnesium sulphate. Removal of the organic solvents under reduced pressure and purification of the crude compound via flash chromatography (eluent: 20% EtOAc in petroleum ether) gives title compound **17** (21.7 mg, 68%) as a white solid.

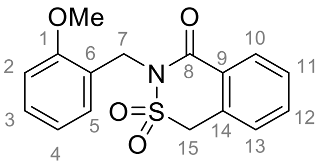

**Formula:** $C_{16}H_{15}NO_4S$
**MW:** 317.359 g/mol
**$R_f$ (30% EtOAc in petroleum ether):** 0.31
**IR:** $\nu_{max}$(cm$^{-1}$): 2924 (w), 1687 (s), 1312 (s), 1298 (s), 1282 (s), 1241 (s), 1162 (s), 1135 (s), 1109 (s), 1063 (s), 1021 (s), 964 (m), 794 (m), 761 (s), 740 (s), 702 (m)
**HRMS (ESI):** calcd for $C_{16}H_{16}NO_4S^+$ [M+H]$^+$: 318.07946; found: 318.0810
**$^1$H NMR (300 MHz, CDCl$_3$):** $\delta$8.21 (dd, *J* = 7.5, 1.5 Hz, 1H, *C10H*), 7.63 (td, *J* = 7.5, 1.5 Hz, 1H, *C12H*), 7.55 (td, *J* = 7.5, 1.5 Hz, 1H, *C11H*), 7.34 (d(br), *J* = 7.5 Hz, 1H, *C13H*), 7.29 (d(br), *J* = 7.7 Hz, 1H, *C5H*), 7.25 (td, *J* = 7.7, 1.7 Hz, 1H, *C3H*), 6.91 (td, *J* = 7.7, 0.9 Hz, 1H, *C4H*), 6.88 (d(br), *J* = 7.7 Hz, 1H, *C2H*), 5.19 (s, 2H, *C7H$_2$*), 4.66 (s, 2H, *C15H$_2$*), 3.87 (s, 3H, O-*CH$_3$*)
**$^{13}$C NMR (75 MHz, CDCl$_3$):** $\delta$163.8 (C), 157.0 (C), 133.9 (CH), 130.9 (CH), 129.6 (CH), 129.0 (CH), 128.7 (CH), 128.3 (CH), 128.3 (C), 127.5 (C), 124.0 (C), 120.4 (CH), 110.4 (CH), 55.4 ($CH_3$), 54.5 ($CH_2$), 40.5 ($CH_2$)
**HSQC:** 133.9 × 7.63, 130.9 × 8.21, 129.6 × 7.55, 129.0 × 7.34, 128.7 × 7.25, 128.3 × 7.29, 120.4 × 6.91, 110.4 × 6.88, 55.4 × 3.87, 54.5 × 4.66, 40.5 × 3.87

## 2.9. 3-benzyl-1H-benzo[*d*][1,2]thiazin-4(3*H*)-one 2,2-dioxide (19)

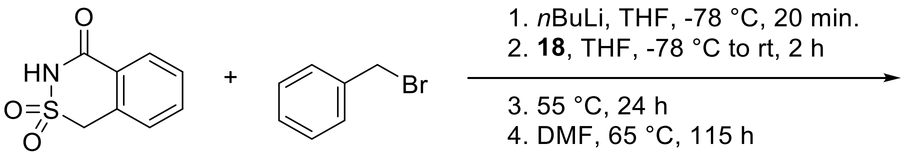


**1** **18** **19**

**41%**

A solution of *n*-BuLi in hexane (2.5 M, 0.05 mL, 0.13 mmol, 1.25 equiv.) is added dropwise to a solution of lactam **1** (20.0 mg, 0.10 mmol, 1 equiv.) in THF (0.5 mL), which is cooled to -78 °C. This solution is stirred for 20 minutes at -78 °C. Then, a solution of benzyl bromide **18** (17.3 mg, 0.10 mmol, 1 equiv.) in THF (0.5 mL) is added dropwise to the lithiated amide solution. The resulting solution is allowed to warm to room temperature over a period of 2 h and is subsequently heated to 55 °C for a period of 24 h. After a period 24 h the conversion rate is checked via TLC analysis (30% EtOAc in petroleum ether). No new compounds can be observed via TLC analysis, therefore, DMF (0.2 mL) is added to push the reaction to completion. The reaction mixture changes from a milky, turbid solution to a clear, transparent solution. This mixture is stirred for another 115 h at 65 °C. After 115 h, no starting material **18** can be detected anymore, therefore the reaction mixture is cooled to room temperature and subsequently quenched with a saturated aqueous solution of ammonium chloride (1.5 mL). The aqueous phase is extracted with DCM (3 × 15 mL). The combined organic phases are washed with brine (6 mL) and dried over anhydrous magnesium sulphate. Removal of the solvents in vacuo and purification of the crude compound via gradient flash chromatography (eluent: 15% to 30% EtOAc in petroleum ether) yields title compound **19** (11.8 mg, 41%) as a white solid.

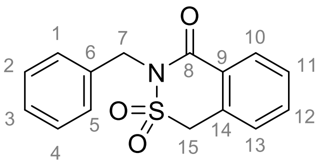


**Formula:** $C_{15}H_{13}NO_3S$
**MW:** 287.333 g/mol
**$R_f$ (30% EtOAc in petroleum ether):** 0.42
**IR:** $\nu_{max}$(cm$^{-1}$): 2921 (w), 1666 (s), 1351 (s), 1314 (s), 1154 (s), 1031 (m), 760 (m), 738(s), 701 (s), 690 (s)
**HRMS (ESI):** calcd for $C_{15}H_{14}NO_3S^+$ [M+H]$^+$: 288.06889; found: 288.0685
**$^1$H NMR (300 MHz, CDCl$_3$):** $\delta$8.22 (dd, *J* = 7.6, 1.5 Hz, 1H, *C10H*), 7.62 (td, *J* = 7.6, 1.5 Hz, 1H, *C12H*), 7.55 (td, *J* = 7.6, 1.5 Hz, 1H, *C11H*), 7.54 - 7.49 (band, 2H, 2 × CHAr), 7.38 - 7.29 (band, 1H, *C13H*), 7.38 - 7.29 (band, 2H, 2 × CHAr), 7.32 - 7.26 (band, 1H, CHAr), 5.13 (s, 2H, *C7H*$_2$), 4.63 (s, 2H, *C15H*$_2$)
**$^{13}$C NMR (75 MHz, CDCl$_3$):** $\delta$163.7 (C), 136.1 (C), 134.0 (CH), 130.9 (CH), 129.6 (CH), 129.0 (CH), 128.8 (CH), 128.6 (CH), 128.1 (C), 127.9 (CH), 127.2 (C), 54.6 (CH$_2$), 44.5 (CH$_2$)
**HSQC:** 134.0 × 7.62, 130.9 × 8.22, 129.6 × 7.55, 129.0 × 7.54 - 7.49, 128.8 × 7.54 - 7.49, 128.6 × 7.38 - 7.29, 127.9 × 7.32 - 7.26, 54.6 × 4.63, 44.5 × 5.13

## 2.10. 3-(4-ethynylbenzyl)-1H-benzo[*d*][1,2]thiazin-4(3*H*)-one 2,2-dioxide (21)

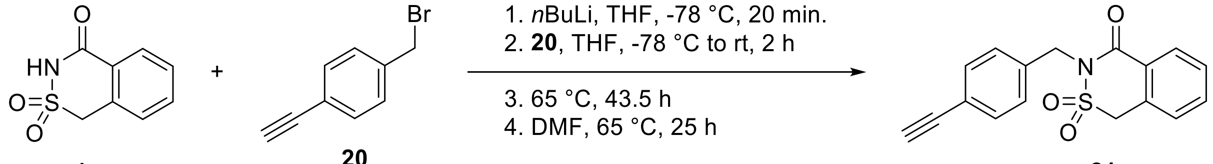


**1** **20** **71%** **21**

To a solution of lactam **1** (24.0 mg, 0.12 mmol, 1 equiv.) in THF (0.6 mL), which is cooled to -78 °C, a solution of *n*-BuLi in hexane (2.5 M, 0.06 mL, 0.15 mmol, 1.25 equiv.) is slowly added. The solution is stirred for 20 minutes at -78 °C. Then, a solution of 1-(bromomethyl)-4-ethynylbenzene **20** (23.6 mg, 0.12 mmol, 1 equiv.), in THF (0.6 mL) is slowly added to the lithiated amide solution. The resulting solution is allowed to warm to room temperature over a period of 2 h and is subsequently heated to 65 °C for a period of 43 h. After a period 43 h the conversion rate is checked via TLC analysis (30% EtOAc in petroleum ether). A complete lack of conversion can be deduced from the TLC, therefore DMF (0.2 mL) is added to push the reaction to completion. The reaction mixture changes from a milky, turbid solution to a clear, transparent solution. This mixture is stirred for another 25 h at 65 °C. After 25 h, no starting material **20** can be detected anymore, therefore the reaction mixture is cooled to room temperature and subsequently quenched with a saturated aqueous solution of ammonium chloride (1.5 mL). The aqueous phase is extracted with DCM (3 × 15 mL) and the combined organic phases are dried over anhydrous magnesium sulphate. Evaporation of the solvents under reduced pressure and purification of the crude compound via flash chromatography (eluent: 20% EtOAc in petroleum ether) gives title compound **21** (26.6 mg, 71%) as a white solid.

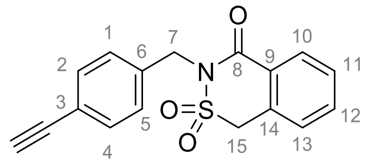

**Formula:** $C_{17}H_{13}NO_3S$
**MW:** 311.355 g/mol
**$R_f$ (20% EtOAc in petroleum ether):** 0.16
**IR:** $\nu_{max}$(cm$^{-1}$): 3288 (w), 2919 (w), 1686 (s), 1348 (s), 1320 (s), 1281 (s), 1163 (s), 1130(m), 1066 (m), 877 (s), 737 (s), 659 (s), 640 (s)
**HRMS (ESI):** calcd for $C_{17}H_{14}NO_3S^+$ [M+H]$^+$: 312.06889; found: 312.0680
**$^1$H NMR (300 MHz, CDCl$_3$):** $\delta$8.22 (dd, *J* = 7.5, 1.5 Hz, 1H, *C10H*), 7.63 (td, *J* = 7.5, 1.5 Hz, 1H, *C12H*), 7.56 (td, *J* = 7.5, 1.5 Hz, 1H, *C11H*), 7.46 (s, 4H, *C1H*, *C2H*, *C4H* and *C5H*), 7.32 (d(br), *J* = 7.5 Hz, 1H, *C13H*), 5.11 (s, 2H, *C7H$_2$*), 4.63 (s, 2H, *C15H$_2$*), 3.06 (s, 1H, C3-C≡*CH*)
**$^{13}$C NMR (75 MHz, CDCl$_3$):** $\delta$163.7 (C), 136.8 (C), 134.1 (CH), 132.3 (CH), 130.9 (CH), 129.7 (CH), 129.1 (CH), 128.8 (CH), 128.1 (C), 127.1 (C), 121.8 (C), 83.3 (C), 77.5 (CH), 54.6 (CH$_2$), 44.2 (CH$_2$)
**HSQC:** 134.1 × 7.63, 132.3 × 7.46, 130.9 × 8.22, 129.7 × 7.56, 129.1 × 7.32, 128.8 × 7.46, 77.5 × 3.06, 54.6 × 4.63, 44.2 × 5.11

## 2.11. 1-bromo-3-(2-methylbenzyl)-1*H*-benzo[*d*][1,2]thiazin-4(3*H*)-one 2,2-dioxide (22)

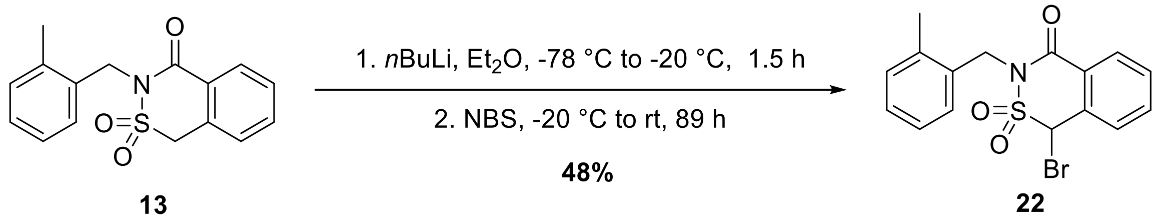


To a solution of starting compound **13** (20.0 mg, 66.3 µmol, 1 equiv.) in $Et_2O$ (1.33 mL), which is cooled to -78 °C, a solution of *n*-BuLi in hexane (2.5 M, 0.035 mL, 86.2 µmmol, 1.3 equiv.) is added dropwise. The mixture is gradually warmed to -20 °C over a period of 1.5 h. After 1.5 h, N-bromosuccinimide (NBS) is added portion wise to the orange coloured mixture at -20 °C. The solution is gradually warmed to room temperature and the conversion rate is regularly checked via TLC (eluent: 30% EtOAc in petroleum ether). The orange colour slowly disappears and the reaction mixture obtains a yellow colour. After 89 h of stirring at room temperature, only a small amount of starting material **21** can still be observed via TLC. Therefore, it is decided to terminate the reaction through the quick addition of a saturated aqueous solution of ammonium chloride (2 mL). The aqueous phase is extracted with DCM (3 × 15 mL) and the combined organic phases are dried over anhydrous magnesium sulphate. Evaporation of the organic solvents under reduced pressure and purification of the crude mixture via flash chromatography (eluent: 10% EtOAc in petroleum ether) gives title compound **22** (12.3 mg, 48%) as a white solid.

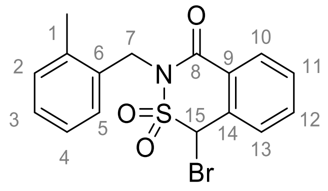

**Formula:** $C_{16}H_{14}NO_3BrS$
**MW:** 380.256 g/mol
**$R_f$ (10% EtOAc in petroleum ether):** 0.21
**IR:** $\nu_{max}$(cm$^{-1}$): 2963 (m), 1679 (s), 1360 (s), 1314 (s), 1286 (s), 1194 (s), 1131 (s), 1064(s), 963 (m), 738 (s), 704 (s), 687 (s)
**HRMS (ESI):** calcd for $C_{16}H_{15}BrNO_3S^+$ [M+H]$^+$: 379.99505; found: 379.9941
**$^1$H NMR (300 MHz, $CDCl_3$):** $\delta$8.24 (dd, *J* = 7.5, 1.5 Hz, 1H, *C10H*), 7.67 (td, *J* = 7.5, 1.5 Hz, 1H, *C12H*), 7.64 (td, *J* = 7.5, 1.5 Hz, *C11H*), 7.50 (dd, *J* = 7.5, 1.5 Hz, 1H, *C13H*), 7.45 - 7.39 (m, 1H, *C3H* or *C5H*), 7.21 - 7.16 (band, 3H, *C2H*, *C4H*, *C3H* or *C5H*), 6.15 (s, 1H, *C15H*-Br), 5.30 (d(AB), *J* = 16.0 Hz, 1H, *C7*H*H*), 5.11 (d(AB), *J* = 16.0 Hz, 1H, *C7H*H), 2.47 (s, 3H, C1-*CH3*)
**$^{13}$C NMR (75 MHz, $CDCl_3$):** $\delta$162.9 (C), 135.5 (C), 134.4 (CH), 133.5 (C), 132.7 (C), 131.6 (CH), 131.2 (CH), 130.4 (CH), 129.1 (CH), 127.7 (CH), 127.2 (CH), 126.2 (CH), 126.1 (C), 54.3 (CH), 44.4 ($CH_2$), 19.3 ($CH_3$)
**HSQC:** 134.4 × 7.67, 131.6 × 8.24, 131.2 × 7.64, 130.4 × 7.21 - 7.16, 129.1 × 7.50, 127.7 × 7.21 - 7.16, 127.2 × 7.45 - 7.39, 126.2 × 7.21 - 7.16, 54.3 × 6.15, 44.4 × 5.30, 44.4 × 5.11, 19.3 × 2.47

## 2.12. 1,3-bis(2-methylbenzyl)-1*H*-benzo[*d*][1,2]thiazin-4(3*H*)-one 2,2-dioxide (23)

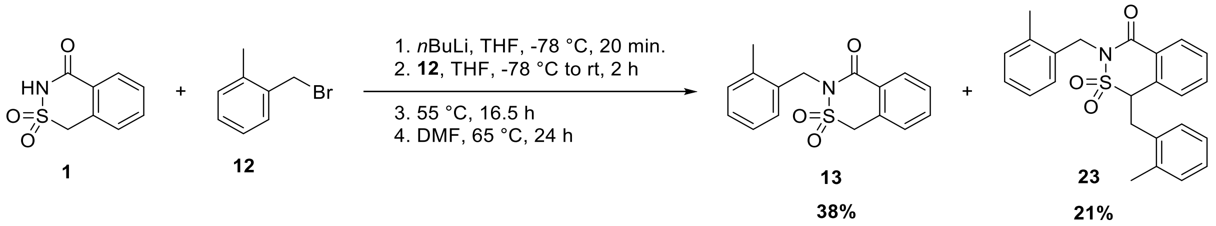

A solution of lactam **1** (200.0 mg, 1.01 mmol, 1 equiv.) in THF (5 mL) is cooled to -78 °C and a solution *n*-BuLi in hexane (2.5 M, 0.5 mL, 1.26 mmol, 1.25 equiv.) is added dropwise to this solution. This mixture is stirred for 20 minutes at -78 °C. Then, a solution of 1-(bromomethyl)-2-methylbenzene **12** (187 mg, 0.10 mmol, 1 equiv.) in THF (5 mL) is slowly added to the lithiated amide solution. The resulting solution is allowed to warm to room temperature over a period of 2 h followed by heating the solution to 55 °C for a period of 16.5 h. After 16.5 h, the conversion rate is checked via TLC analysis (30% EtOAc in petroleum ether). As no conversion can be observed, DMF (2 mL) is added to push the conversion of the reaction. The reaction mixture changes from a milky, turbid solution to a clear, transparent solution. The resulting mixture is stirred for another 24 h at 65 °C. After 24 h, a complete conversion can be deduced from TLC analysis, therefore the reaction mixture is cooled to room temperature and quenched with a saturated aqueous solution of ammonium chloride (12 mL). The aqueous phase is extracted with DCM (3 × 50 mL). The combined organic phases are washed with brine (2 × 20 mL) and dried over anhydrous magnesium sulphate. The organic solvents are removed under reduced pressure and the crude mixture is purified via gradient flash chromatography (eluent: 15% to 20% EtOAc in petroleum ether). The dibenzylated title compound **23** (87 mg, 21%) and single benzylated compound **13** (117 mg, 38%) are isolated as readily separable fast and slow eluting fractions.

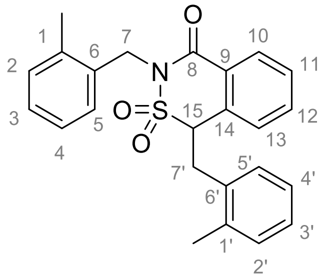

**Formula:** $C_{24}H_{23}NO_3S$
**MW:** 405.512 g/mol
**R$_f$ (30 % EtOAc in petroleum ether):** 0.63
**IR:** $\nu_{max}$(cm$^{-1}$) 2946 (w), 1673 (s), 1353 (s), 1301 (s), 1147 (s), 1047 (m), 752 (s), 695(s)
**HRMS (ESI):** calcd for $C_{24}H_{24}NO_3S^+$ $[M+H]^+$: 406.14714; found: 406.1472
**$^1$H NMR (300 MHz, CDCl$_3$):** $\delta$8.23 (dd, *J* = 7.5, 1.5 Hz, 1H, *C10H*), 7.49 (td, *J* = 7.5, 1.5 Hz, 1H, *C11H*), 7.45 - 7.39 (m, 1H, *C5H*), 7.32 (td, *J* = 7.5, 1.5 Hz, 1H, *C12H*), 7.21 - 7.16 (band, 1H, *C2H*), 7.19 - 7.14 (band, 3H, *C3H*, *C4H* and *C3'H*), 7.11 (td, *J* = 7.5, 1.5 Hz, 1H, *C2'H*), 7.05 (td, *J* = 7.5, 1.5 Hz, 1H, *C4'H*), 6.72 (d(br), *J* = 7.5 Hz, 1H, *C5'H*), 6.43 (d(br), *J* = 7.5 Hz, 1H, *C13H*), 5.24 (d(AB), *J* = 15.8 Hz, 1H, *C7H*H), 5.16 (d(AB), *J* = 15.8 Hz, 1H, *C7*H*H*), 4.47 (dd, *J* = 11.3, 3.4 Hz, 1H, *C15H*), 3.75 (dd, *J* = 13.4, 3.4 Hz, 1H, *C7'H*H), 2.89 (dd, *J* = 13.4, 11.3 Hz, 1H, *C7'*H*H*), 2.51 (s, 3H, *C1H-CH$_3$*), 1.98 (s, 3H, *C1'H-CH$_3$*)
**$^{13}$C NMR (75 MHz, CDCl$_3$):** $\delta$163.9 (C), 136.6 (C), 135.7 (C), 134.1 (C), 133.1 (CH), 132.74 (C), 132.67 (C), 131.0 (CH), 130.7 (CH), 130.5 (CH), 130.4 (CH), 129.9 (CH), 129.4(CH), 127.7 (CH), 127.6 (CH), 127.3 (CH), 126.2 (CH), 126.2 (C), 64.3 (CH), 42.5 (CH$_2$), 36.3 (CH$_2$), 19.4 (CH$_3$), 19.0 (CH$_3$)
**HSQC:** 133.1 × 7.32, 131.0 × 8.23, 130.7 × 7.11, 130.5 × 6.72, 130.4 × 7.21 - 7.16, 129.9 × 6.43, 129.4 × 7.49, 127.7 × 7.19 - 7.14, 127.6 × 7.19 - 7.14, 127.3 × 7.45 - 7.39, 126.2 × 7.19 - 7.14, 126.2 × 7.05, 64.3 × 4.47, 42.5 × 5.24, 42.5 × 5.16, 36.3 × 3.75, 36.3 × 2.89, 19.4 × 2.51, 19.0 × 1.98

## 2.13. 3-(2-methylbenzyl)-1-(prop-2-yn-1-yl)-1*H*-benzo[*d*][1,2]thiazin-4(3*H*)-one 2,2-dioxide (25)

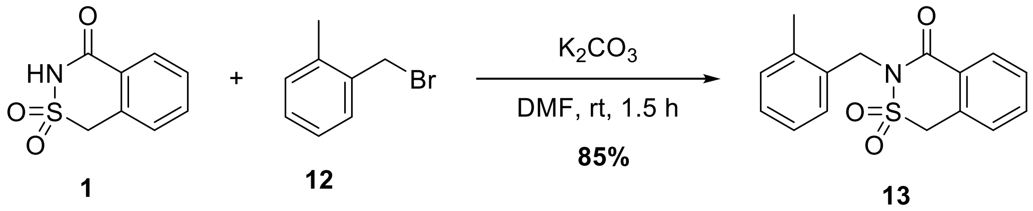


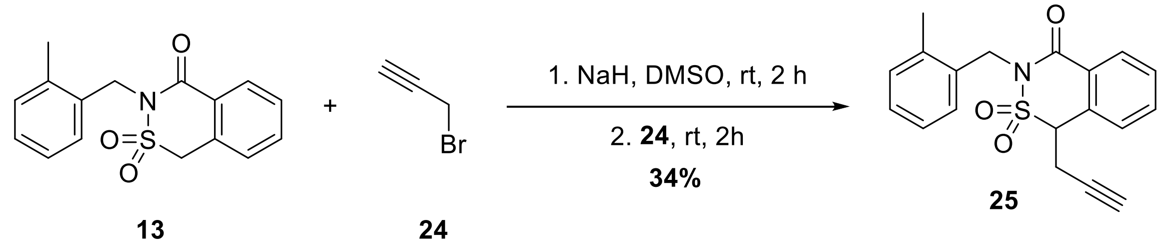


Lactam **1** (190.0 mg, 0.96 mmol, 1 equiv.) was dissolved in DMF (8 ml) in a round bottom flask, followed by the addition of $K_2CO_3$ (201.8 mg, 1.45 mmol, 1.5 equiv.). Then 1-(bromomethyl)-2-methylbenzene **12** (0.20 ml, 1.45 mmol, 1.5 equiv.) was added, after which the reaction mixture was stirred at room temperature for 1.5 h. The reaction was quenched with a saturated $NaHCO_3$ solution (15 ml). This mixture was extracted with EtOAc (1 × 150 ml). This organic phase was then washed with $H_2O$ (5 × 12 ml) after which it was dried on sodium sulphate. The organic solvents are removed under reduced pressure and the crude mixture is purified via flash chromatography (eluent: 10% EtOAc in pentane) giving 248.1 mg of the benzylated lactam **13** as a white powder (85% yield).

This benzylated lactam **13** (90.4 mg, 0.3 mmol, 1 equiv.) was then added to a roundbottom flask containing a solution of NaH (60% dispersion, 11.4 mg, 0.285 mmol, 0.95 equiv.) in dmso (6 ml). This orange mixture was then stirred for 2 h at room temperature after which the reaction flask was covered with aluminium foil before adding propargyl bromide (**24**) to this mixture. This was then stirred for 21 h at room temperature after which it was quenched with $H_2O$ (10 ml) and extracted with EtOAc (1 × 100 ml). This organic phase was then washed with $H_2O$ (5 x 15 ml) and dried on sodium sulphate after which the organic solvents are removed under reduced pressure and the crude mixture is purified via flash chromatography (eluent: 5% EtOAc in pentane). This yielded 34.9 mg of the title compound **25** as a white solid (34% yield).

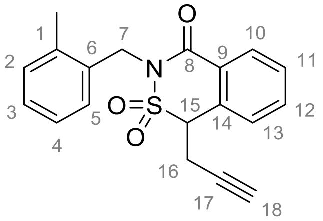

**Formula:** $C_{19}H_{17}NO_3S$
**MW:** 339.409 g/mol
**$R_f$ (30% EtOAc in Pentane):** 0.50
**HRMS (ESI):** calcd for $C_{19}H_{18}NO_3S^+$ $[M+H]^+$: 340.10019; found: 340.1000
**$^1$H NMR (700 MHz, CDCl$_3$):** $\delta$8.23 (dd, *J* = 7.6, 1.4 Hz, 1H, *C10H*), 7.66 (td(AB), *J* = 7.6, 1.4 Hz, 1H, *C12H*), 7.59 (td(AB), *J* = 7.6, 1.4 Hz, 1H, *C11H*), 7.46 (dd, *J* = 7.6, 1.4 Hz, 1H, *C13H*), 7.38 - 7.33 (m, 1H, *C5H*), 7.19 - 7.16 (band, 3H, *C2H*, *C3H* and *C4H*), 5.18 (d(AB), *J* = 15.9 Hz, 1H, *C7H*H), 5.05 (d(AB), *J* = 15.9 Hz,

1H, *C7*H*H*), 4.54 (dd, *J* = 8.3, 4.3 Hz, 1H, *C15H*), 3.17 (ddd(AB), *J* = 17.0, 4.3, 2.7 Hz, 1H, *C16H*H), 2.88 (ddd(AB), *J* = 17.0, 8.3, 2.7 Hz, 1H, *C16*H*H*), 2.47 (s, 3H, C1-*CH₃*), 2.11 (t, *J* = 2.7 Hz, 1H, *C18H*)
**$^{13}$C NMR (175 MHz, CDCl$_3$):** $\delta$163.7 (C), 135.6 (C), 133.9 (C), 133.7 (CH), 131.8 (C), 130.9 (CH), 130.4 (CH), 129.9 (CH), 129.7 (CH), 127.6 (CH), 127.3 (CH), 126.7 (C), 126.2 (CH), 76.6 (C), 73.7 (CH), 61.2 (CH), 43.0 (CH2), 23.2 (CH2), 19.4 (CH3)
**HSQC:** 133.7 × 7.66, 130.9 × 8.23, 130.4 × 7.19 - 7.16, 129.9 ×, 129.7 ×, 127.6 × 7.19 -7.16, 127.3 × 7.38 - 7.33, 126.2 × 7.19 - 7.16, 73.7 × 2.11, 61.2 × 4.54, 43.0 × 5.18, 43.0 × 5.05, 23.2 × 3.17, 23.2 × 2.88, 19.4 × 2.47

## 2.14. *N*-(2-(2-(2-(2-(4-((3-(2-methylbenzyl)-2,2-dioxido-4-oxo-3,4-dihydro-1*H*-benzo[*d*][1,2]thiazin-1-yl)methyl)-1*H*-1,2,3-triazol-1-yl)ethoxy)ethoxy)ethoxy)ethyl)-5-((3a*S*,4*S*,6a*R*)-2-oxohexahydro-1*H*-thieno[3,4-*d*]imidazol-4-yl)pentanamide (27)

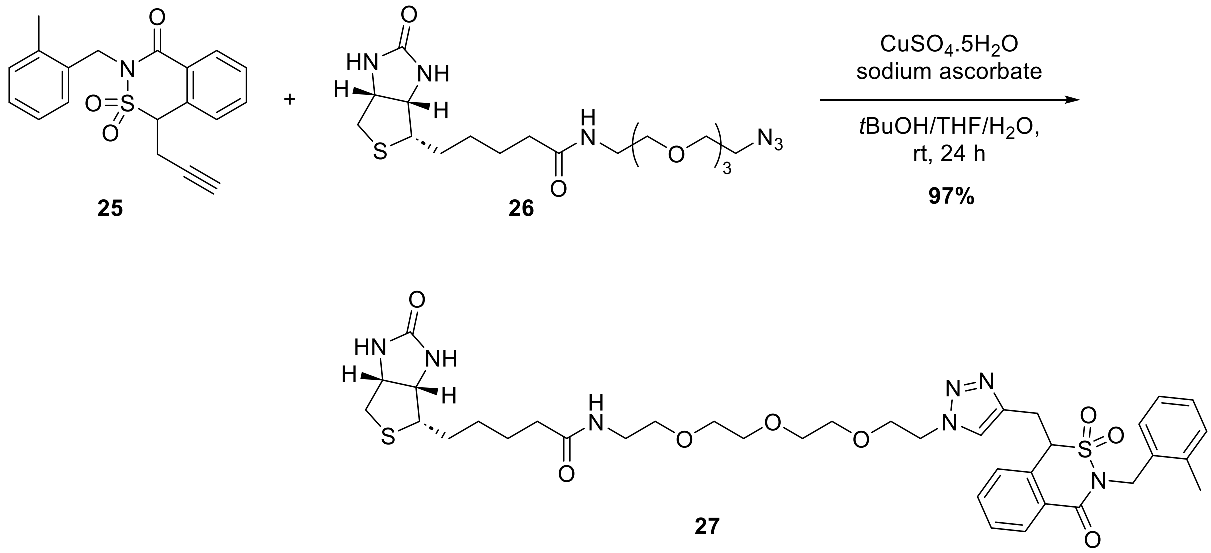


Alkyne **25** (36.2 mg, 0.11 mmol, 1 equiv.) and biotin-PEG3-azide **26** (44.7 mg, 0.10 mmol, 0.94 equiv.) were brought into a screwcap vial onto which a mixture of THF (0.18 ml), *t*BuOH (0.54 ml) and $H_2O$ (0.36 ml) were added. Then a 0.5 M stock solution of $CuSO_4$ and sodium ascorbate in $H_2O$ was added (0.05 ml). The reaction was then stirred at room temperature for 24 hours after which the mixture was evaporated and purified via prep-HPLC. This yielded 81 mg of the clicked product **27** as a white solid (97 % yield).

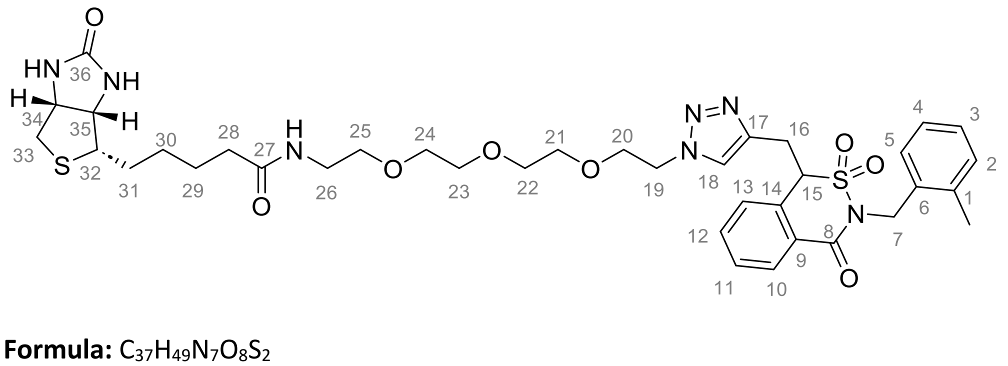

**Formula:** $C_{37}H_{49}N_7O_8S_2$
**MW:** 783.96 g/mol
**R$_f$ (10% MeOH in DCM):** 0.39
**MS (ESI):** calcd for $C_{37}H_{49}N_7O_8S_2^+$ [M+H]$^+$: 784.3; found: 784.2

**$^{1}$H NMR (500 MHz, MeOD-d$_4$):** $\delta$8.12 (dd, *J* = 7.3, 2.0 Hz, 1H, *C10H*), 7.61 (td, *J* = 7.5, 1.7 Hz, 1H, *C12H*), 7.58 (td, *J* = 7.5, 1.6 Hz, 1H, *C11H*), 7.52 (s, 1H, *C18H*), 7.31 (m, 1H, *C3H*), 7.21-7.10 (band, 4H, *C2H, C4H, C5H and C13H*), 5.20 (dd, *J* = 8.2, 5.0 Hz, 1H, *C15H*), 5.09 (d(AB), *J* = 16.2 Hz, 1H, *C7H*H), 5.01 (d(AB), *J* = 16.2 Hz, 1H, *C7*H*H*), 4.49 (m, 2H, *C19H$_2$*), 4.46 (dd, *J* = 7.9, 5.0 Hz, 1H, *C35H*), 4.27 (dd, *J* = 7.9, 4.4 Hz, 1H, *C34H*), 3.81 (t, *J* = 5.0 Hz, 2H, *C20H$_2$*), 3.64 (dd, *J* = 14.9, 5.0 Hz, 1H, *C16H*H), 3.56 (band, 8H, *C21H$_2$, C22H$_2$*, C23H$_2$, C24H$_2$), 3.50 (t, *J* = 5.5 Hz, 2H, *C25H$_2$*), 3.36 (dd, *J* = 14.9, 8.2 Hz, 1H, *C16H*), 3.33 (m, 2H, *C26H$_2$*), 3.17 (m, 1H, *C32H*), 2.90 (dd, *J* = 12.7, 5.0 Hz, 1H, *C33H*H), 2.68 (d, *J* = 12.7 Hz, 1H, *C33*H*H*), 2.45 (s, 3H, C1-*CH$_3$*), 2.18 (t, *J* = 7.4 Hz, 2H, *C28H$_2$*), 1.76-1.50 (band, 4H, *C31H$_2$ and C29H$_2$*), 1.41 (m, 2H, *C30H$_2$*),
**$^{13}$C NMR (100 MHz, MeOD-d$_4$):** $\delta$176.1 (C=O, *C27*), 166.1 (C=O, *C36*), 165.1 (C=O, *C8*), 142.0 (Cq, *C17*), 136.8 (Cq, *C1*), 135.8 (Cq, *C6*), 135.0 (Ar-CH, *C11*), 134.5 (Ar-Cq, *C14*), 131.4 (Ar-CH, *C10*), 131.3 (Ar-CH, *C13*), 131.1 (Ar-CH, *C2*), 130.7 (Ar-CH, *C12*), 128.4 (Ar-CH, *C5*), 128.2 (Ar-CH, *C3*), 128.0 (Ar-Cq, *C9*), 127.1 (Ar-CH, *C4*), 125.9 (CH, *C18*), 71.5 (CH$_2$, *C22/C23*), 71.4 (CH$_2$, *C22/C23*), 71.3 (CH$_2$, *C21*), 71.2 (CH$_2$, *C24*), 70.5 (CH$_2$, *C25*), 70.3 (CH$_2$, *C20*), 63.7 (CH, *C15*), 63.4 (CH, *C34*), 61.6 (CH, *C35*), 57.0 (CH, *C32*), 51.4 (CH2, *C19*), 44.1 (CH$_2$, *C7*), 41.0 (CH$_2$, *C33*), 40.3 (CH$_2$, *C26*), 36.7 (CH$_2$, *C28*), 30.0 (CH$_2$, *C16*), 29.7 (CH$_2$, *C30*), 29.5 (CH$_2$, *C31*), 26.8 (CH$_2$, *C29*), 19.5 (CH$_3$, C1-*CH$_3$*)
**HSQC:** 8.12 × 131.4, 7.61 × 130.7, 7.58 × 135.0, 7.52 × 125.9, 7.31 × 128.2, 7.21-7.15 × 131.3 , 7.21-7.15 × 131.1 , 7.19-7.12 × 128.4, 7.16-7.10 × 127.1 , 5.20 × 63.7, 5.09 × 44.1, 5.01 × 44.1, 4.49 × 51.4, 4.46 × 61.6, 4.27 × 63.4, 3.81 × 70.3, 3.64 × 30.0, 3.56 × 71.5, 3.56 × 71.4, 3.56 × 71.3, 3.56 × 71.2, 3.50 × 70.5, 3.36 × 30.0, 3.33 × 40.3, 3.17 × 57.0, 2.90 × 41.0, 2.68 × 41.0, 2.45 × 19.5, 2.18 × 36.7, 1.74-1.66 × 29.5, 1.68-1.57 × 26.8, 1.61-1.53 × 29.5, 1.41 × 29.7

# 3. Copies of the NMR spectra:

## 3.1. 3-((2,2-dioxido-4-oxo-1,4-dihydro-3H-benzo[*d*][1,2]thiazin-3-yl)methyl)benzonitrile (3)

$^{1}$H-NMR (400 MHz, $CDCl_3$)

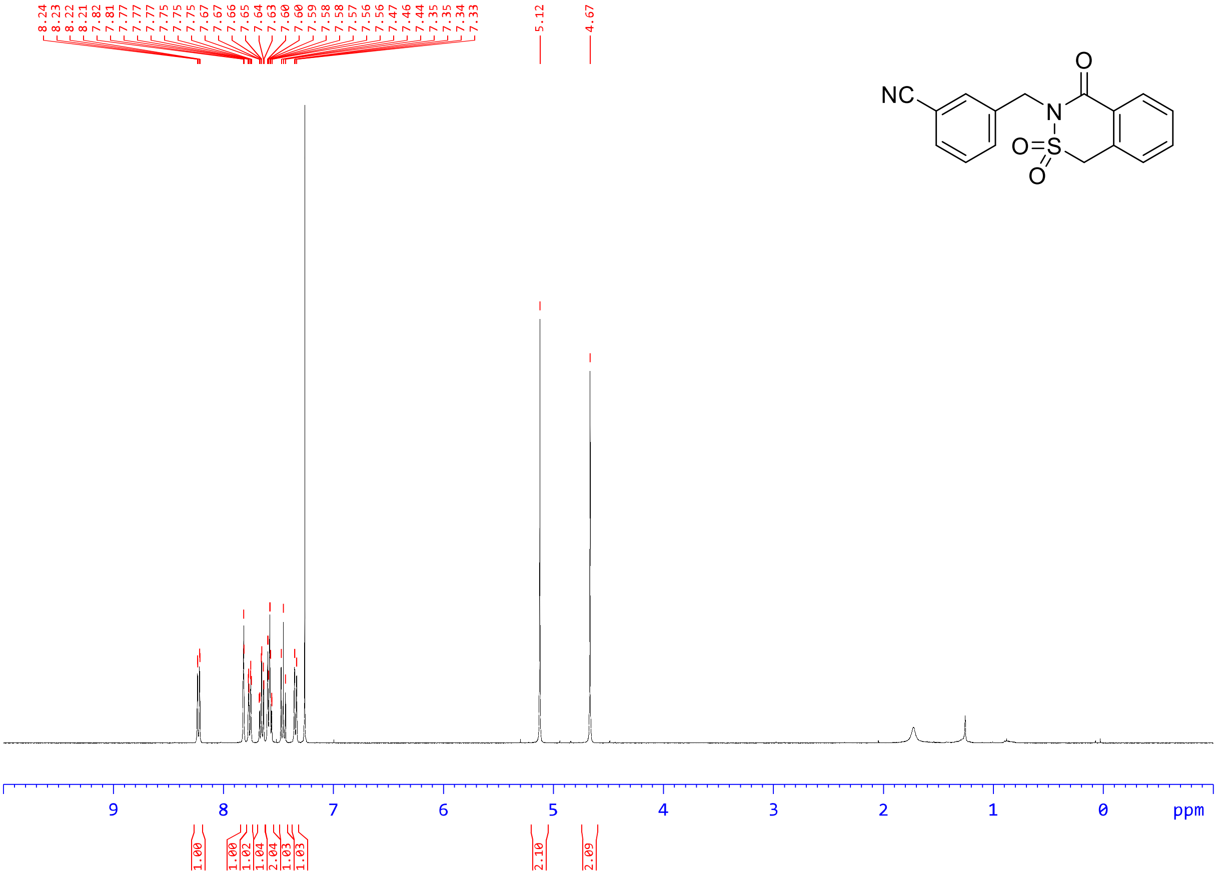


$^{13}C\{^{1}H\}$-NMR (100 MHz, $CDCl_3$)

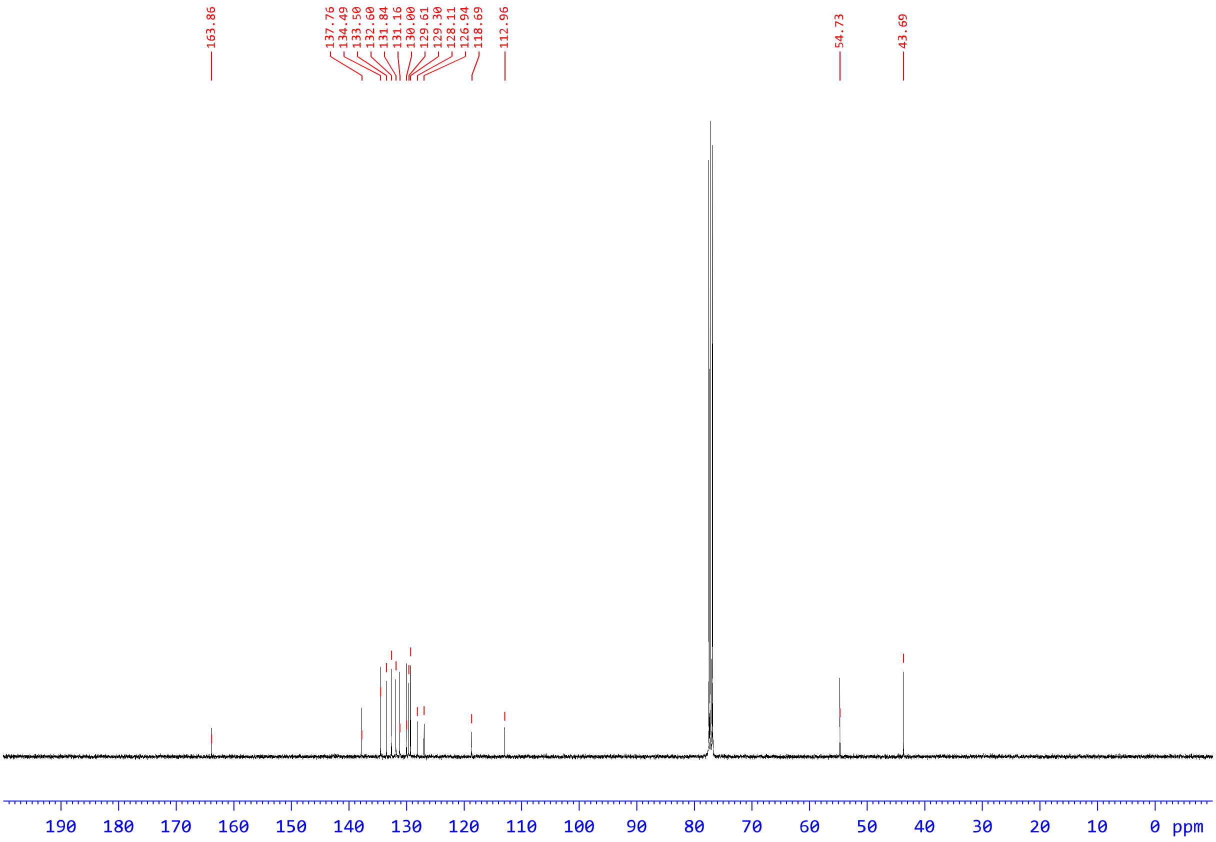

163.86
137.76
134.49
133.50
132.60
131.84
131.16
130.00
129.61
129.30
128.11
126.94
118.69
112.96
54.73
43.69
190 180 170 160 150 140 130 120 110 100 90 80 70 60 50 40 30 20 10 0 ppm

### 3.2. 3-(4-bromobenzyl)-1H-benzo[*d*][1,2]thiazin-4(3*H*)-one 2,2-dioxide (5)

$^{1}$H-NMR (300 MHz, $CDCl_3$)

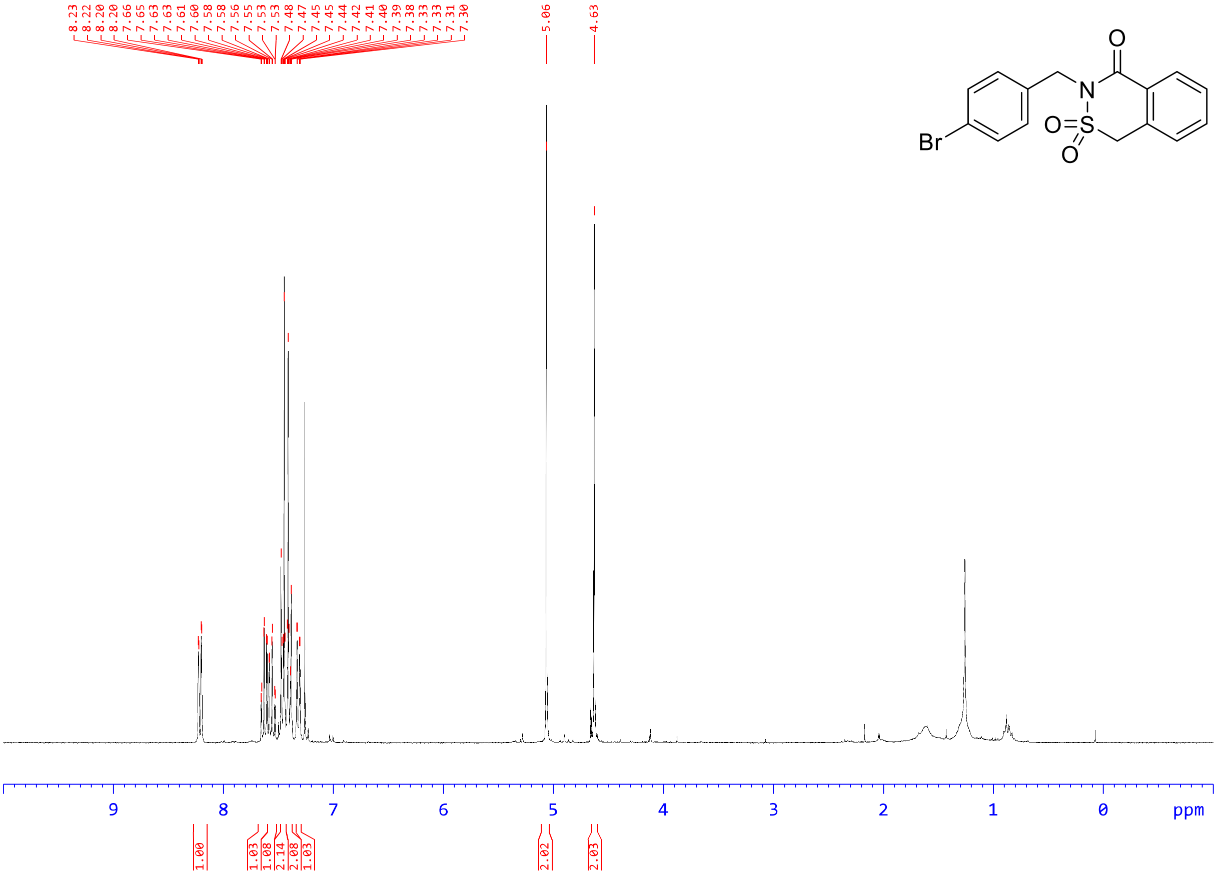


$^{13}$C{$^{1}$H}-NMR (75 MHz, $CDCl_3$)

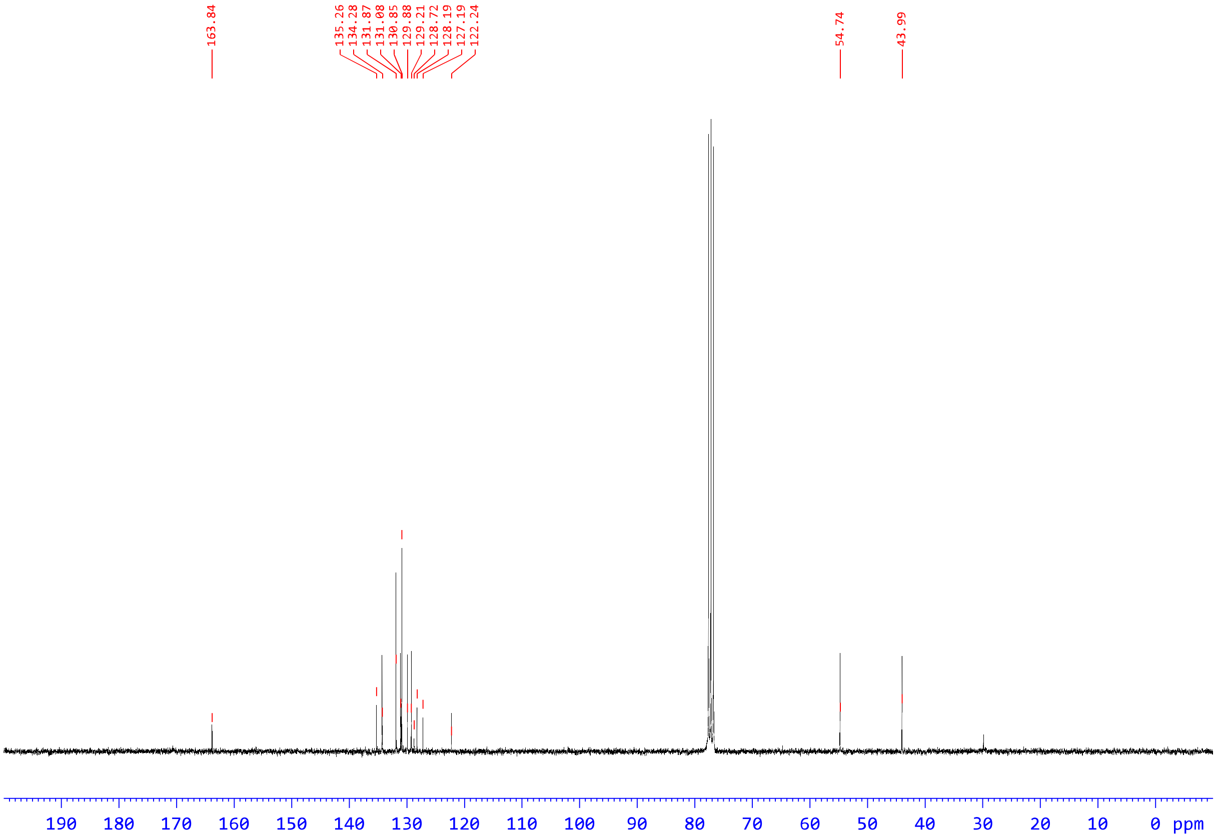

3.3. 3-(3,5-dimethylbenzyl)-1H-benzo[*d*][1,2]thiazin-4(3*H*)-one 2,2-dioxide (7)

$^{1}$H-NMR (400 MHz, $CDCl_3$)

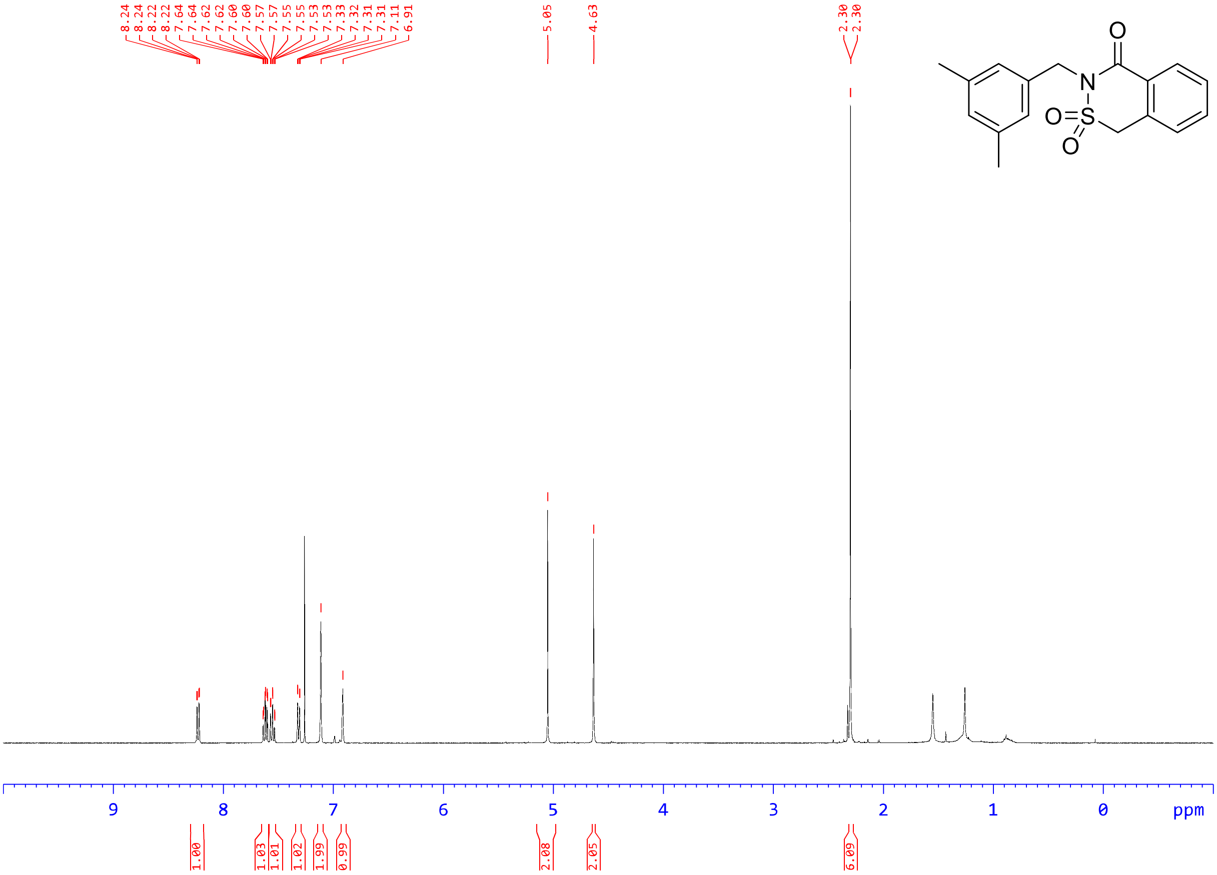


$^{13}C\{^{1}H\}$-NMR (100 MHz, $CDCl_3$)

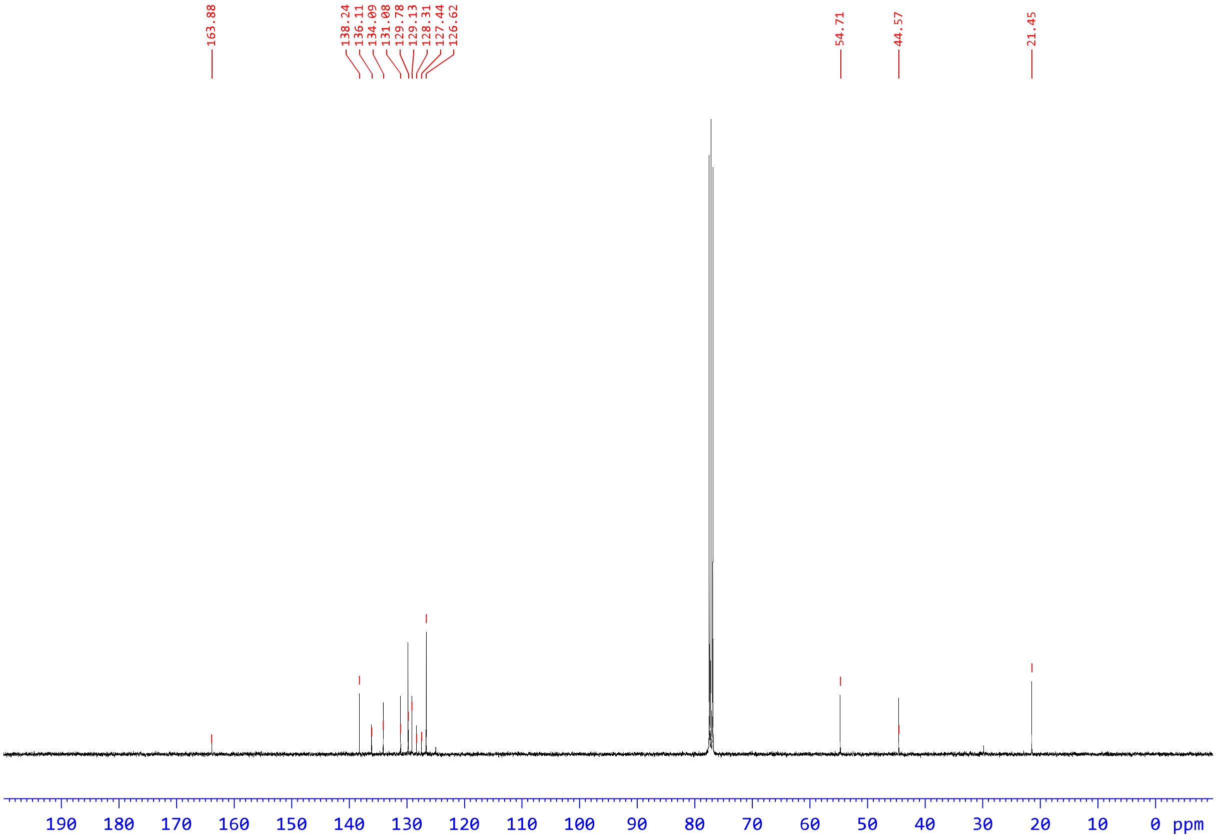

3.4. 3-(3-bromobenzyl)-1H-benzo[*d*][1,2]thiazin-4(3*H*)-one 2,2-dioxide (9)

$^{1}H$-NMR (300 MHz, $CDCl_3$)

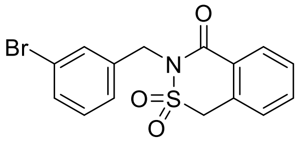

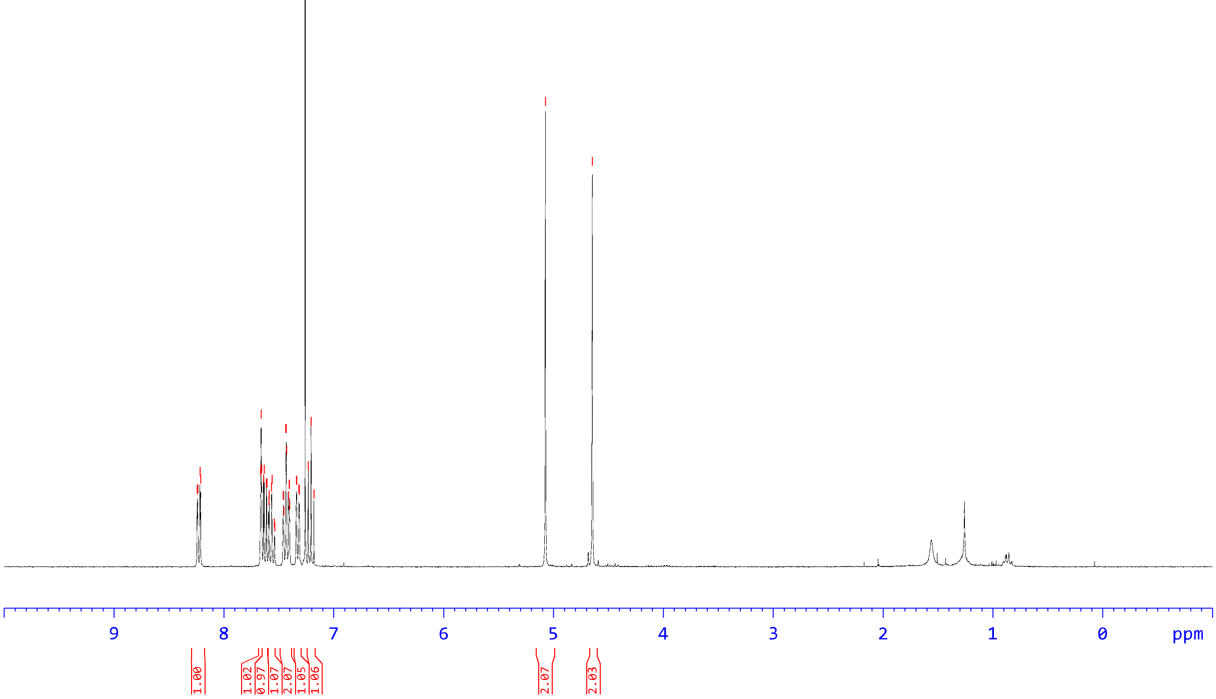

$^{13}C\{^{1}H\}$-NMR (75 MHz, $CDCl_3$)

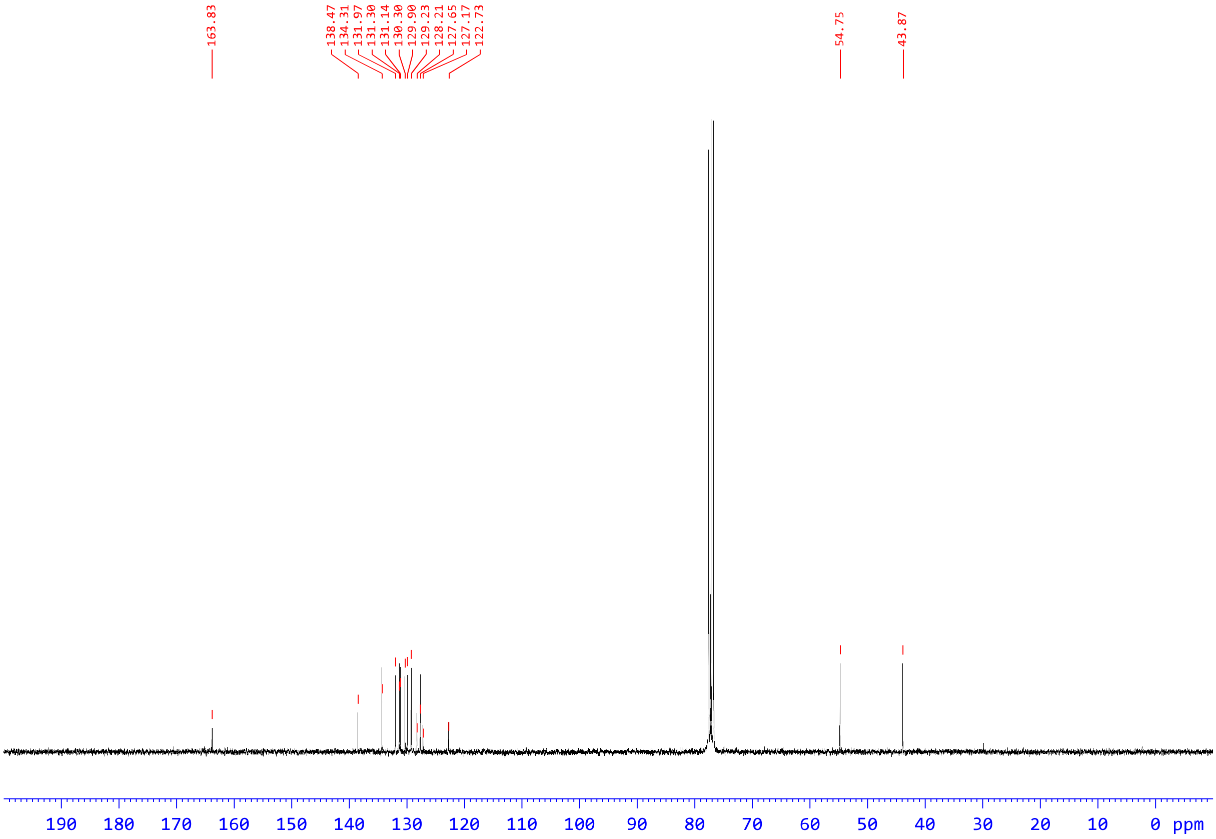

### 3.5. 3-(2-bromobenzyl)-1H-benzo[*d*][1,2]thiazin-4(3*H*)-one 2,2-dioxide (11)

$^1$H-NMR (300 MHz, $CDCl_3$)

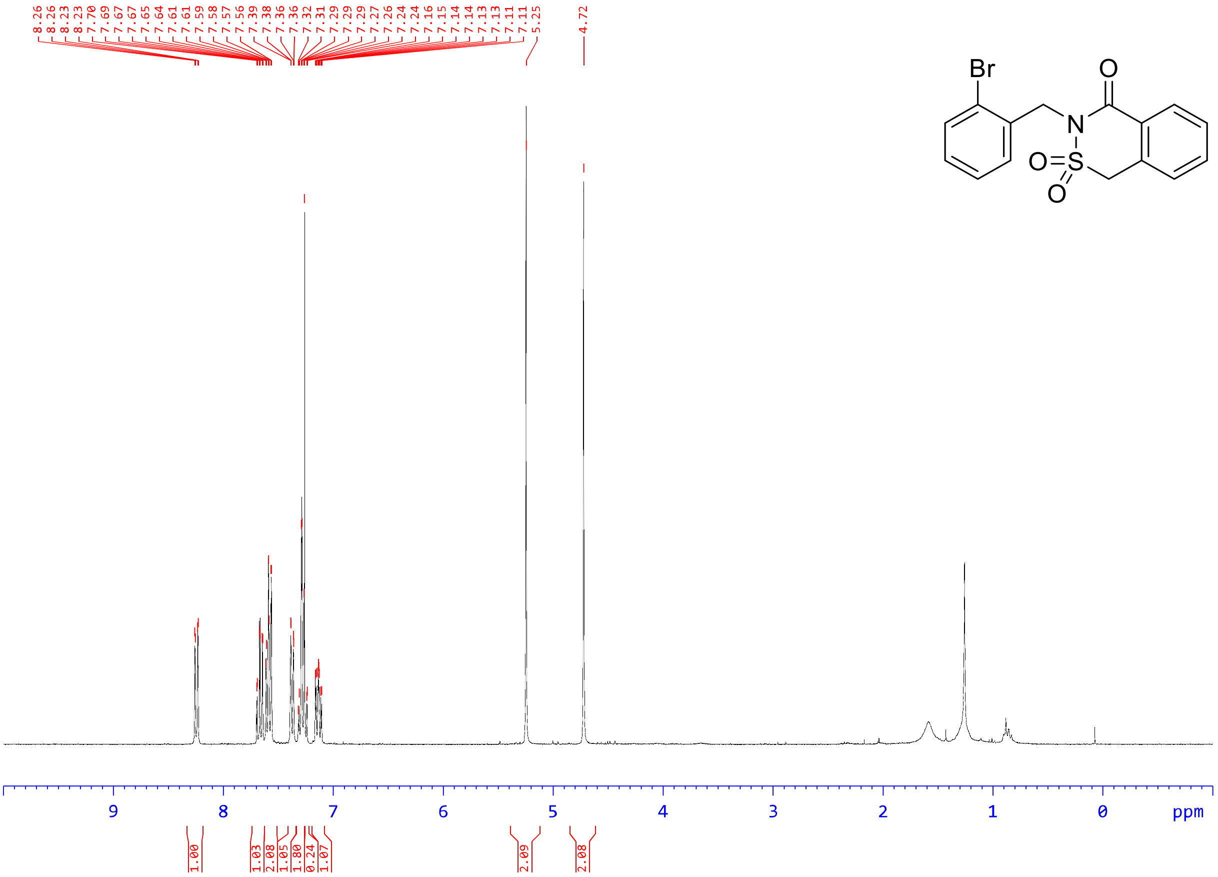


$^{13}$C{$^1$H}-NMR (75 MHz, $CDCl_3$)

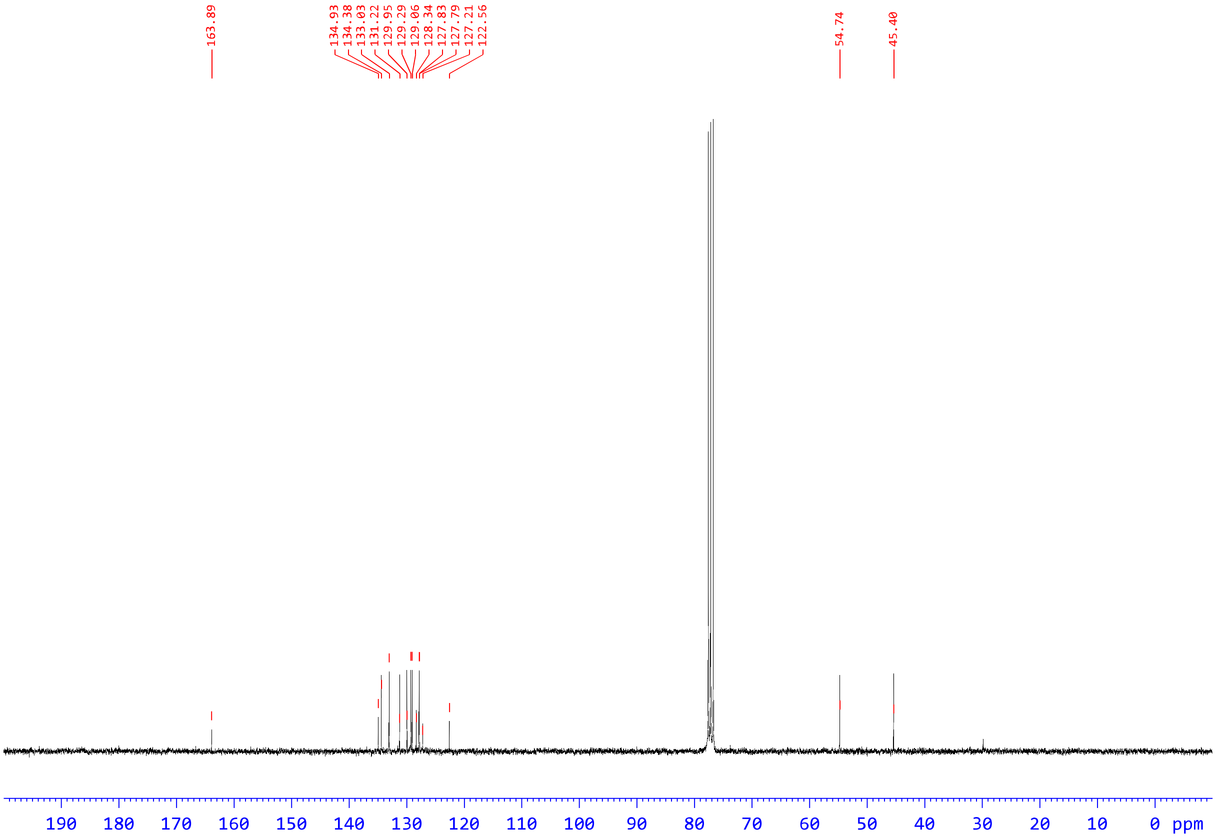

## 3.6. 3-(2-methylbenzyl)-1H-benzo[*d*][1,2]thiazin-4(3*H*)-one 2,2-dioxide (13)

$^{1}$H-NMR (300 MHz, $CDCl_3$)

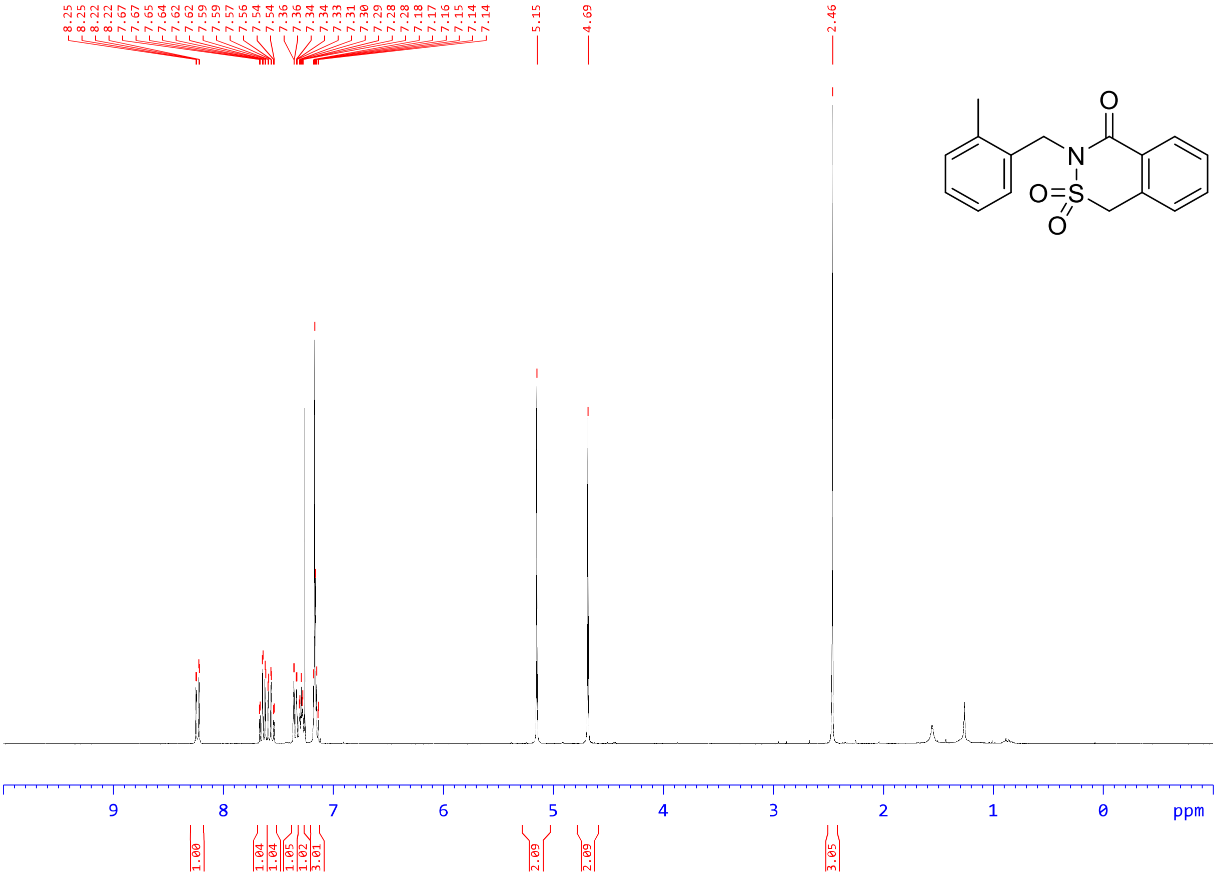


$^{13}$C{$^{1}$H}-NMR (75 MHz, $CDCl_3$)

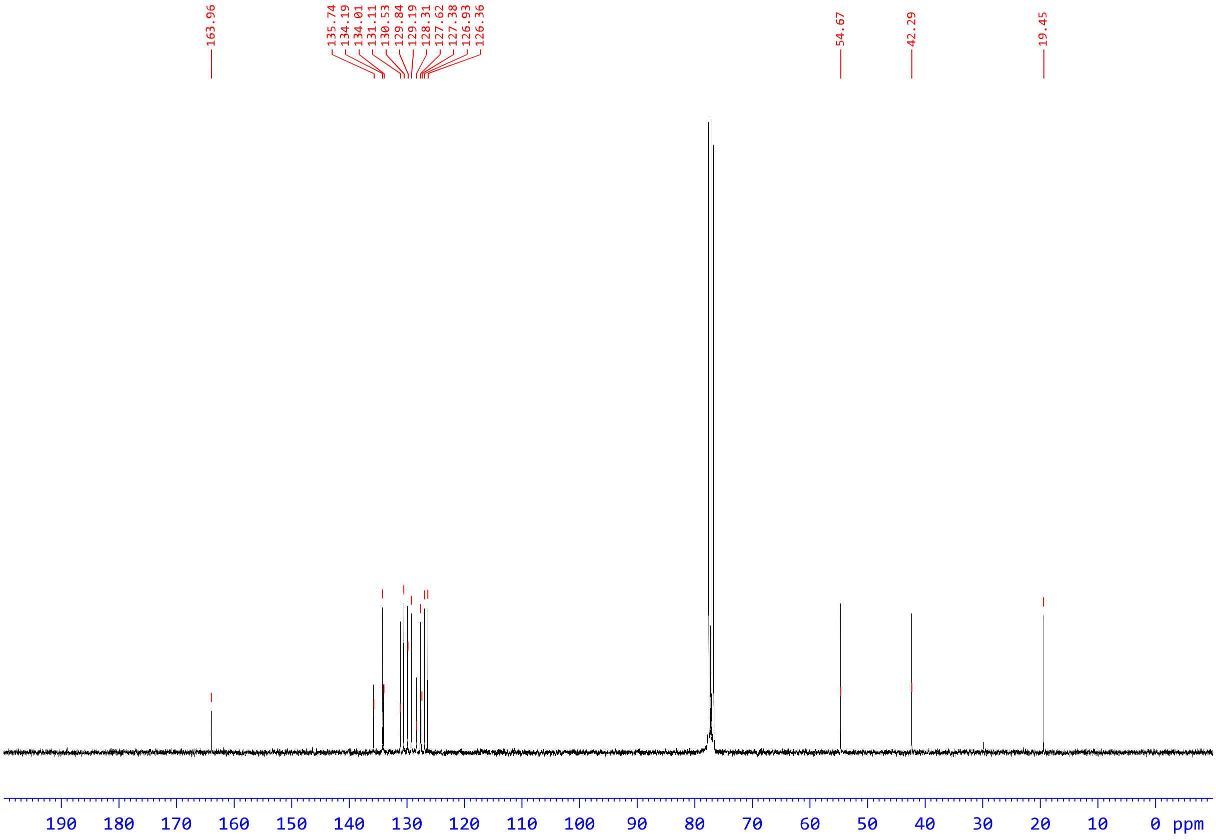

## 3.7. 3-(3-methylbenzyl)-1H-benzo[*d*][1,2]thiazin-4(3*H*)-one 2,2-dioxide (15)

$^{1}$H-NMR (300 MHz, $CDCl_3$)

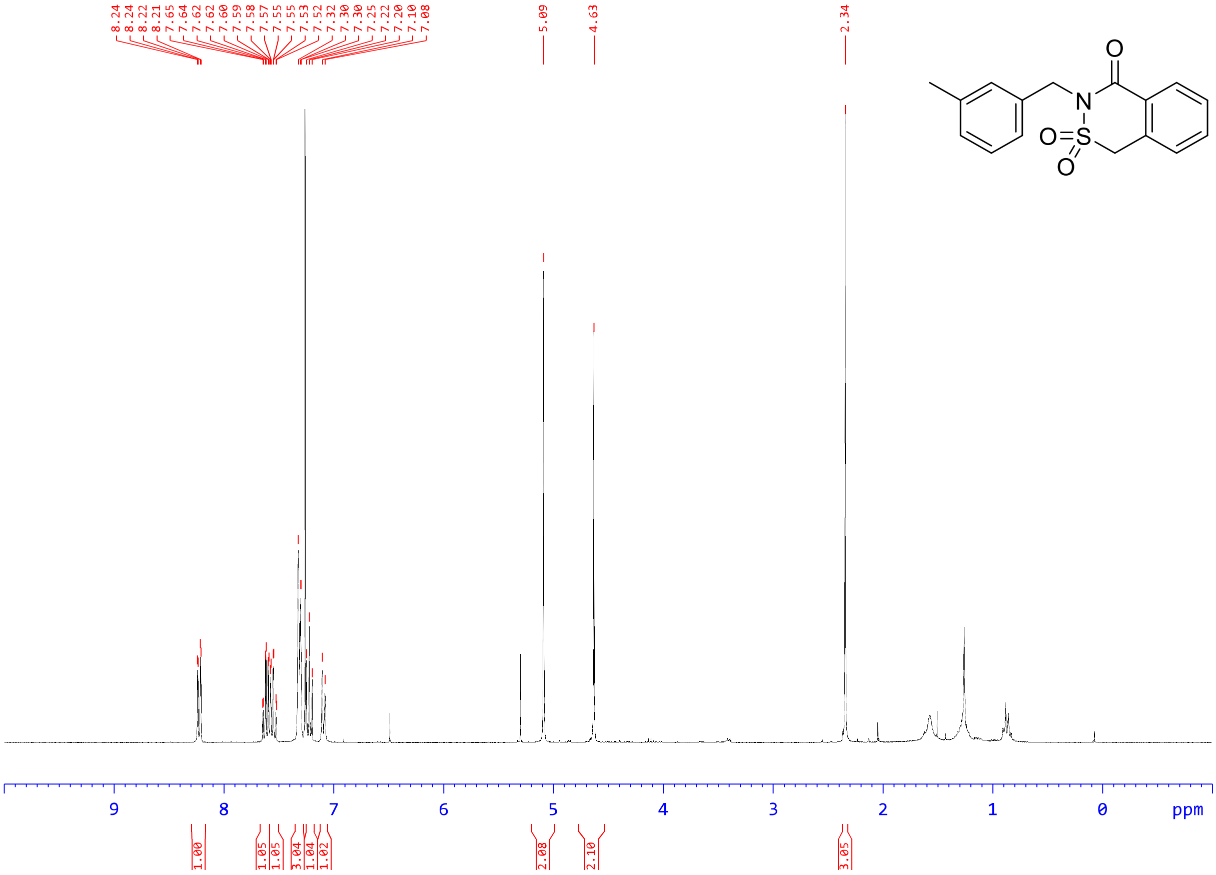


$^{13}$C{$^{1}$H}-NMR (75 MHz, $CDCl_3$)

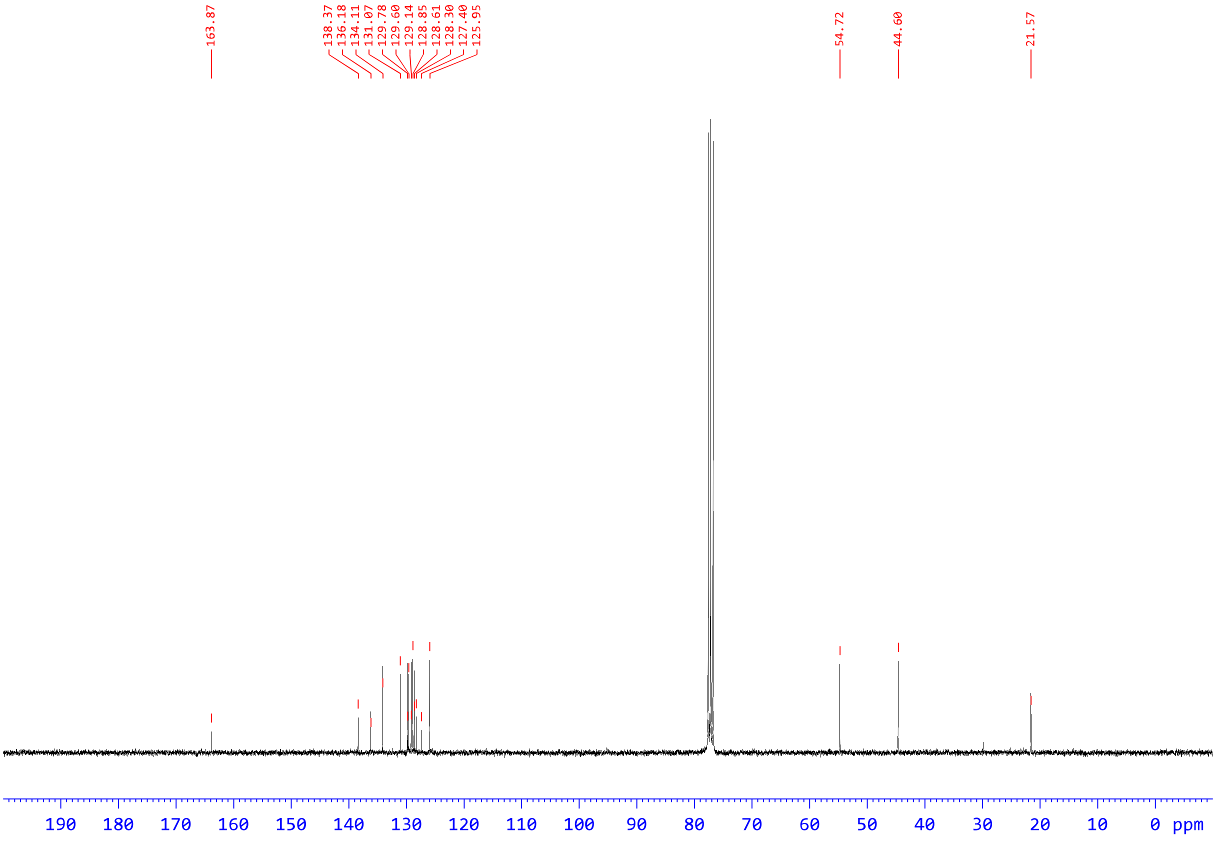

## 3.8. 3-(2-methoxybenzyl)-1H-benzo[*d*][1,2]thiazin-4(3*H*)-one 2,2-dioxide (17)

$^{1}$H-NMR (300 MHz, $CDCl_3$)

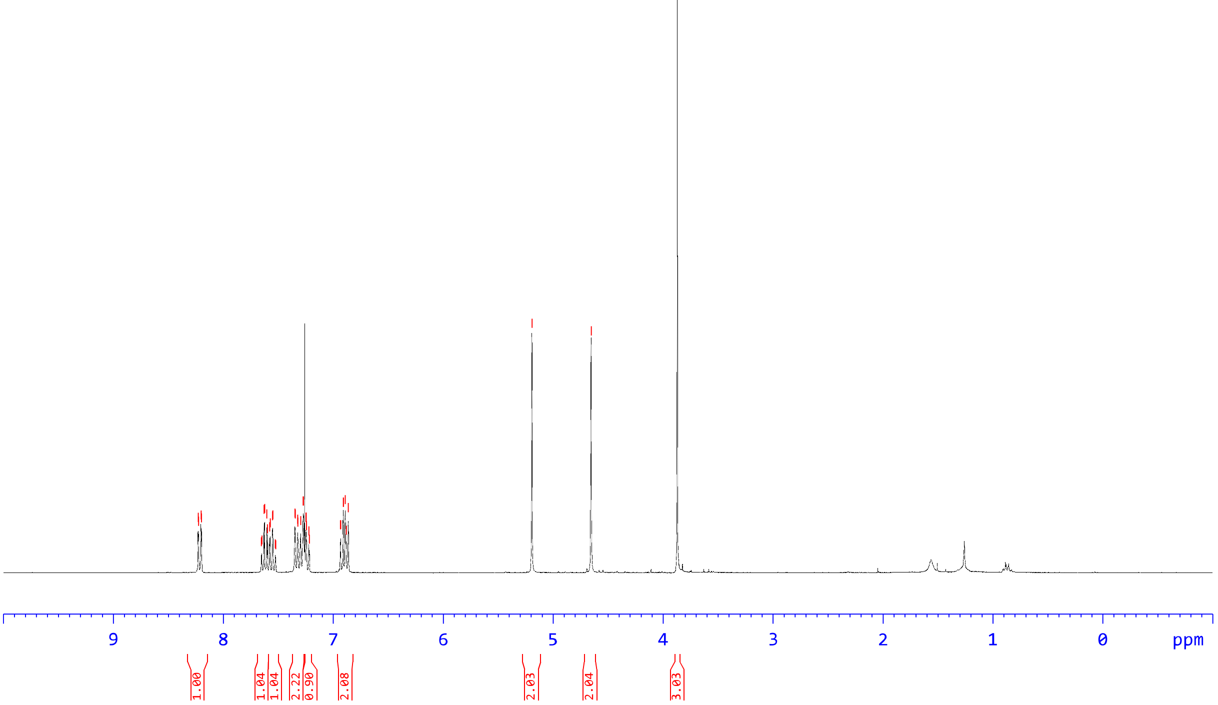


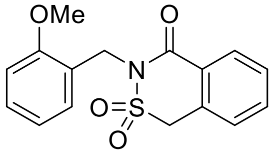


$^{13}$C{$^{1}$H}-NMR (75 MHz, $CDCl_3$)

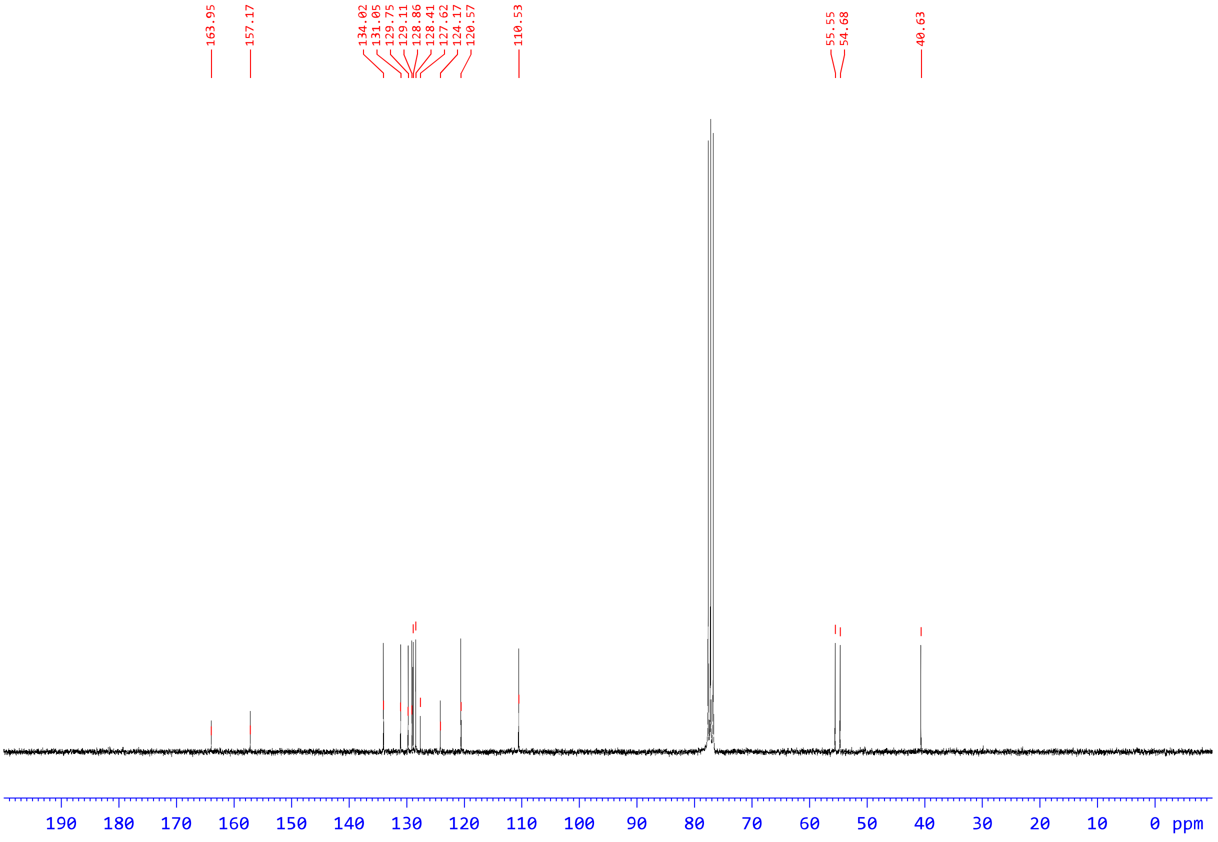

## 3.9. 3-benzyl-1H-benzo[*d*][1,2]thiazin-4(3*H*)-one 2,2-dioxide (19)

$^{1}$H-NMR (300 MHz, $CDCl_3$)

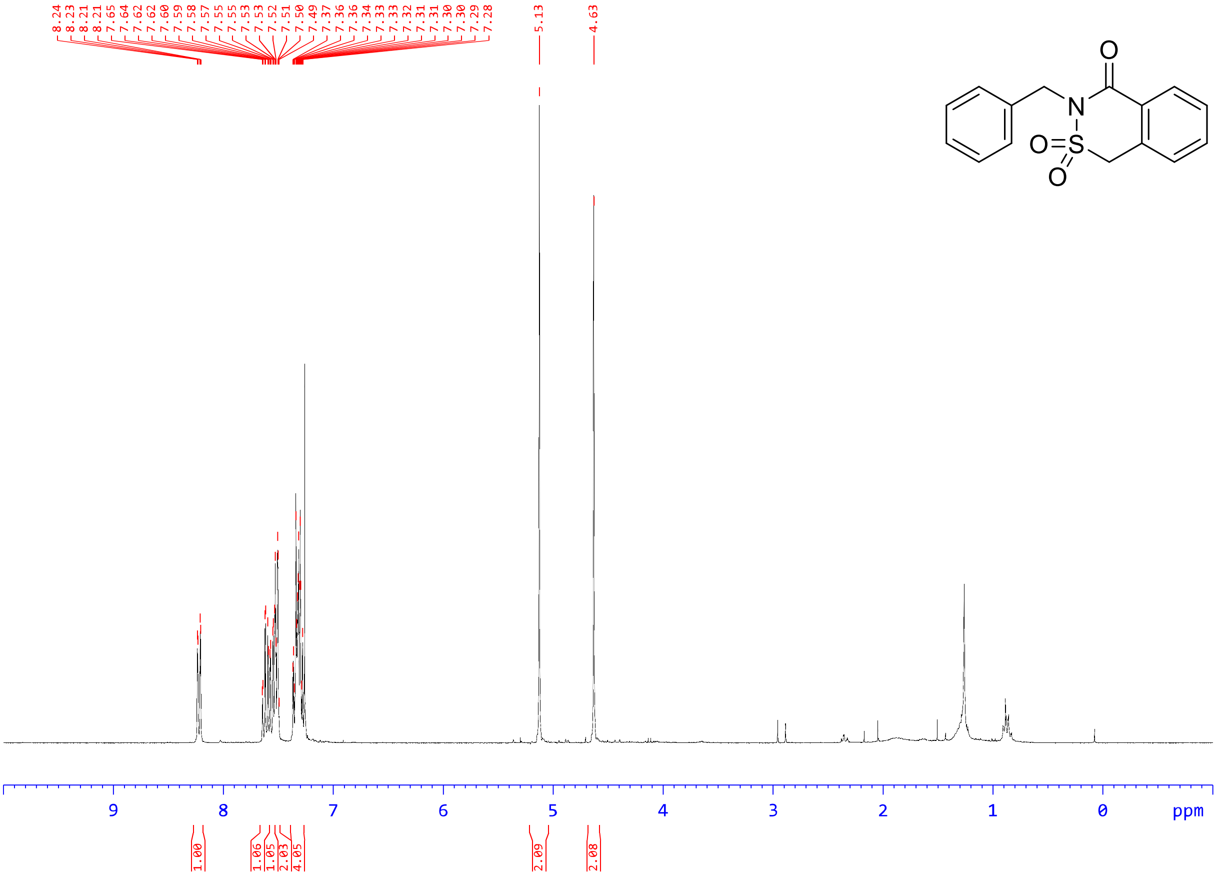


$^{13}$C{$^{1}$H}-NMR (75 MHz, $CDCl_3$)

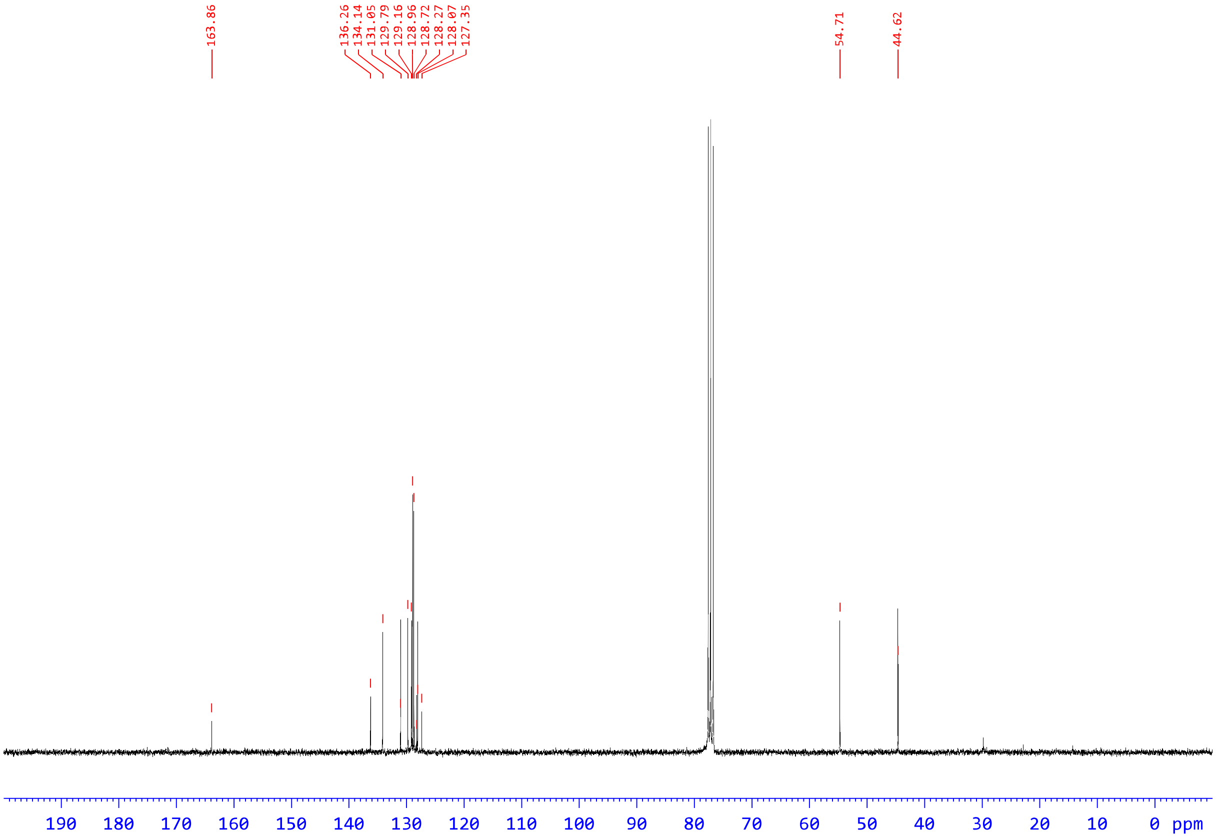

## 3.10. 3-(4-ethynylbenzyl)-1H-benzo[*d*][1,2]thiazin-4(3*H*)-one 2,2-dioxide (21)

$^{1}$H-NMR (300 MHz, $CDCl_3$)

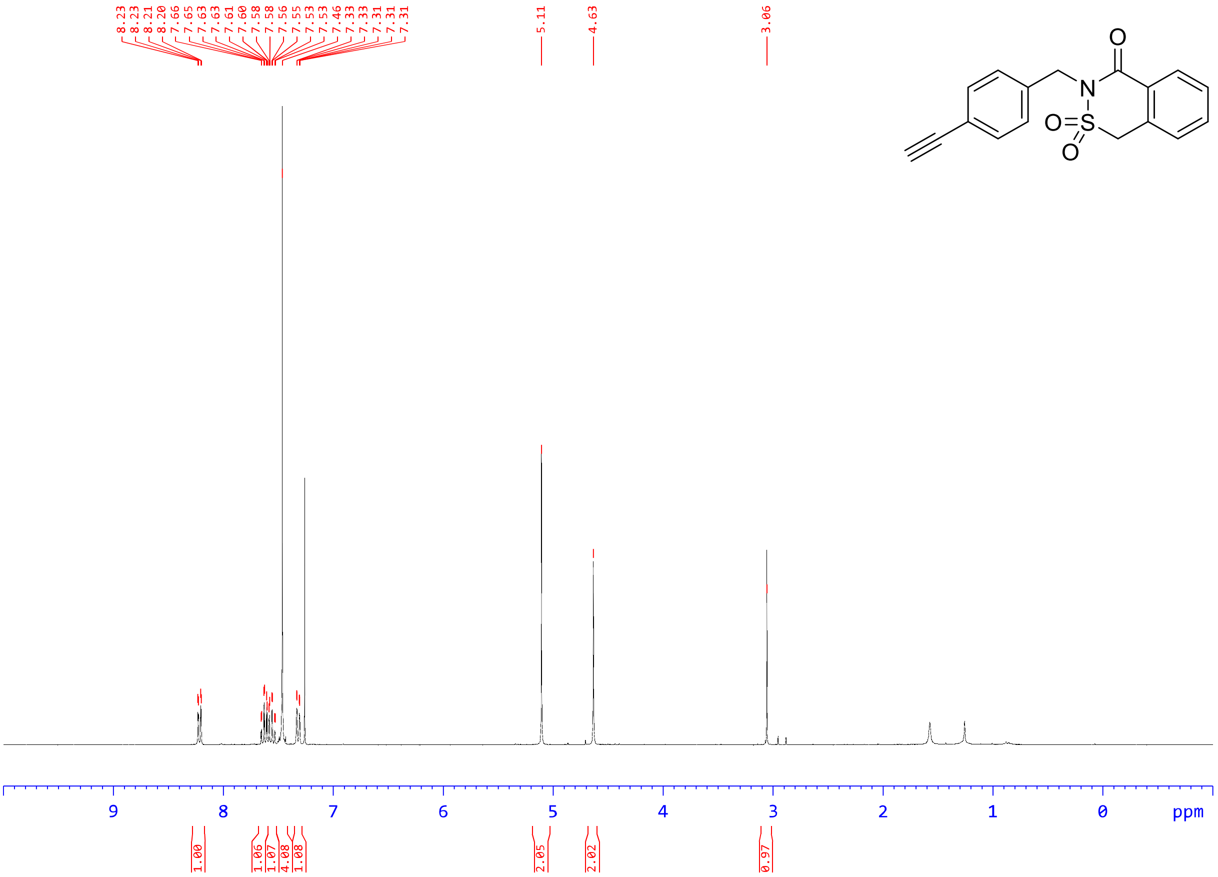


$^{13}$C{$^{1}$H}-NMR (75 MHz, $CDCl_3$)

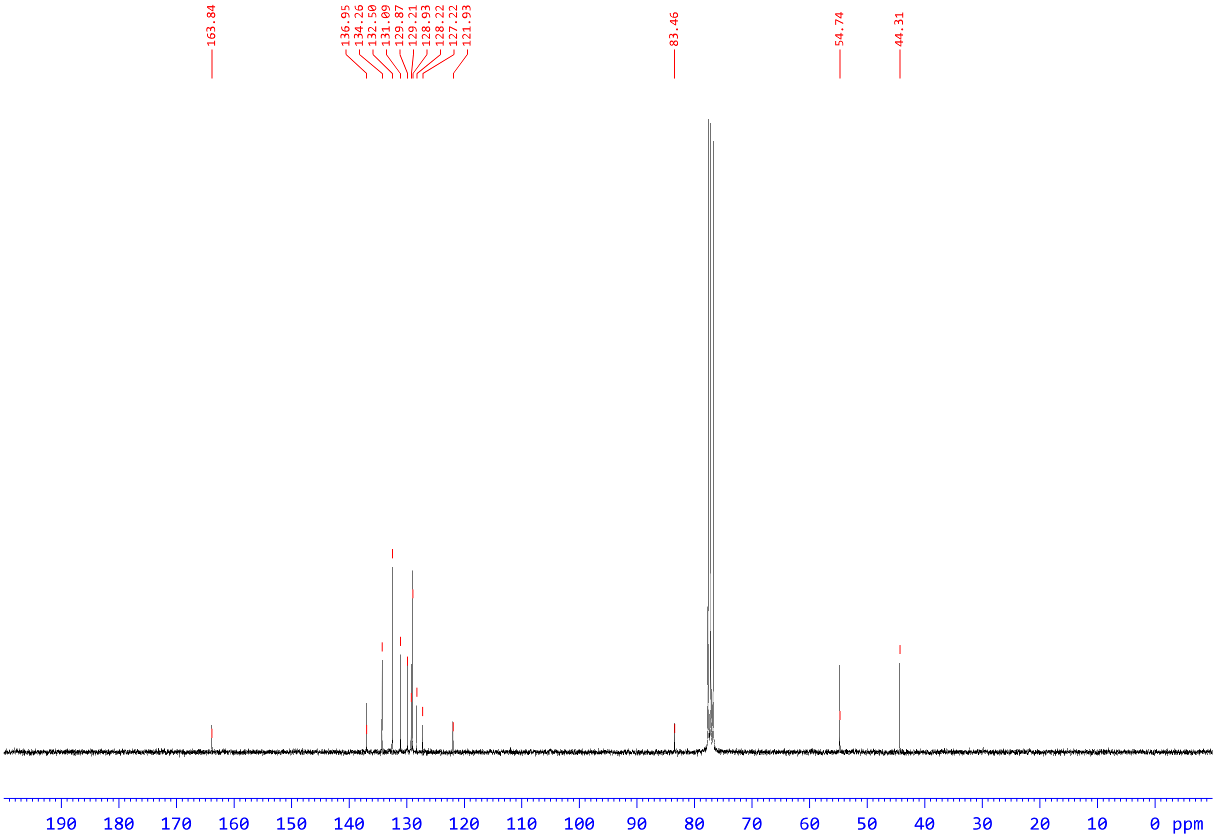

## 3.11. 1-bromo-3-(2-methylbenzyl)-1*H*-benzo[*d*][1,2]thiazin-4(3*H*)-one 2,2-dioxide (22)

$^{1}$H-NMR (300 MHz, $CDCl_3$)

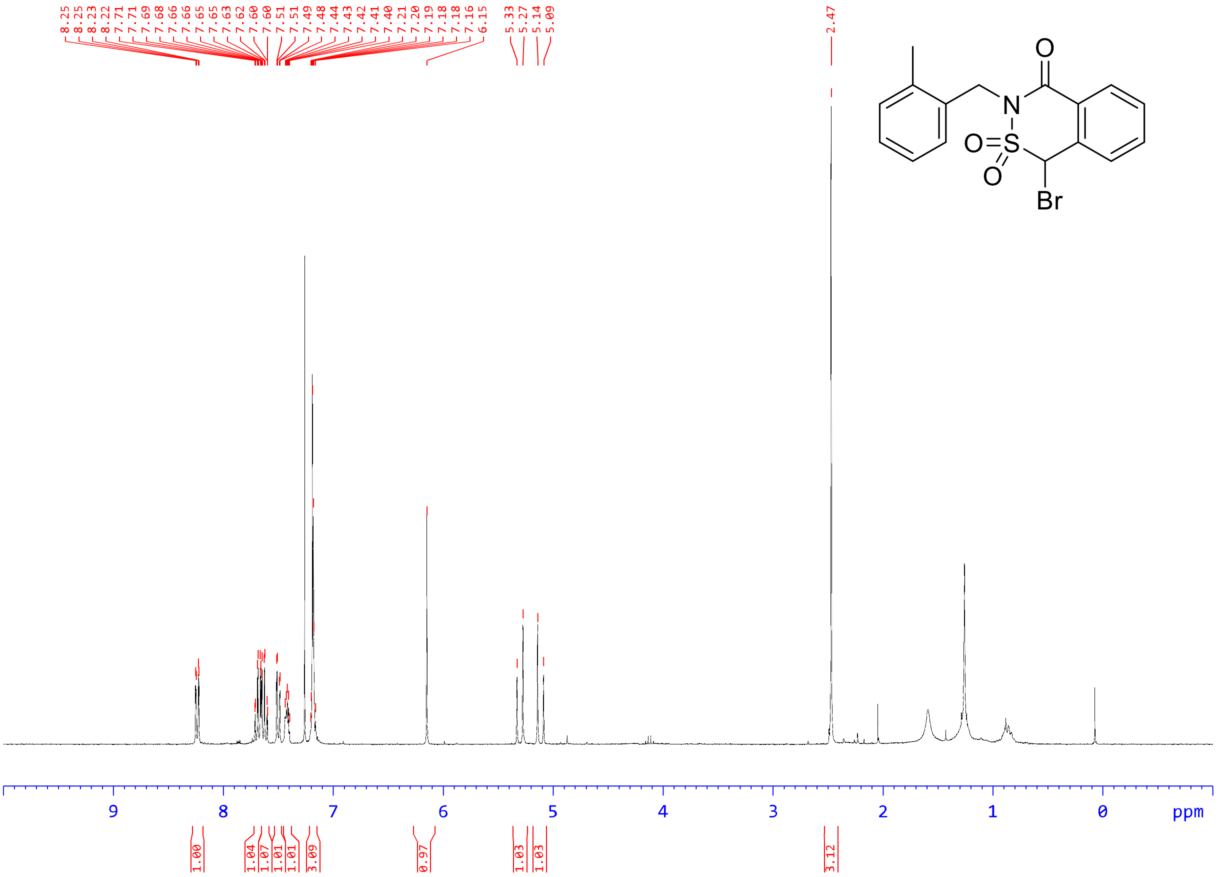


$^{13}$C{$^{1}$H}-NMR (75 MHz, $CDCl_3$)

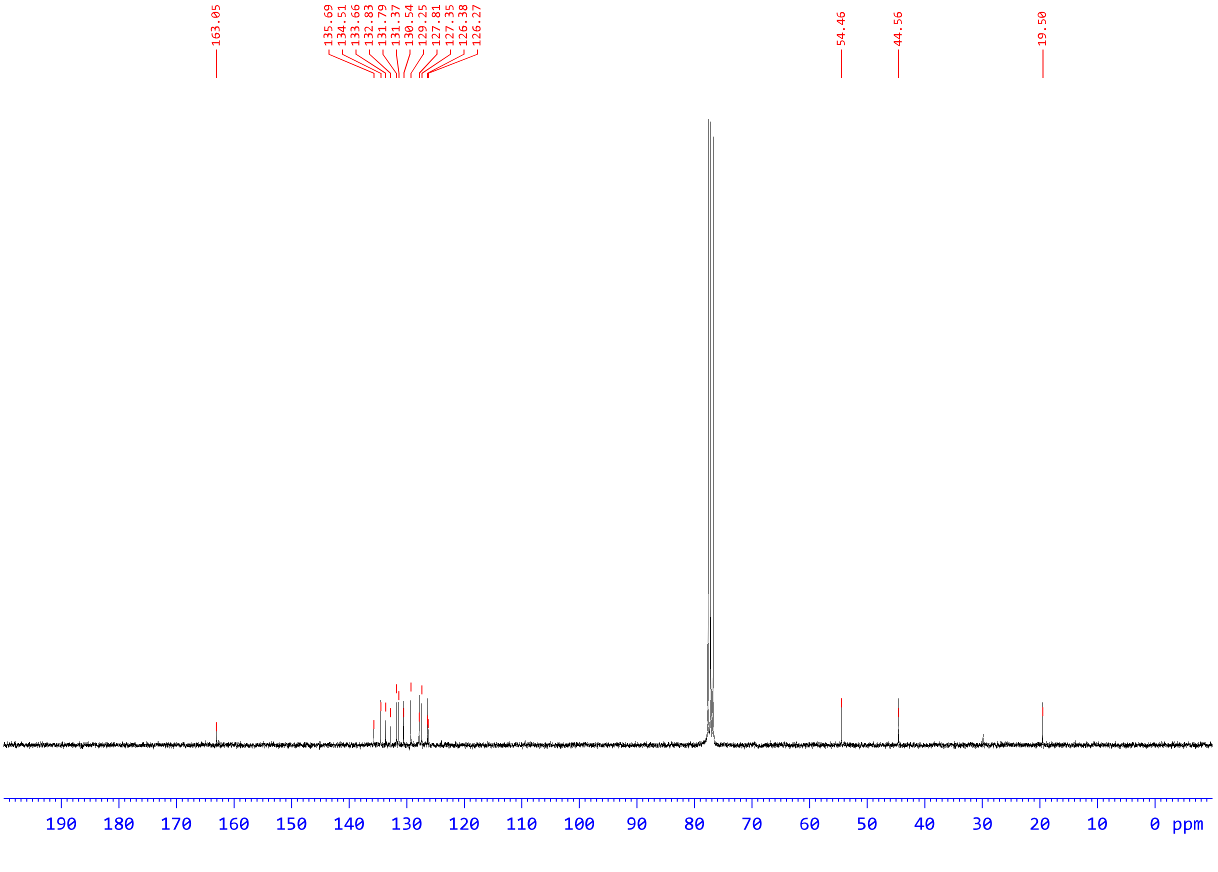

## 3.12. 1,3-bis(2-methylbenzyl)-1*H*-benzo[*d*][1,2]thiazin-4(3*H*)-one 2,2-dioxide (23)

$^{1}$H-NMR (300 MHz, $CDCl_3$)

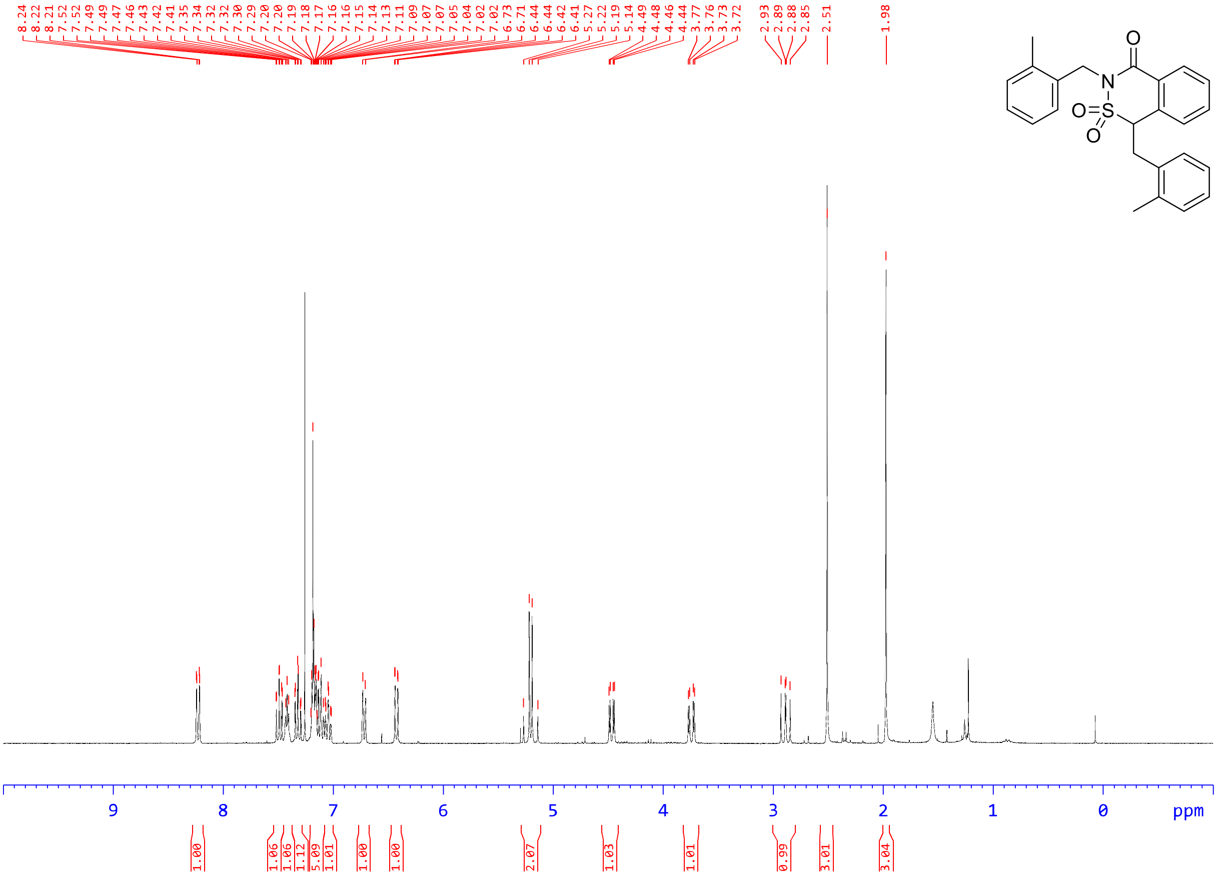


$^{13}$C{$^{1}$H}-NMR (75 MHz, $CDCl_3$)

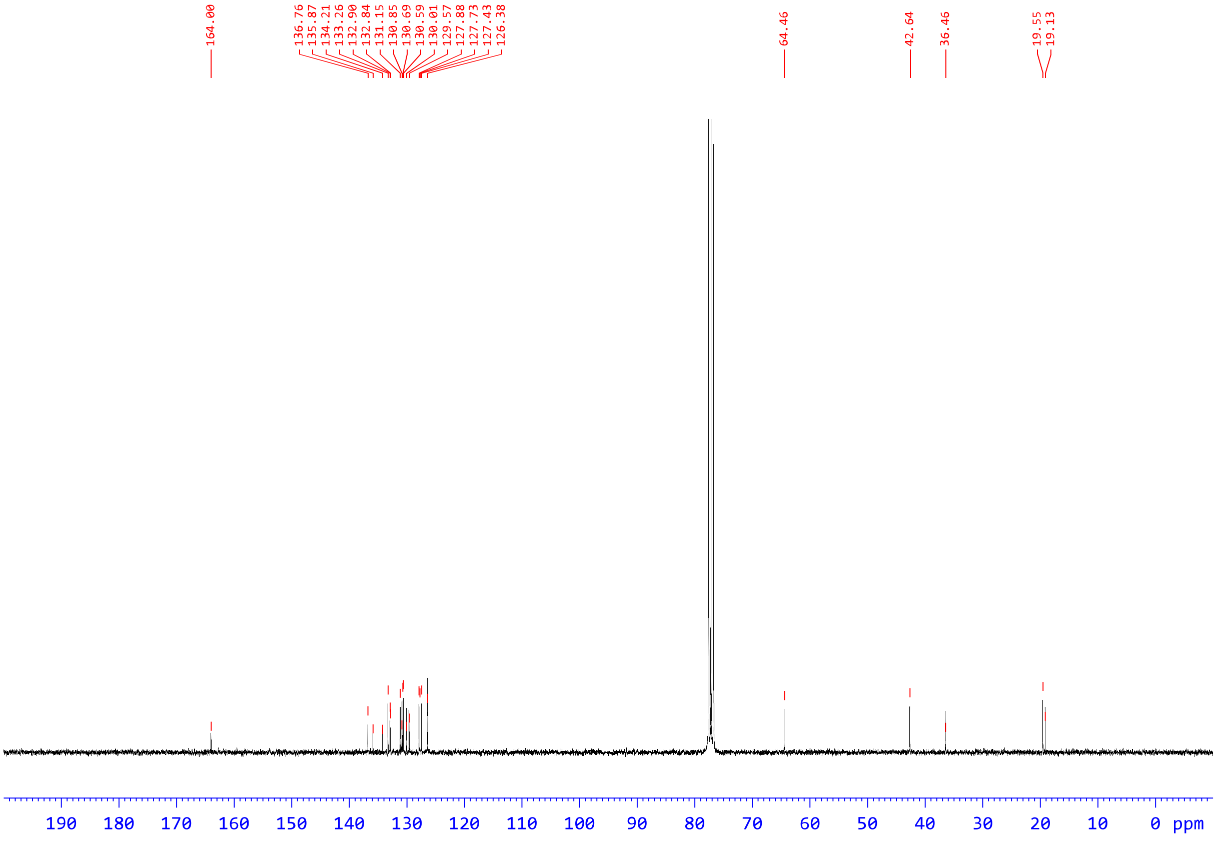

## 3.13. 3-(2-methylbenzyl)-1-(prop-2-yn-1-yl)-1*H*-benzo[*d*][1,2]thiazin-4(3*H*)-one 2,2-dioxide (25)

$^{1}$H-NMR (700 MHz, $CDCl_3$)

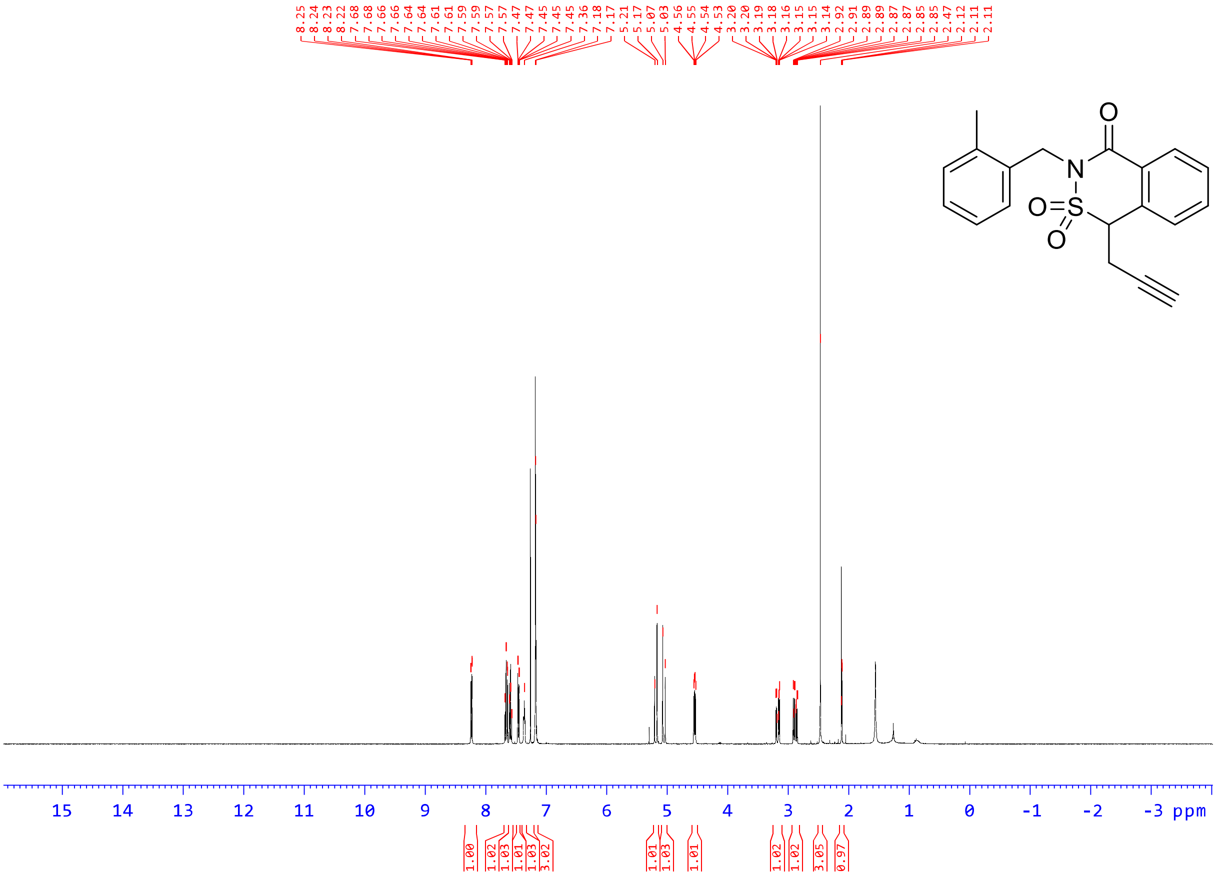


$^{13}$C{$^{1}$H}-NMR (175 MHz, $CDCl_3$)

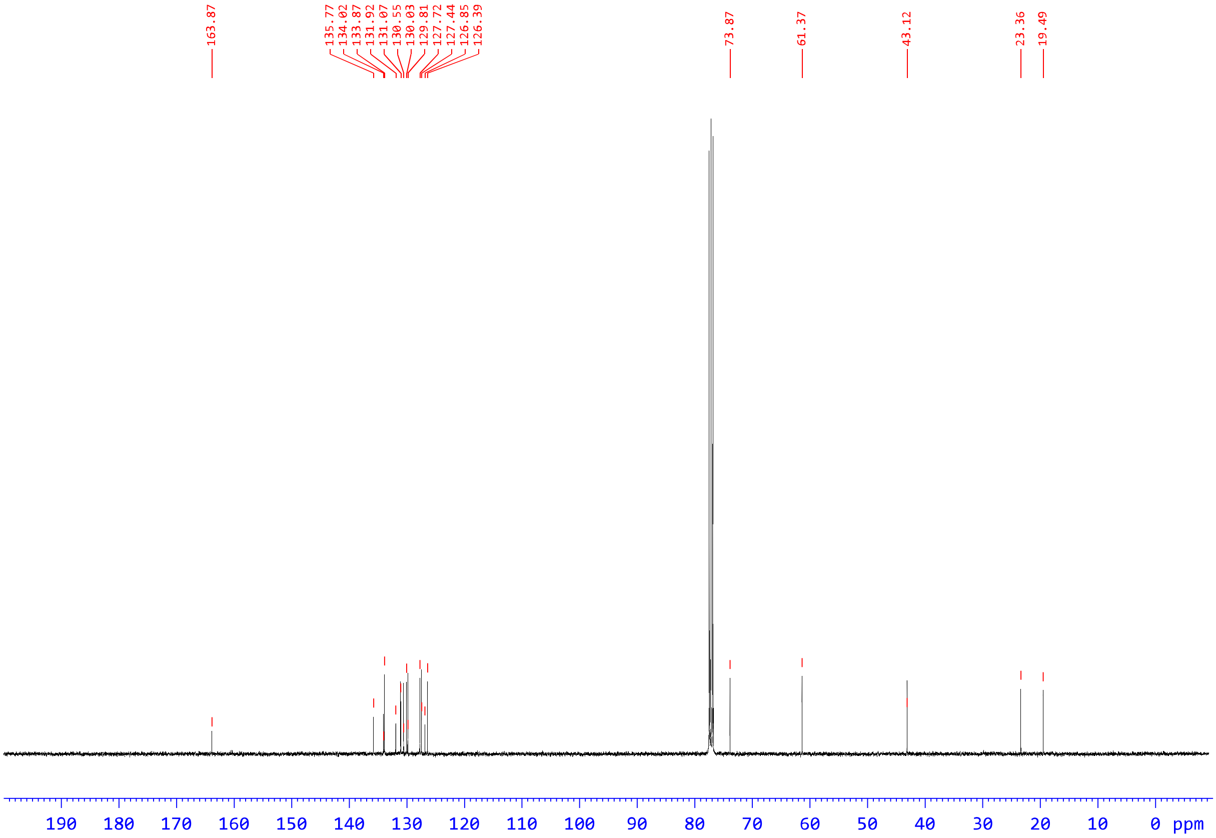


## 3.14. *Biotin-PEG3-click product* (27)

$^{1}$H-NMR (500 MHz, MeOD-d$_4$)

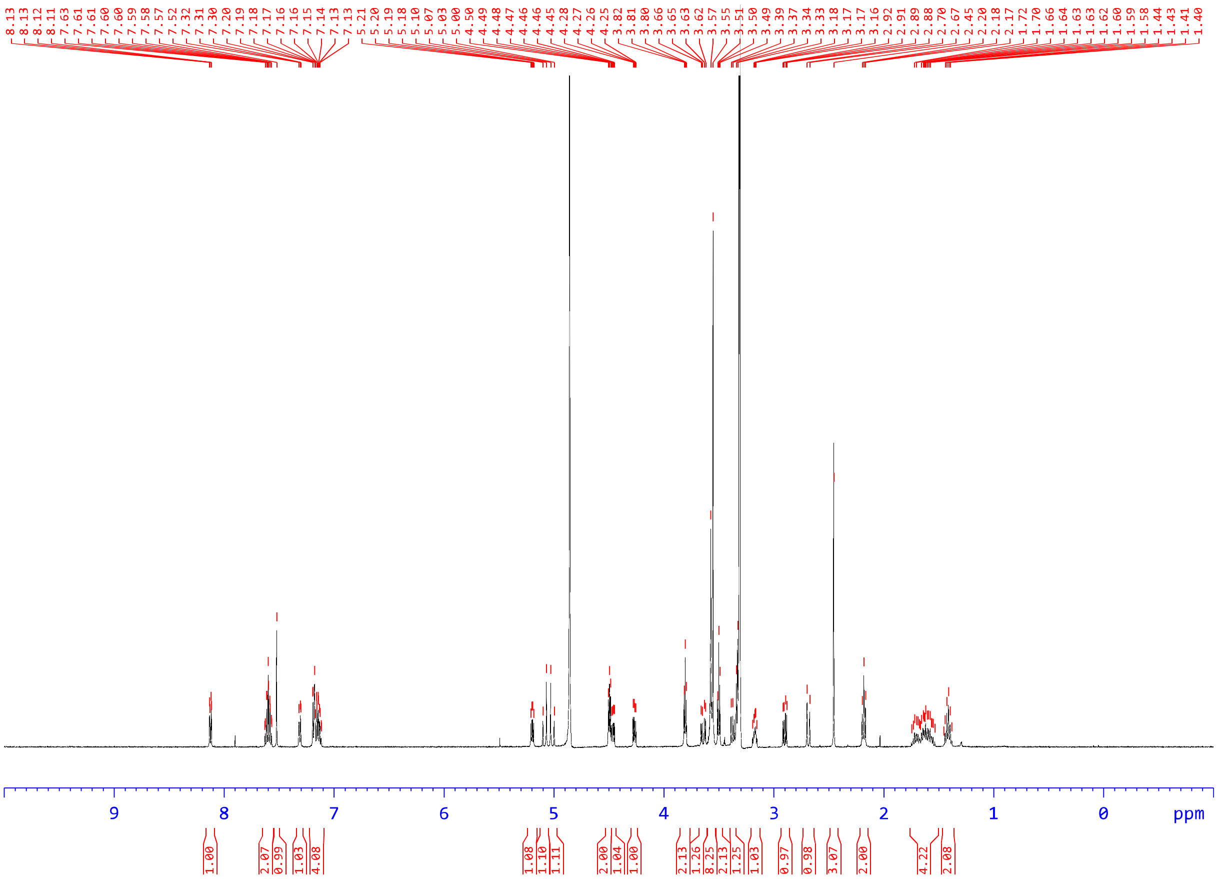

$^{13}C\{^{1}H\}$-NMR (100 MHz, MeOD-d$_4$)

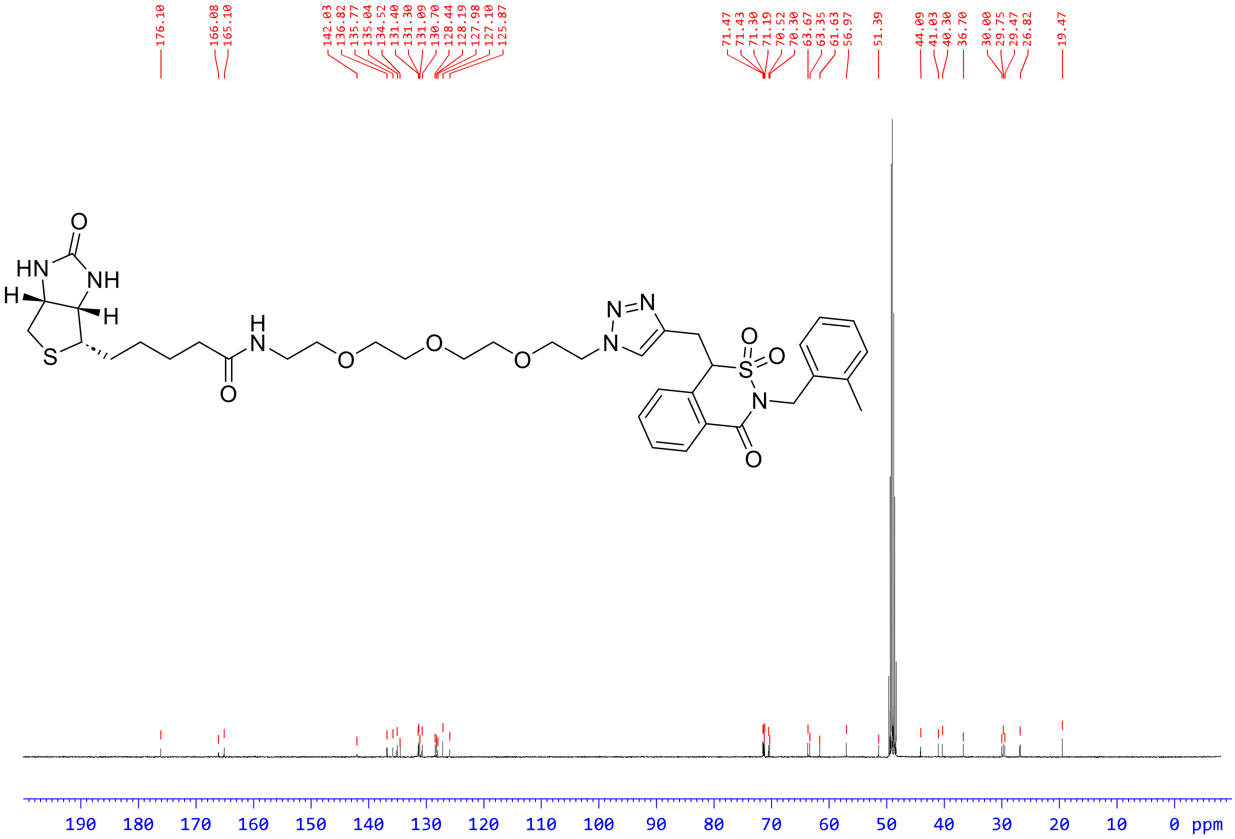

Supplement: Data S5 [file EMS200382-supplement-Data_S5.pdf]
