# Supplementary material for: SPL13 controls a root apical meristem phase change by triggering oriented cell divisions
Source: Science. Author manuscript; Available in PMC 2025 May 15. (PMC7616863; doi:10.1126/science.ado4298)
Supplement: Figures S1-24 [file EMS200382-supplement-Figures_S1_24.pdf]

Science
AAAS

Supplementary Materials for

SPL13 controls a root apical meristem phase change by triggering oriented cell divisions

Baojun Yang[1,2,3,4,†,*], Yanbiao Sun[1,2,†], Max Minne[1,2], Yanhua Ge[3,4], Qianru Yue[3,4], Vera Goossens[5,6], Eliana Mor[1,2,8], Brenda Callebaut[7], Kevin Bevernaege[7], Johan M. Winne[7], Dominique Audenaert[5,6] and Bert De Rybel[1,2,*]

Corresponding authors: bert.derybel@psb.vib-ugent.be and bjyang@genetics.ac.cn

The PDF file includes:

Figs. S1 to S24

Other Supplementary Materials for this manuscript include the following:

Data S1 to S5

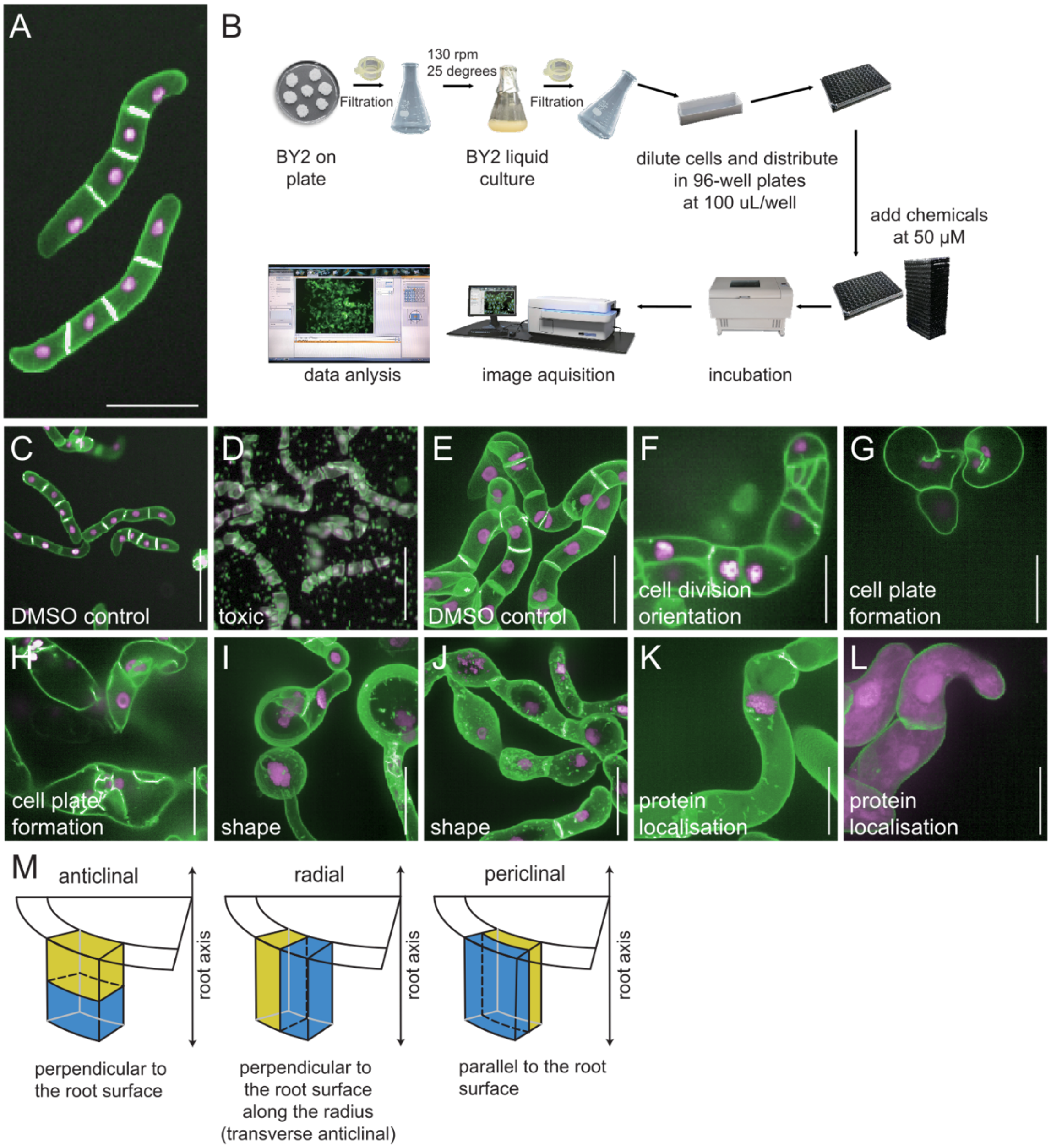

Fig. S1. Development of a microscopy-based chemical screen system to study cell division orientation in BY2 cell cultures
(**A**) Example BY2 filaments transformed with the dual color nuclear (p*35S*::H2B-mCherry, magenta) and a plasma membrane (p*35S*::CPK17-YFP, green) marker line for easy tracking in the automated chemical genetics screening. Scale bar is 200 µm. (**B**) Visual overview of the chemical genetics screening procedure. (**C**-**D**) Example of representative filaments of the dual color reporter line grown on control medium supplemented with DMSO as solvent for the chemical library (C) and medium supplemented with one of the toxic chemicals (D). Scale bar is 200 µm. (**E**-**L**) Example of representative filaments of the dual color reporter line grown on control medium supplemented with DMSO (E) and overview of various phenotypes observed in the chemical genetics screening (F-L). Scale bar is 50 µm. (**M**) Overview of the used nomenclature for oriented cell divisions in the root.

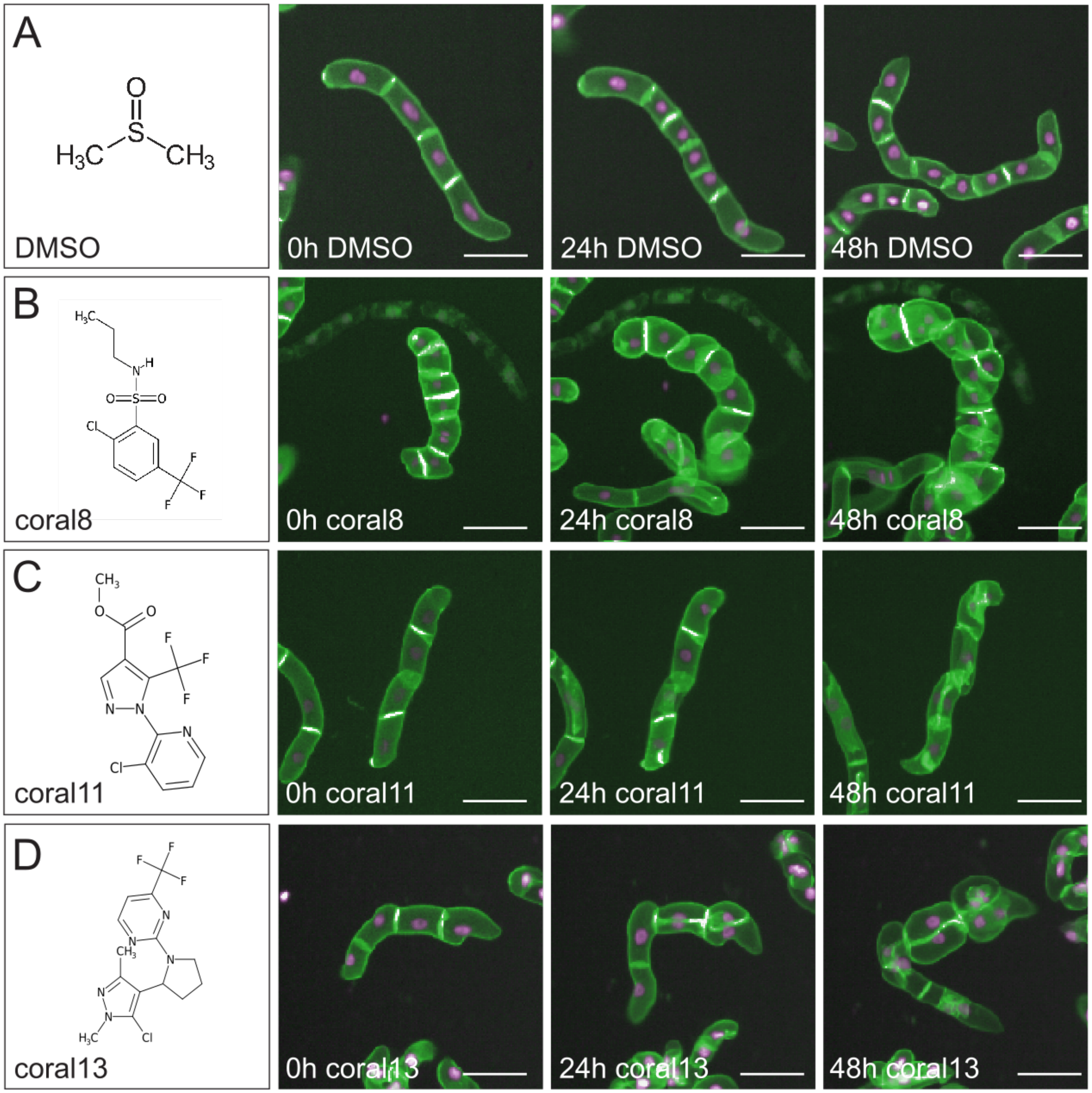

Fig. S2. Effect of the selected chemicals on division orientation in BY2 cell cultures
(**A**) Chemical structure of the DMSO solvent and control phenotypes on representative filaments of the BY2 cell culture over the course of 2 days. (**B-D**) Chemical structure of the selected chemicals and the respective phenotypical effects of a 50 µM treatment on a representative filament over time. Scale bars are 100 µm.

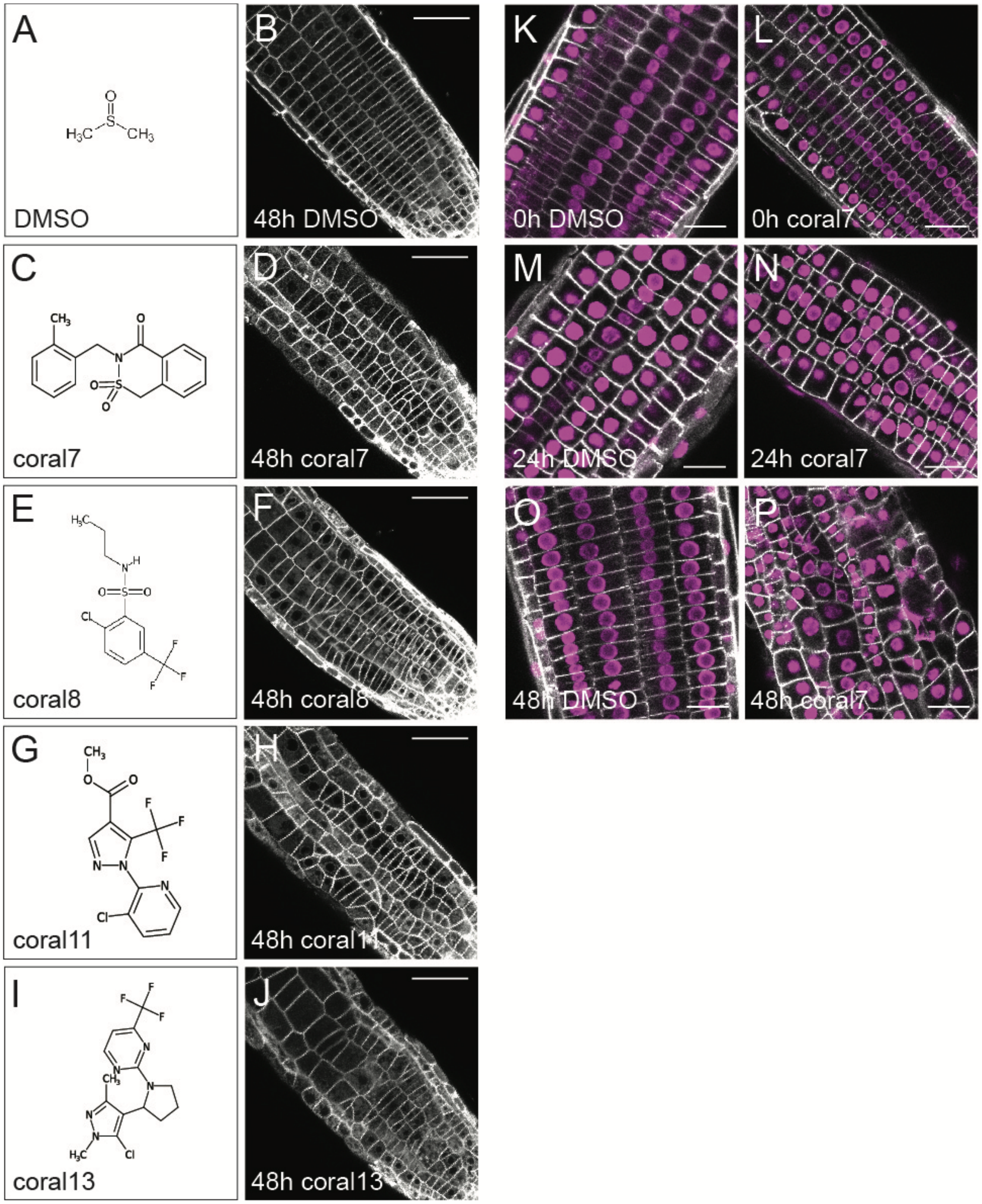

Fig. S3. Effect of the selected chemicals on division orientation in *Arabidopsis thaliana* root apical meristems

(**A**-**B**) Chemical structure of the DMSO solvent and control phenotypes on representative root apical meristems over the course of 2 days. (**C**-**J**) Chemical structure of the selected chemicals and the respective phenotypical effects of a 50 µM treatment on a representative root apical meristem after 2 days. Scale bars are 50 µm. (**K**-**P**) Effect of 50 µM coral7 treatment on root apical meristem cells at three timepoints compared to a control DMSO solvent treatment. Scale bars are 25 µm.

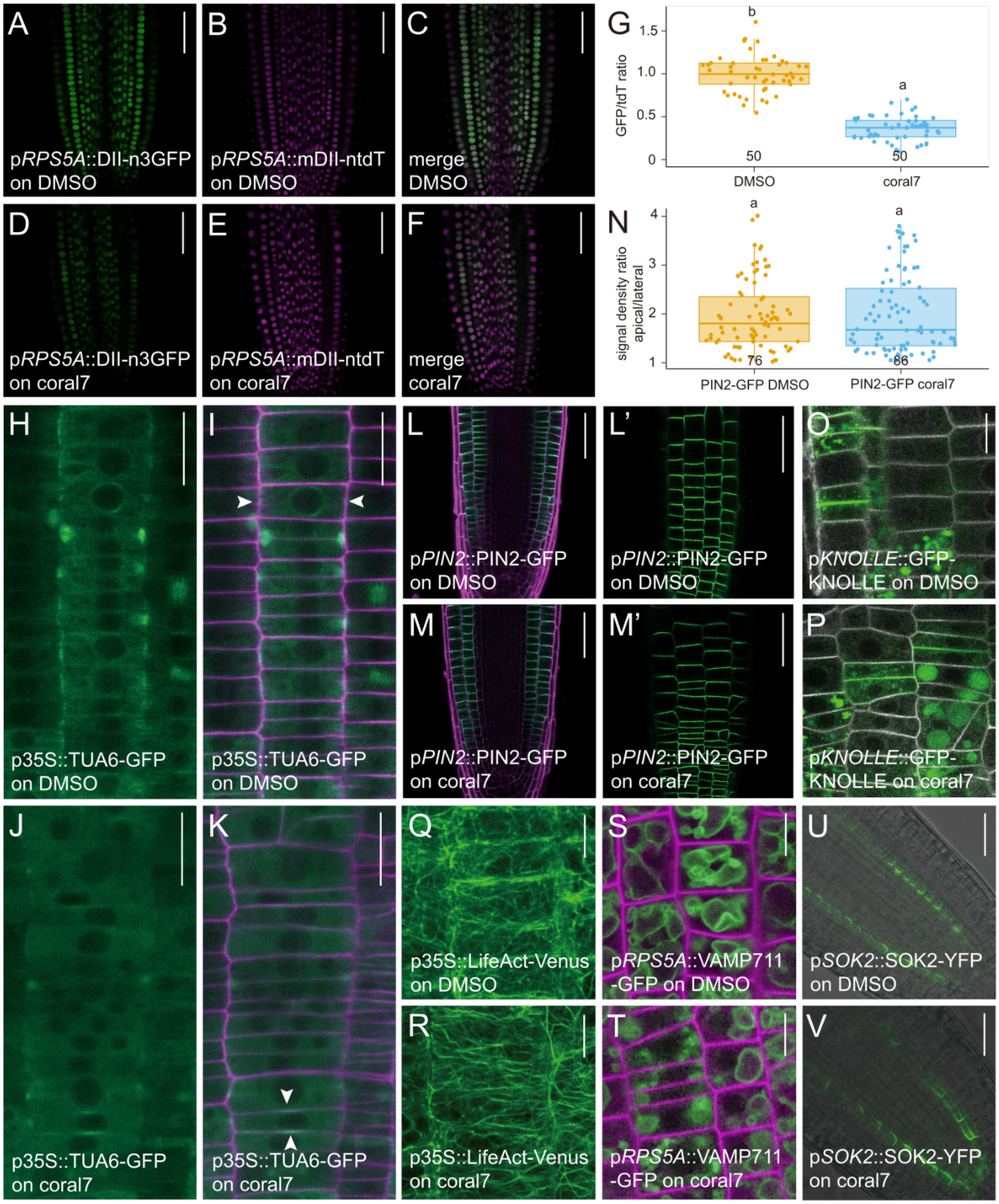

Fig. S4. Evaluation of coral7 treatment on known reporter lines in Arabidopsis roots
(**A-F**) Confocal images of 7 DAG root meristems expressing R2D2 auxin sensor under 24 hours treatment in DMSO control and coral7 (50 µM). (**G**) Quantification of auxin signaling by GFP/RFP ratio in cortical cells of DMSO control and coral7 (A-F). (**H-K**) Confocal images of 7 DAG root meristems expressing p*35S*::TUA6-GFP and PPB position in DMSO and Coral7 treatment (48hours) as indicated (white arrow), and counterstained with propidium iodide

(magenta). (**L**-**M**) Confocal images of 7 DAG root meristems expressing p*PIN2*::PIN2-GFP under 24 hours treatment in DMSO control and Coral7 (50 µM), and counterstained with propidium iodide (magenta). L' and M' represent epidermal PIN2-GFP expression used for quantification. (**N**) Quantification of GFP signal ratio (apical/lateral) in p*PIN2*::PIN2-GFP epidermis cells under 24 hours treatment with DMSO control and coral7 (50 µM) (L-M). (**O**-**V**) Effect of a 50 µM coral7 treatment on root cells of Actin filament (p*35S*::LifeAct-Venus) (Q-R), cell plate (p*KNOLLE*::GFP-KNOLLE) (O-P), vacuolar morphology (p*RPS5A*::VAMP711-GFP) (S-T) and endodermis cell polarity marker (*pSOK2*::SOK2-YFP) (U-V). Scale bars are 50 µm in (A-F), 20 µm in (H-K) and 10 µm in (O-T) and 25 µm in (U-V).

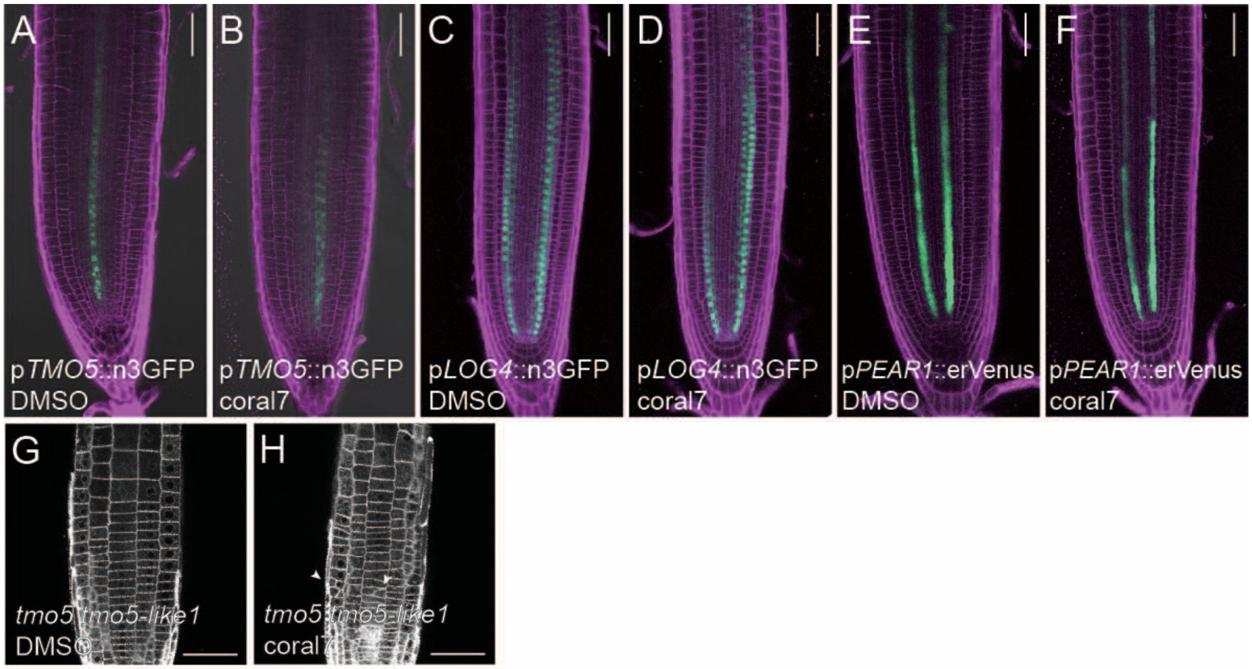

Fig. S5. Expression pattern of TMO5, LOG4 and PEAR1 upon coral7 treatment
(**A**-**F**) Confocal images of 7 DAG root meristems expressing of p*TMO5*::n3GFP, p*LOG4*::n3GFP and p*PEAR1*::erVenus with 24 hours DMSO and coral7 (50 µM) treatment and counterstained with propidium iodide (magenta). (**G**-**H**) Confocal images of *tmo5 tmo5-like1* double mutant root meristems with 48 hours DMSO and coral7 (50 µM) treatment and counterstained with propidium iodide (white). Arrowheads indicate the induced ectopic cell divisions. Scale bar is 50 µm.

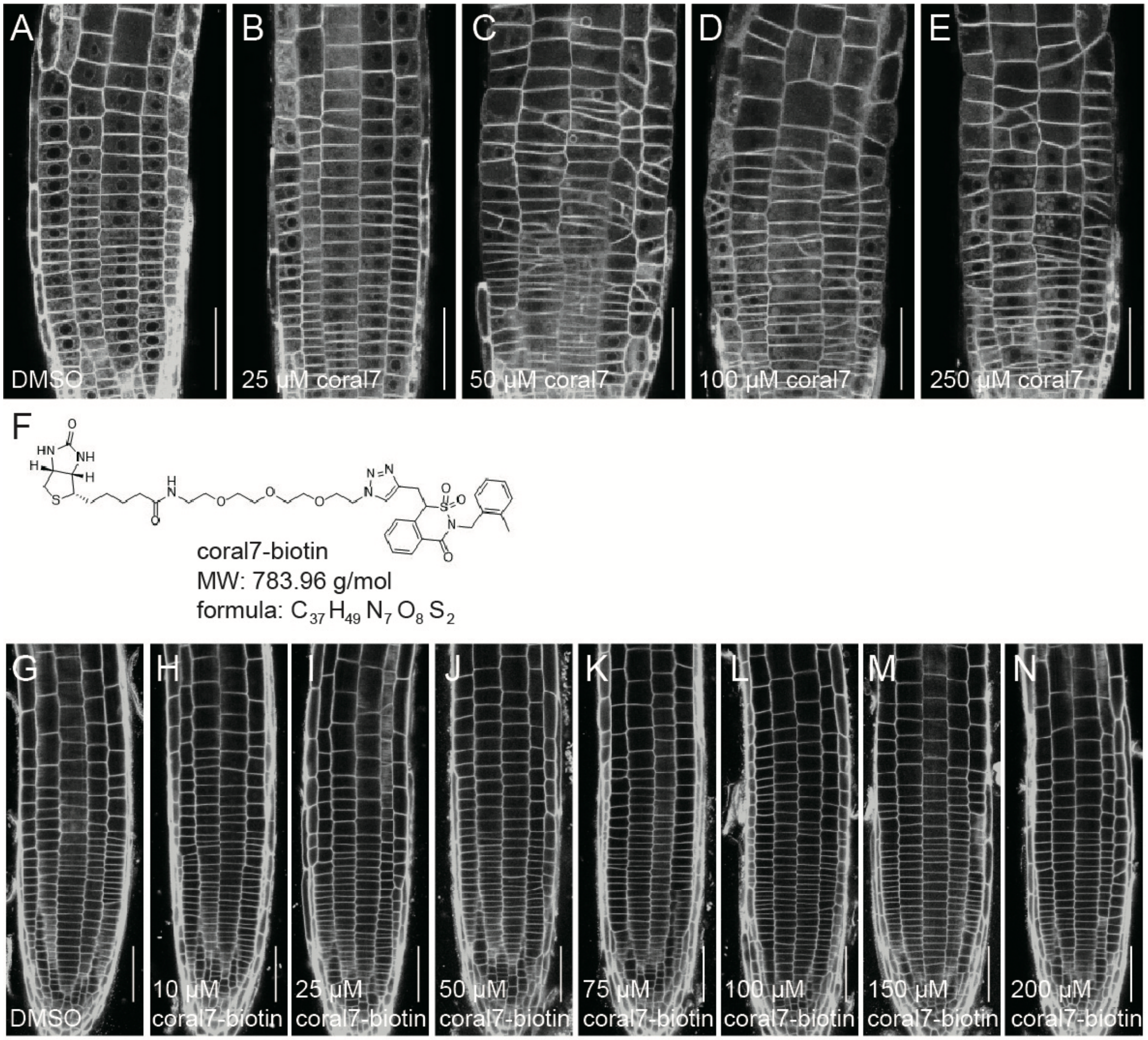

Fig. S6. Dose-response analysis and activity of a biotin labelled version of coral7
(**A**-**E**) Confocal images of Col-0 root meristems counterstained using PI and treated for 48 hours with the indicated concentration of coral7. (**F**) Chemical structure of the biotinylated-coral7 molecule. (**G**-**N**) Confocal images of 5 DAG Col-0 root meristems counterstained using PI and treated for 3 days with the indicated concentration of coral7-biotin. Scale bars are 50 µm.

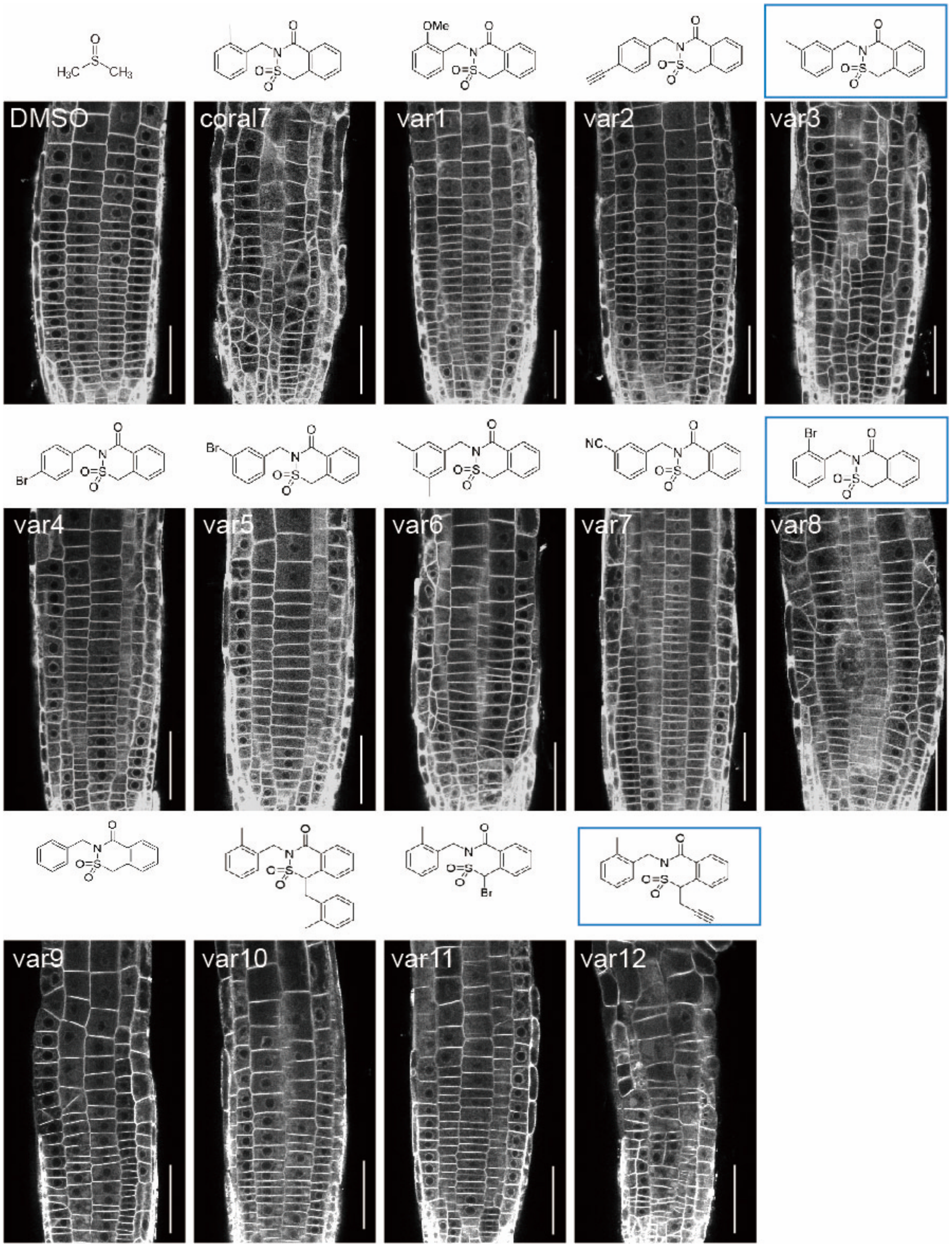

Fig. S7. Structure-activity relationship (SAR) analysis for coral7
Panels show the chemical structure of the tested coral7 variant and the resulting phenotypical effect of a 48 hours treatment at 50 µM on root apical meristems of *Arabidopsis thaliana*. Note that most modification result in a non-functional version of coral7 and only minor modification are tolerated.

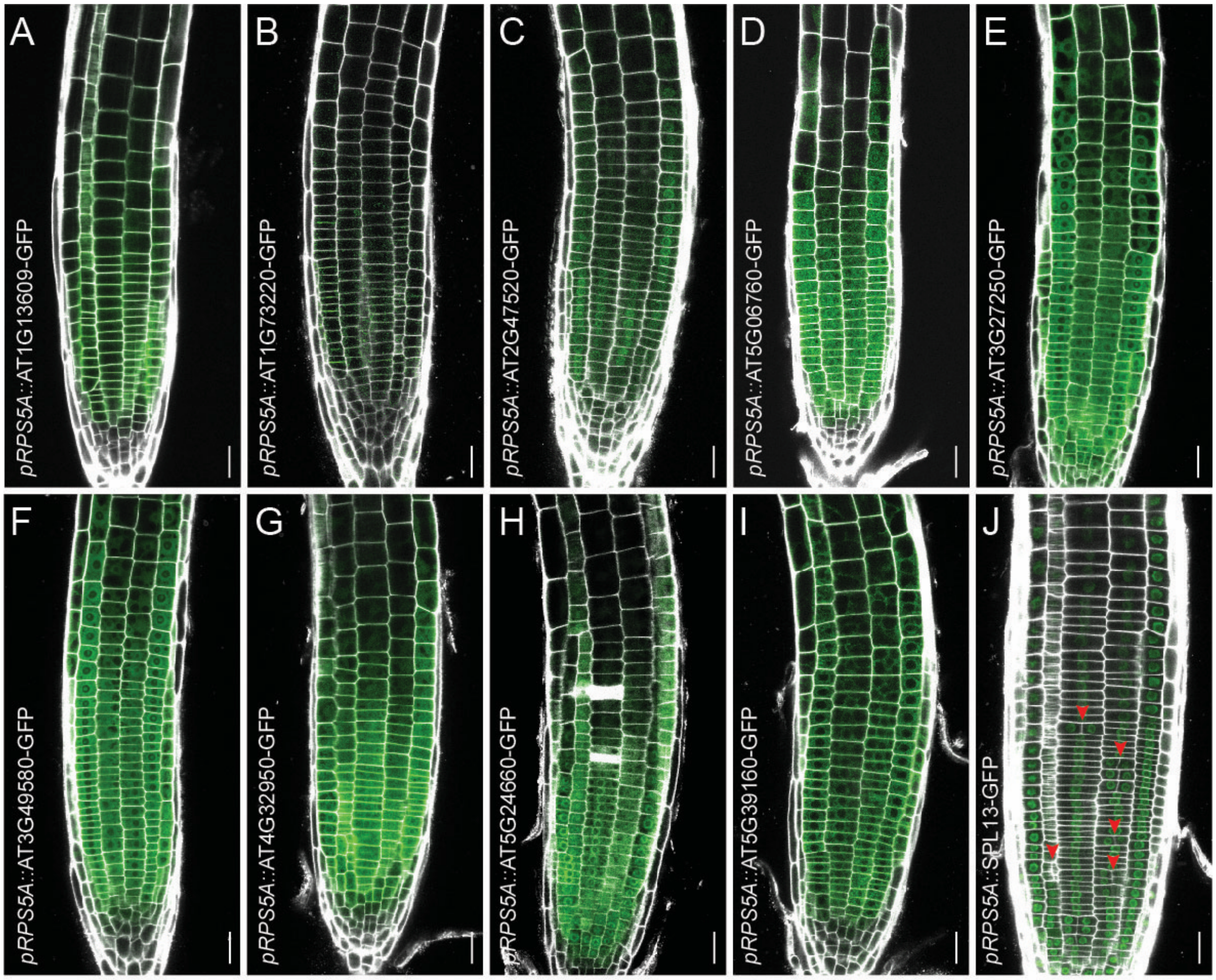

Fig. S8. Effect of misexpression of the top DEG from the RNA-seq analysis
(**A-J**) Confocal images of 7 DAG root meristems of the indicated genotypes counterstained using PI. Arrowheads indicate the induced ectopic periclinal/radial cell divisions in (J). Scale bar is 25 µm.

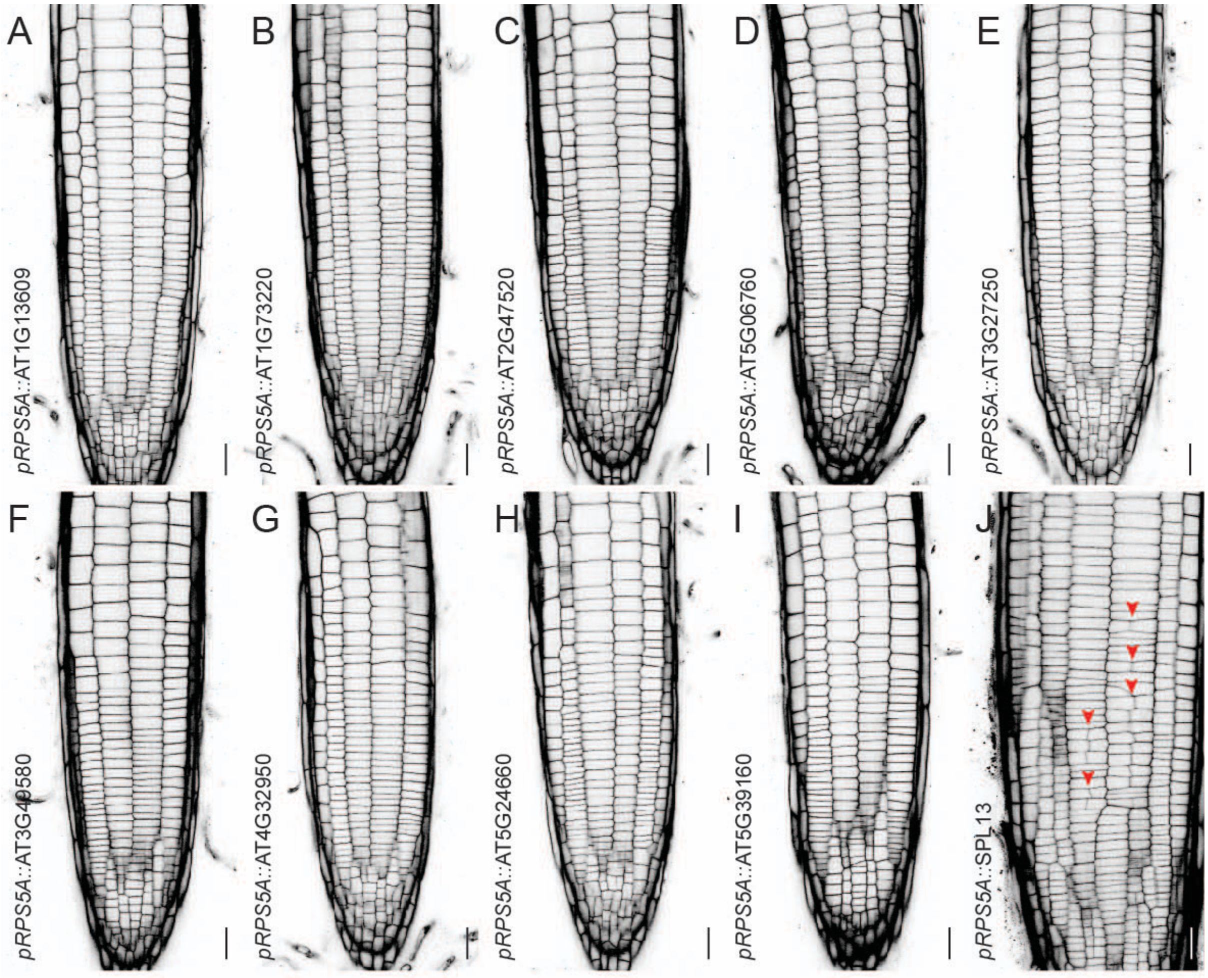

Fig. S9. Effect of misexpression of the top DEG from the RNA-seq analysis without GFP tag

(**A-J**) Confocal images of 9 DAG root meristems of the indicated genotypes counterstained using propidium iodide (black). Arrowheads indicate the induced ectopic periclinal/radial cell divisions in (J). Scale bar is 25 μm.

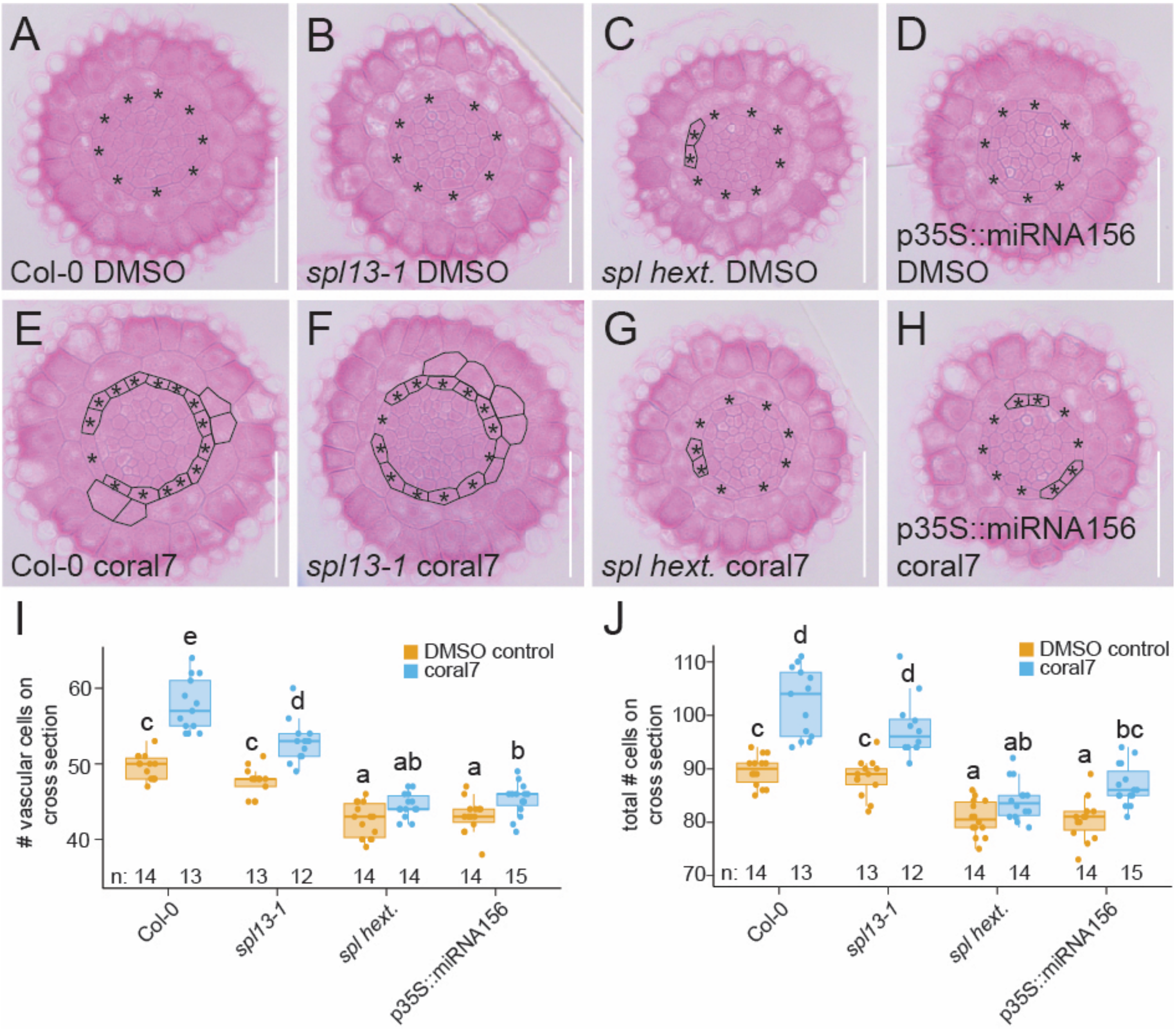

Fig. S10. The effect of coral7 is dependent on SPL levels

(**A-H**) Histochemical cross sections through the root meristem of 6 DAG seedlings grown on control medium (A-D) or medium supplemented with 50 µM coral7 for 48 hours (E-H). Black asterisks indicate the endodermis; black outlines indicate ectopic divisions in the cortex and endodermis cell layers. Scale bar is 50 µm. (**I-J**) Quantification of the total cell numbers (I) and the vascular cell numbers (J) in panels A-H.

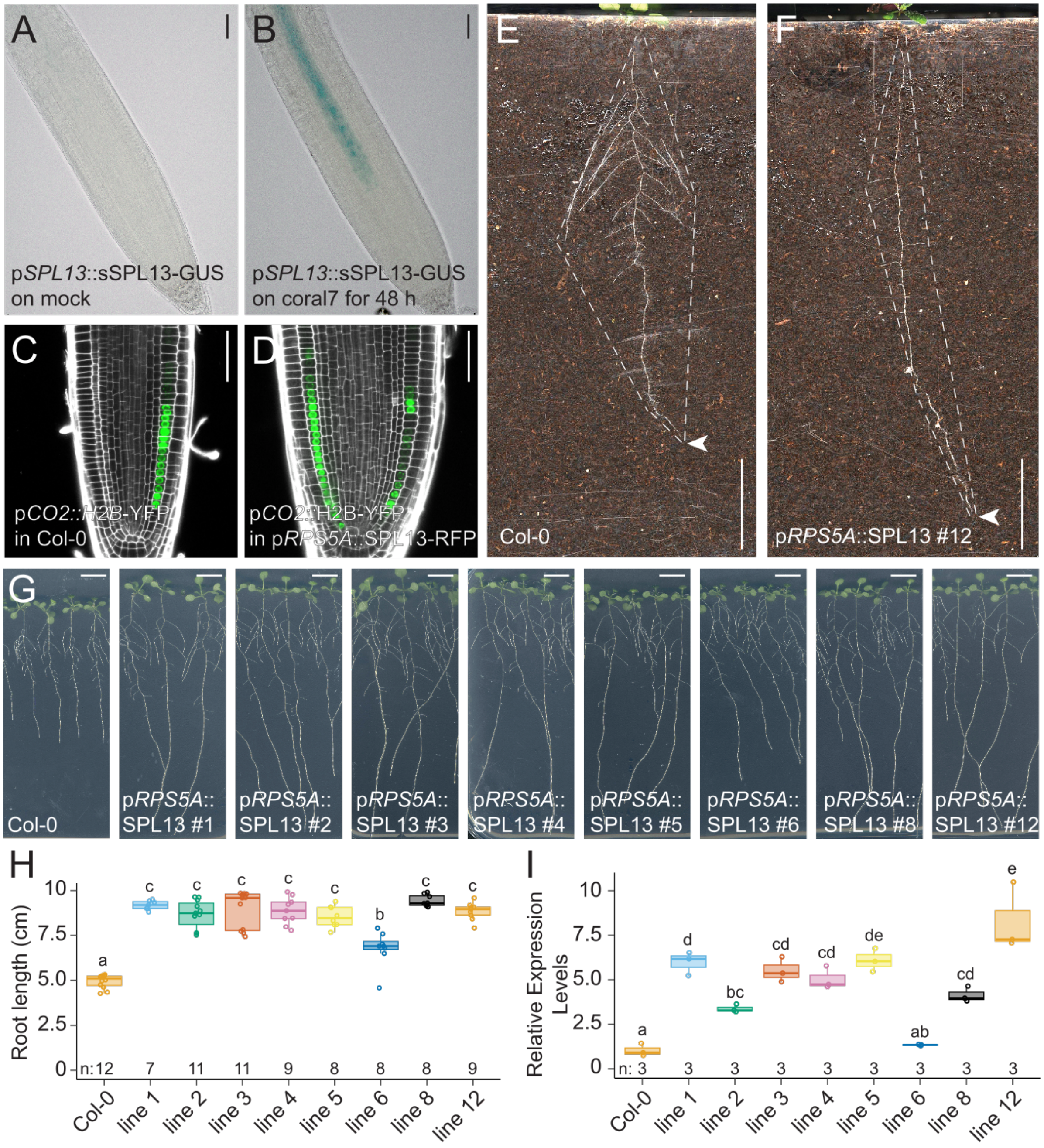

Fig. S11. Characterization of the p*RPS5A*::SPL13-GFP line

(**A**-**B**) Localization of p*SPL13*::sSPL13-GUS in 8 DAG root meristems grown on DMSO solvent control medium, or medium supplemented with 50 µM coral7 for 24 hours. Scale bar is 50 µm. (**C**-**D**) Confocal images of 8 DAG root meristems expressing p*CO2*::H2B-YFP (green nuclei) in the respective genotypes as indicated and counterstained with propidium iodide (white). Scale bar is 50 µm. (**E**-**F**) Seedling phenotype of Col-0 and p*RPS5A*::SPL13-GFP seedlings grown in a rhizotron for 17 days. Scale bar is 2 cm; Arrowhead indicates position of the root apical meristem; dashed line shows outline of the root system. (**G**) Seedling phenotypes of multiple independent p*RPS5A*::SPL13-GFP misexpression lines compared to the Col-0 control. Scale bar is 1cm. (**H**) Quantification of the root length in (G). (**I**) Relative expression levels of *SPL13* as determined by qRT-PCR in the different lines shown in (G).

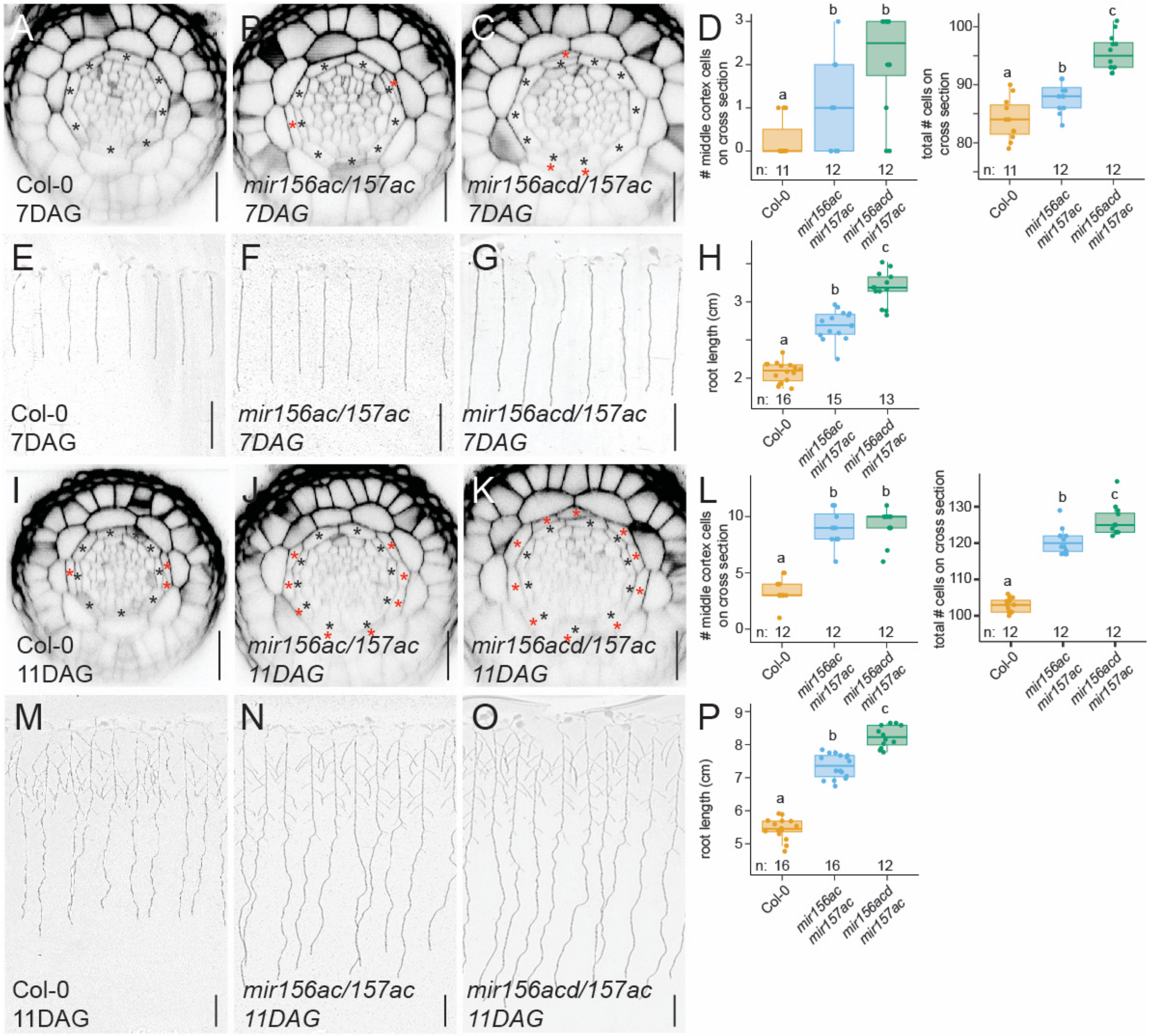

Fig. S12. *mir156/157* loss-of-function lines accelerate the juvenile to adult transition (**A-C** and **I-K**) Optical confocal cross section through respectively 7 DAG and 11 DAG root apical meristem of the indicated genotypes. Black asterisks indicate the endodermis cell; red asterisk indicate the middle cortex cells. (**D** and **L**) Quantifications of the cell numbers in (A-C and I-K) respectively. (**E-G** and **M-O**) Root length of 7 DAG and 11 DAG seedlings of the indicated genotypes. (**H** and **P**) Quantifications of the primary root length in (E-G and M-O) respectively. Scale bars are 25 µm in (A-C) and (I-K), 1cm in (E-G) and (M-O).

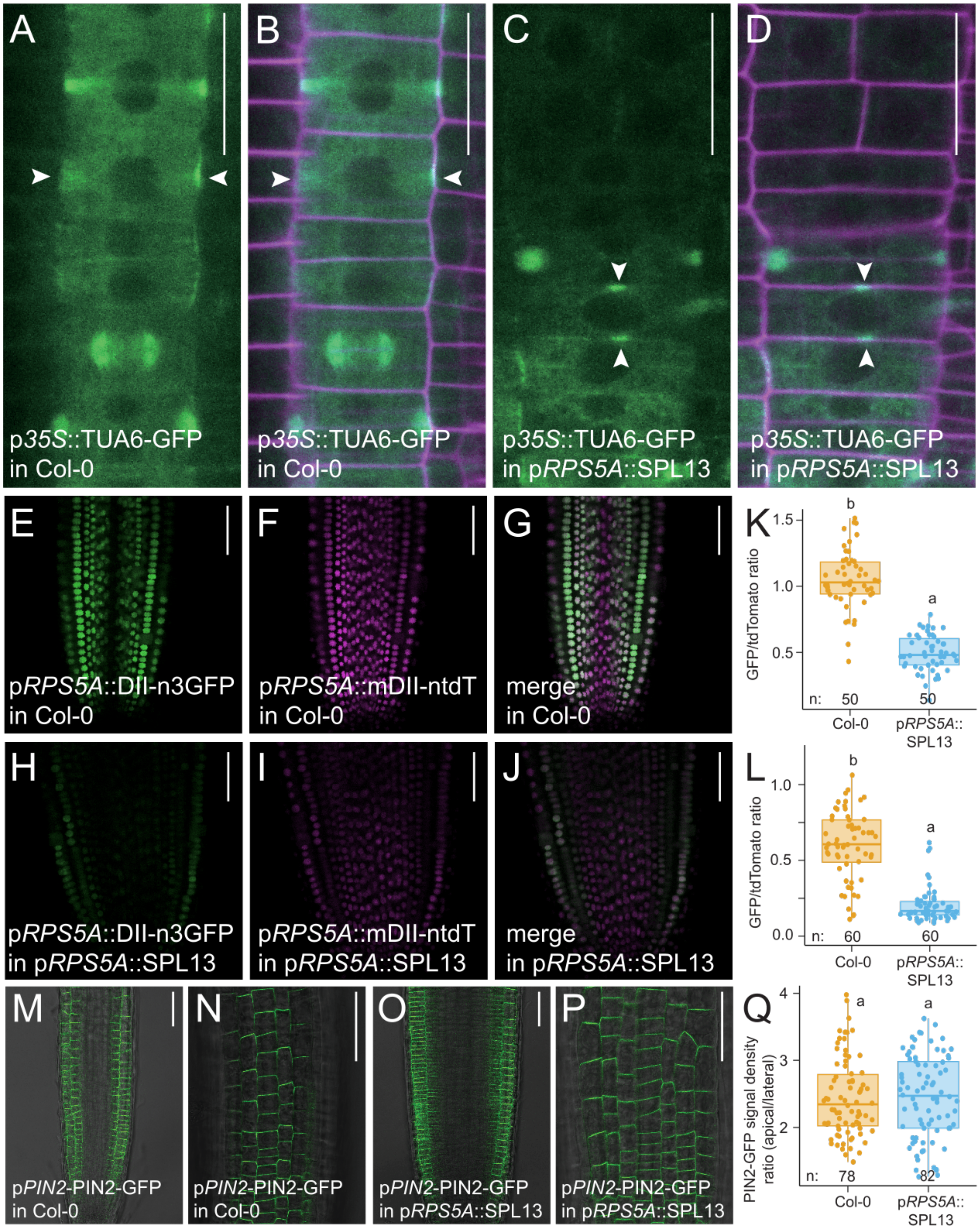

Fig. S13. Evaluation of SPL13 misexpression on known reporter lines in Arabidopsis roots
(**A-D**) Confocal images of 7 DAG root meristems expressing p*35S*::TUA6-GFP and PPB position as indicated (white arrow) in wild type and p*RPS5A*::SPL13 lines, and counterstained

with propidium iodide (magenta). (**E-J**) Confocal images of 7 DAG root meristems expressing R2D2 auxin sensor in wild type and p*RPS5A*::SPL13 lines.
(**K-L**) Quantifications of (E-J) auxin signaling by GFP/RFP ratio in cortical cells (K) and vascular cells (L) of wild type and p*RPS5A*::SPL13 lines. (**M-P**) confocal images of 7 DAG root meristems with central cell layers (M, O) or epidermal cell layers (N, P) in focus of p*PIN2*::PIN2-GFP in Col-0 and p*RPS5A*::SPL13-RFP backgrounds. (**Q**) Quantification of PIN2-GFP signal ratio (apical/lateral) in epidermis cells of the indicated genotypes (N, P). Scale bars are 20 μm in (A-D), 50 μm in (E-J).

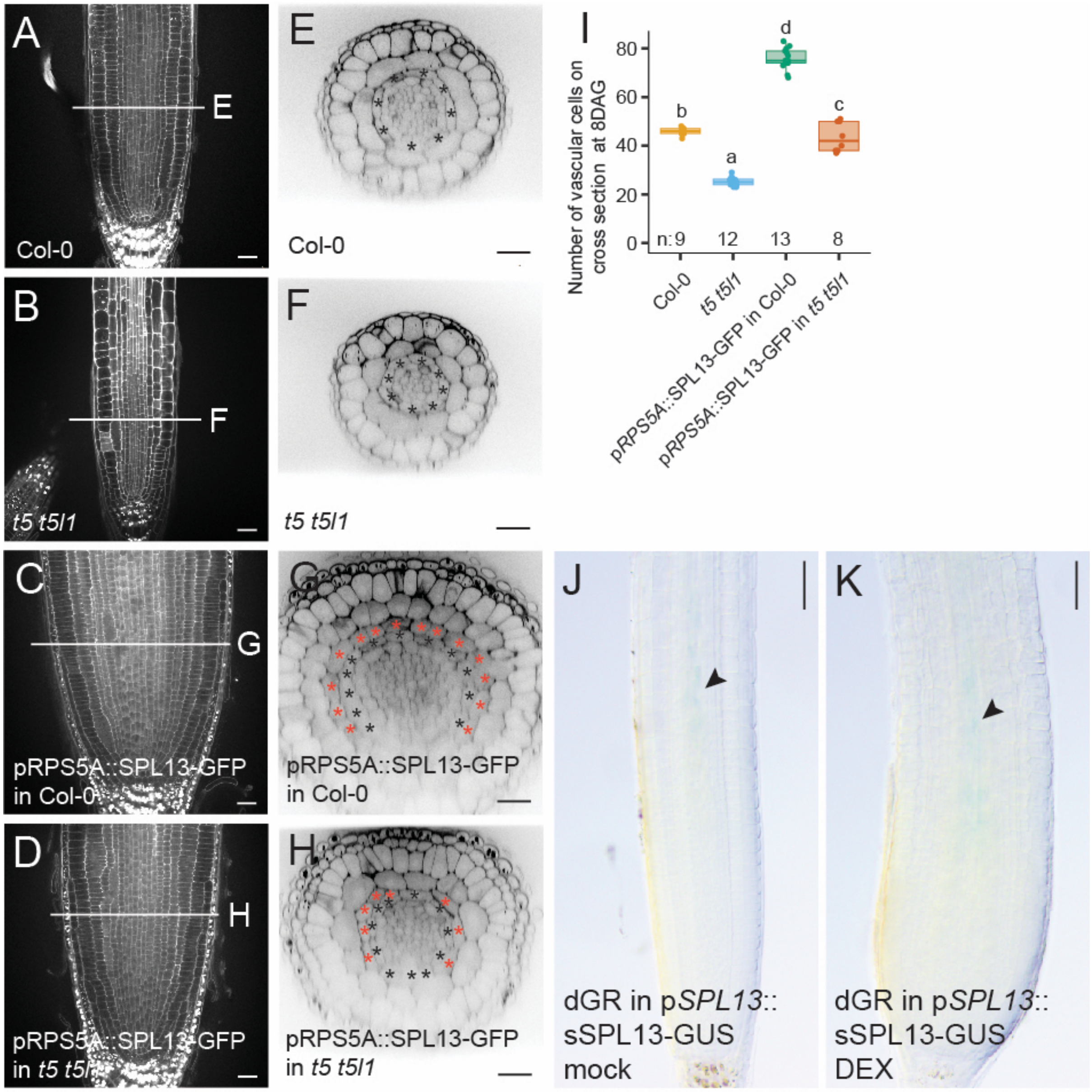

Fig. S14. SPL13 function does not require TMO5 activity

(**A-D**) Confocal images of 8 DAG root meristems of the indicated genotypes counterstained using mPS-PI. (**E-H**) Optical confocal cross sections of root meristems of the indicated genotypes counterstained using mPS-PI at the position indicated in A-D. Scale bar is 25 µm. (**I**) Quantification of the number of vascular cells on a cross section in (E-H). (**J-K**) Light microscopy image of 9 DAG root meristem expressing p*SPL13*::sSPL13-GUS in dGR background, grown on DMSO solvent control medium, or medium supplemented with 10 µM DEX. Black asterisks in (E-H) indicate the endodermis cell layer; red asterisks additional ground tissue layer. Black arrowheads in (J-K) indicate the weak GUS signal. Scale bars are 25 µm in (A-H), 50 µm in (J-K).

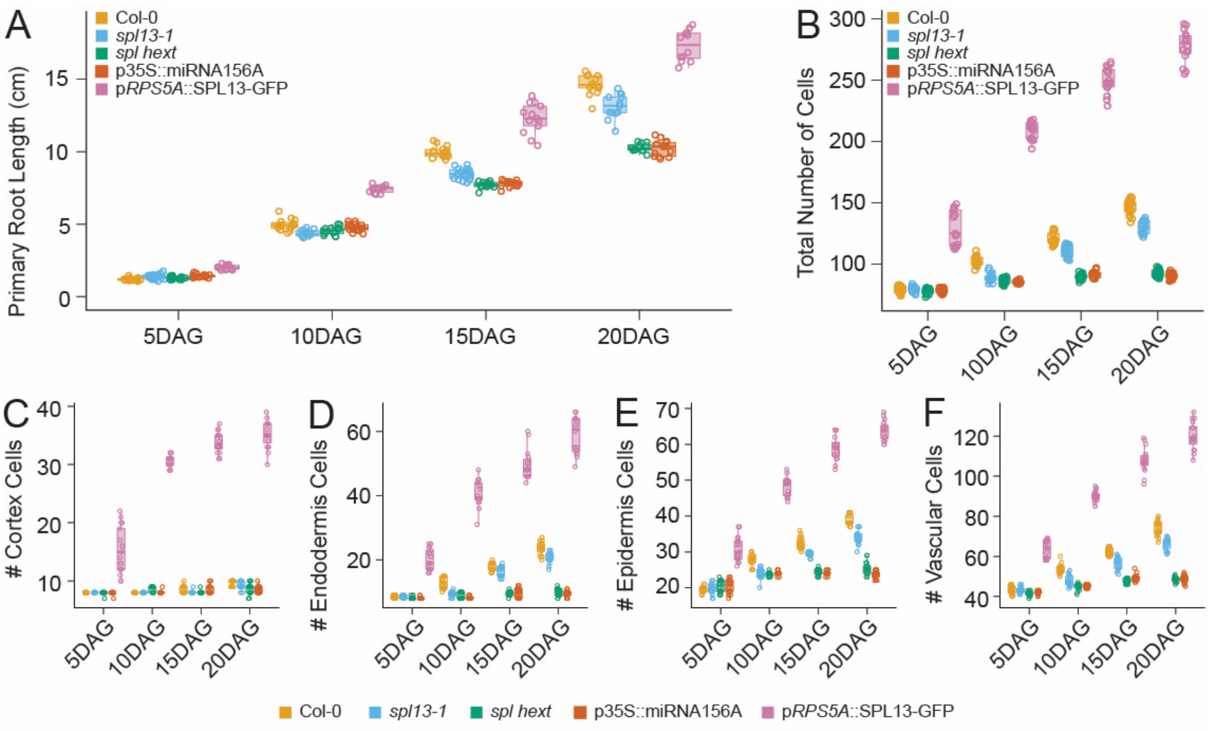

Fig. S15. Changes in root meristem characteristics over 20 days of growth
(**A**) Quantification of the primary root length of the indicated genotypes for the indicated durations. (**B**) Quantification of the total number of cells from cross sections in the indicated genotypes for the indicated durations. (**C-F**) Quantifications of the number of cortex (C), endodermis (D), epidermis (E) and stele (F) cells from cross sections in the indicated genotypes for the indicated durations. Note that (B) is the sum of (C-F). For all panels, number of samples analyzed and statistical significance groups are included in the **Data S5** to maintain readability of the figure.

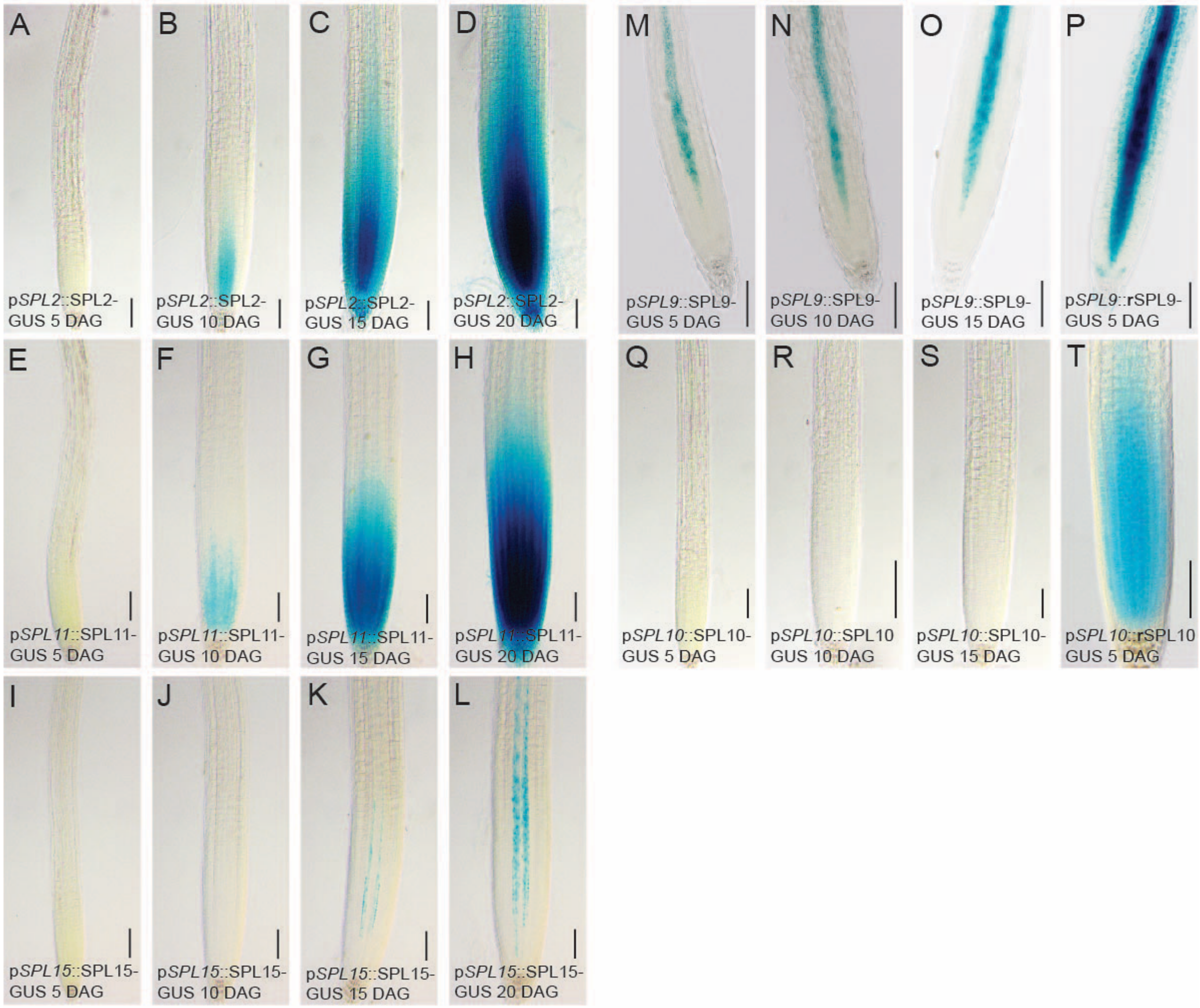

Fig. S16. Localization of SPL proteins in the root meristem over time
(**A**-**T**) GUS staining of the indicated genotypes on the indicated timepoints after germination. Note that multiple SPL proteins increase in abundance over time. (P) and (T) represent the miRNA156 resistant lines activity at 5 DAG versus the sensitive versions in respectively (M) and (Q). Scale bar is 100 µm.

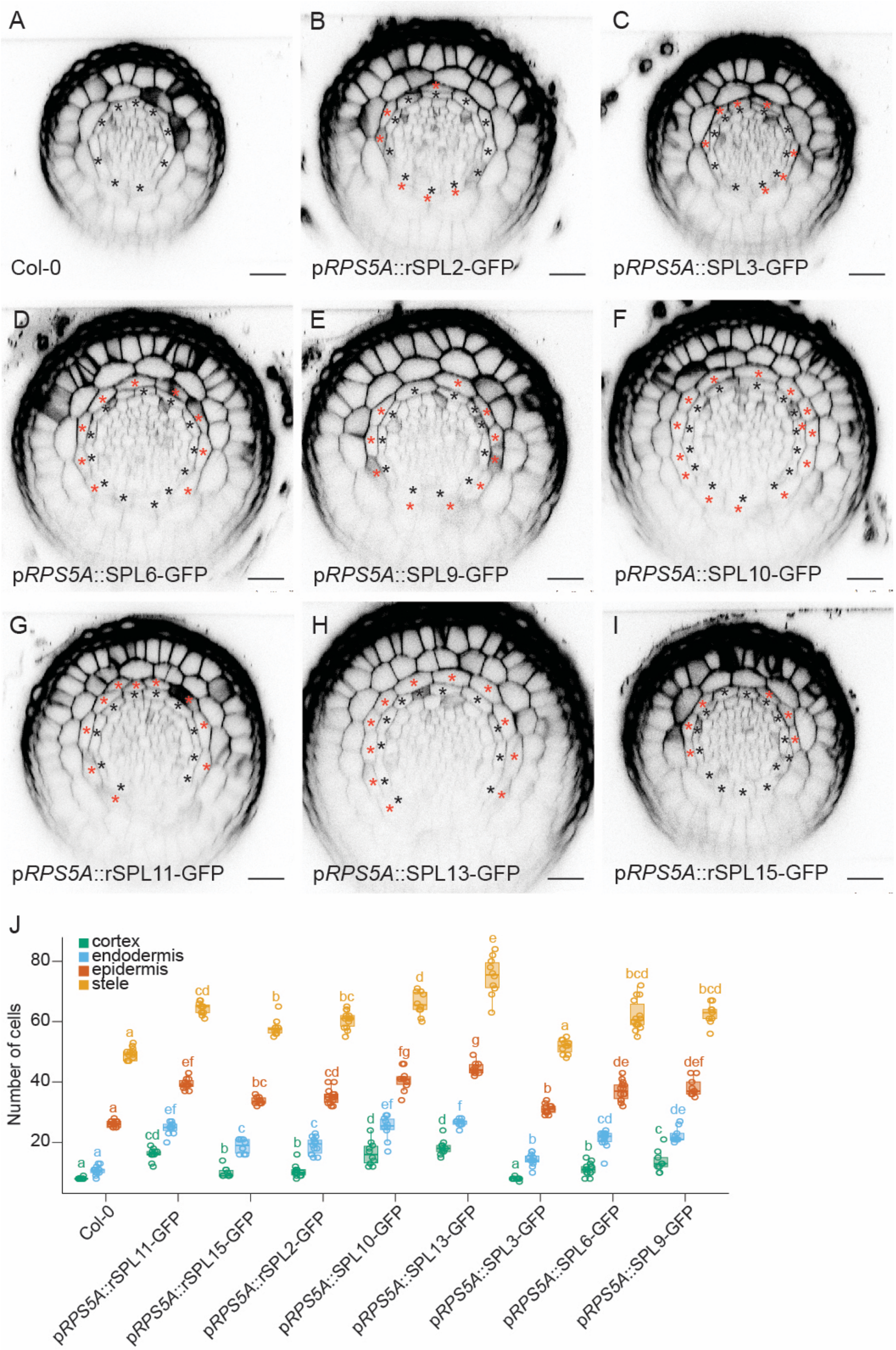

Fig. S17. Misexpression phenotypes of SPL proteins in the root apical meristem
(**A-I**) Optical confocal cross sections of 10 DAG root meristems of the indicated genotypes counterstained using PI. Scale bar is 25 µm. Black asterisks indicate the endodermis cells; red asterisks middle cortex cells. (**J**) Quantification of the number of cells in (A-I) (for n: see **Data S5**).

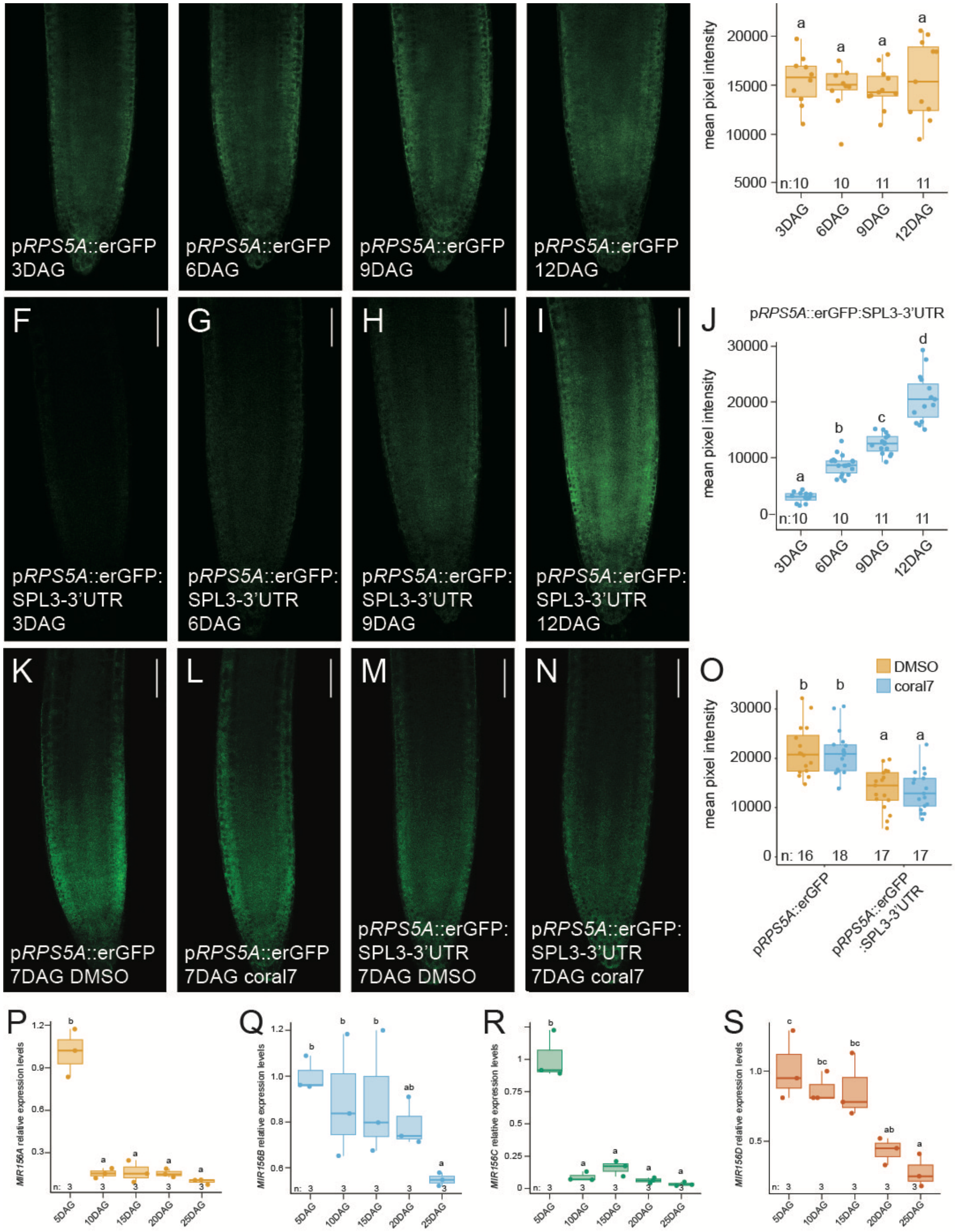

Fig. S18. miR156 levels decrease in root apical meristems over time
(**A-J**) Development of a miR156 sensor construct in Arabidopsis root apical meristems. The control p*RPS5A*::erGFP construct does not change in intensity over time, whereas the p*RPS5A*::erGFP-SPL3-3'UTR construct capable of being degraded by miR156 increases in

expression over time, indicating a reduction in miR156 levels. Scale bar in A-D and F-I is 50 µm. Panels (E) and (J) are quantifications of representative images in (A-D) and (F-I), respectively. (**K**-**O**) Evaluation of coral7 treatment on miR156 sensor in Arabidopsis root meristems. Confocal images of 7 DAG root meristems expressing p*RPS5A*::erGFP control plants and p*RPS5A*::erGFP-SPL3-3'UTR miR156 sensor plants under 24 hours treatment in DMSO control and coral7 (50 µM). Panel (O) is the quantification of representative images in (K-N). Scale bar in K-N is 50µm. (**P**-**S**) Relative expression levels of *MIR156A-D* over time in the root apical meristem as determined by qRT-PCR analysis.

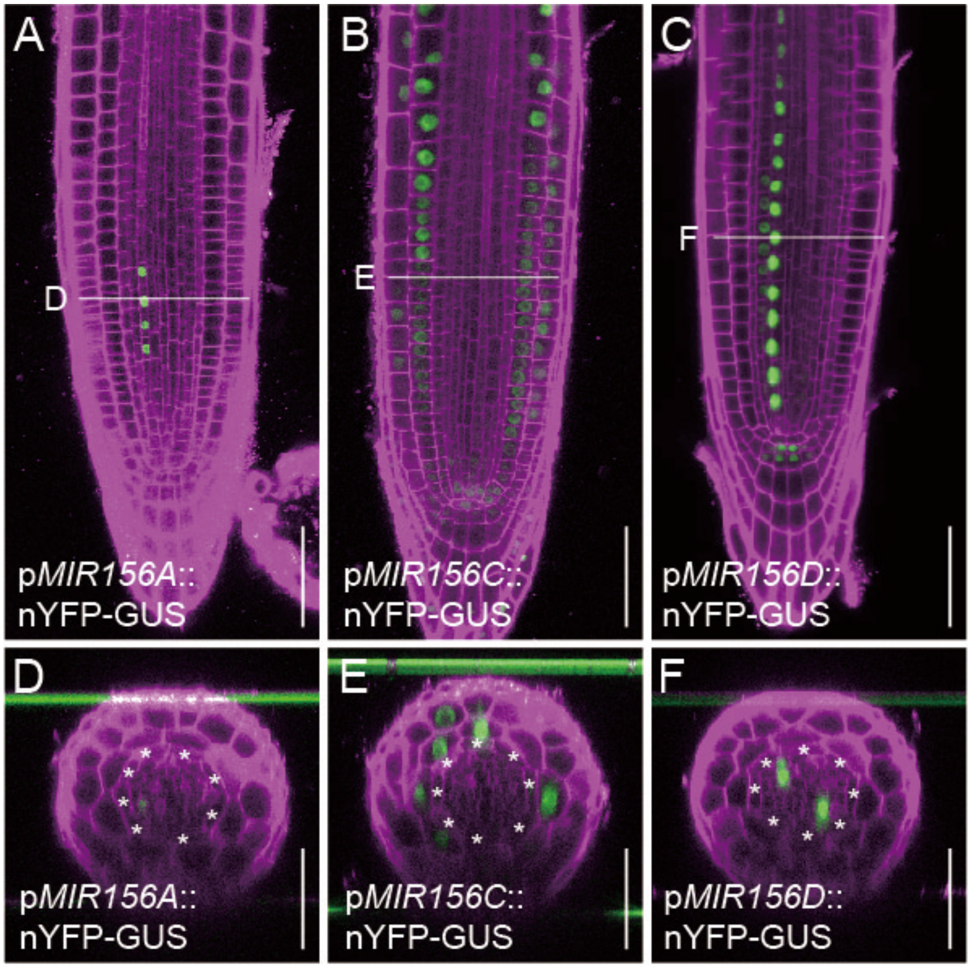

Fig. S19. Expression patterns in *MIR156* isoforms in the root apical meristem
(**A**-**C**) Confocal images of 5 DAG root meristems of the indicated genotypes counterstained using PI (magenta). YFP expression of the *MIR156* genes is shown as green nuclei. Scale bar is 50 µm. (**D**-**F**) Optical confocal cross sections of root meristems of the indicated genotypes counterstained using PI at the position indicated in (A-C). Scale bar is 50 µm.

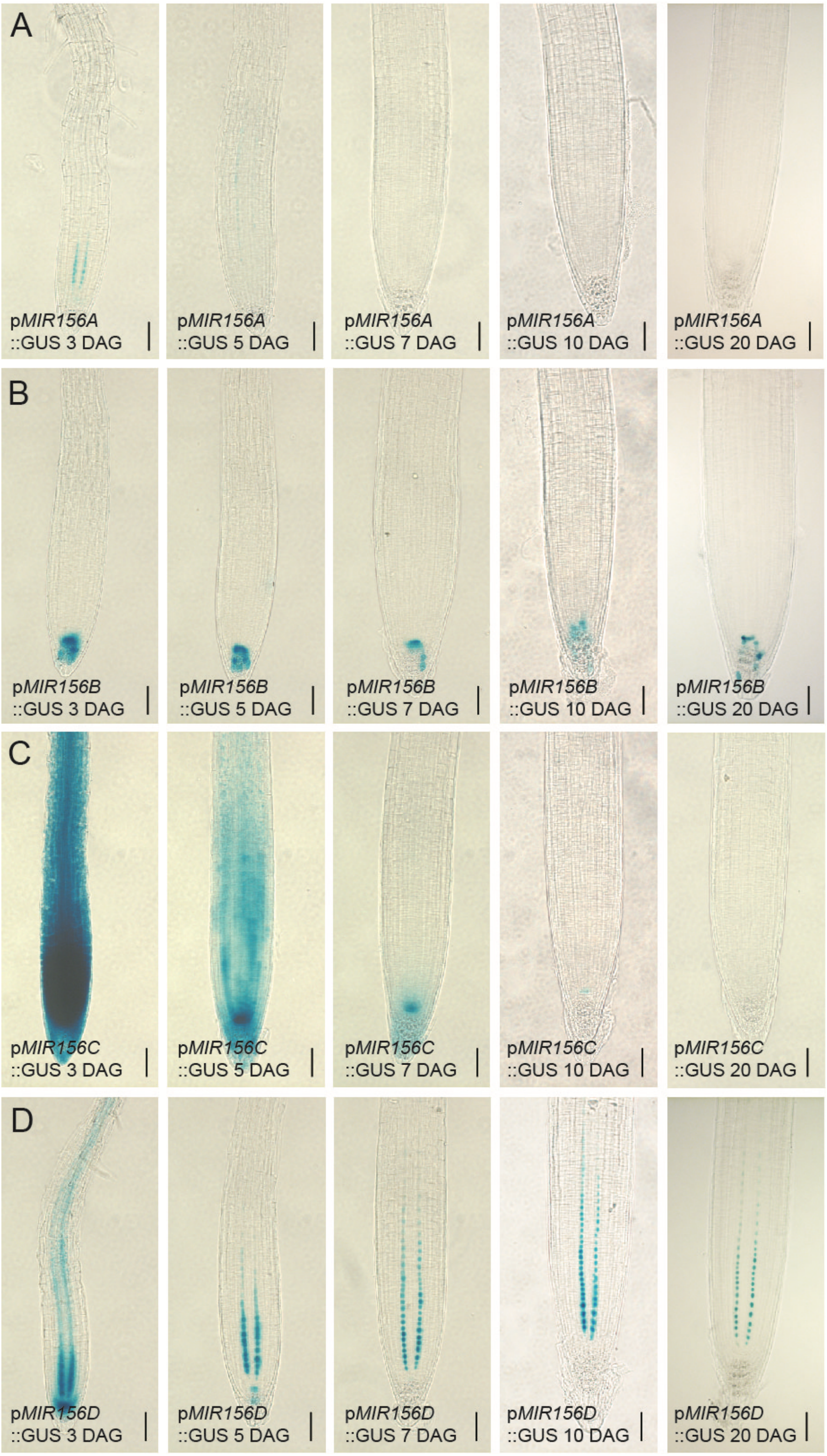

Fig. S20. Expression patterns of *MIR156* genes in the root meristem over time
(**A**-**D**) GUS staining of the indicated genotypes on the indicated timepoints after germination. Note that all *MIR156* isoforms reduce in expression levels over time. Scale bar is 50 µm.

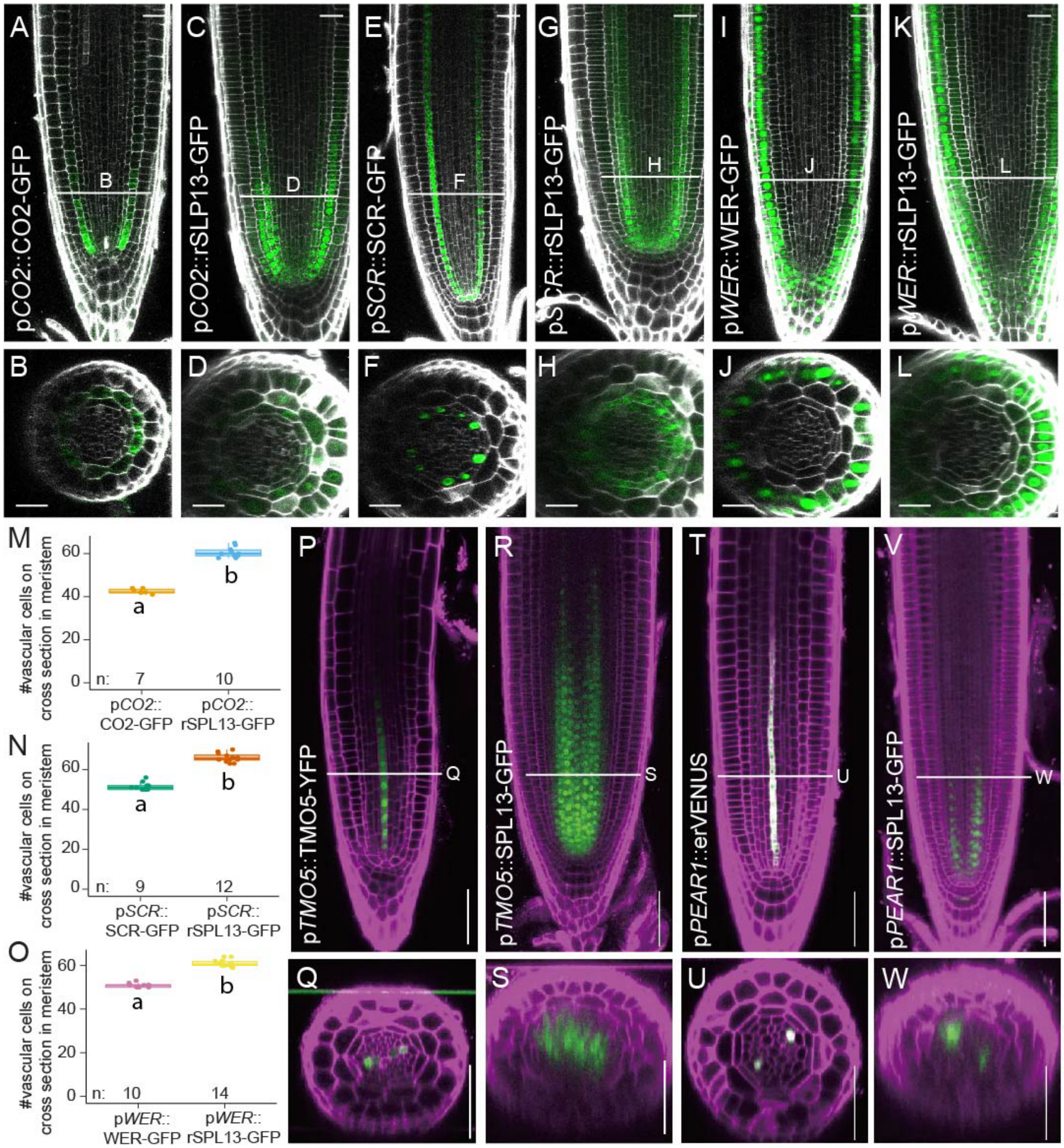

Fig. S21. SPL13 is a mobile protein in the root apical meristem
(**A-L**) Confocal images on longitudinal and cross sections of 10 DAG root meristems of the indicated genotypes counterstained using PI (white). Respective translational protein fusions are shown in green. (**M-O**) Quantifications of the number of vascular cells on cross sections from (A-L). (**P-W**) Confocal images on longitudinal and cross sections of 7 DAG root meristems of the indicated genotypes counterstained using PI (magenta). Scale bars are 25 µm in (A-L), 50 µm in (P-W). Note that the fusion proteins move outside the expression domains and trigger cell divisions in the vascular (M-O).

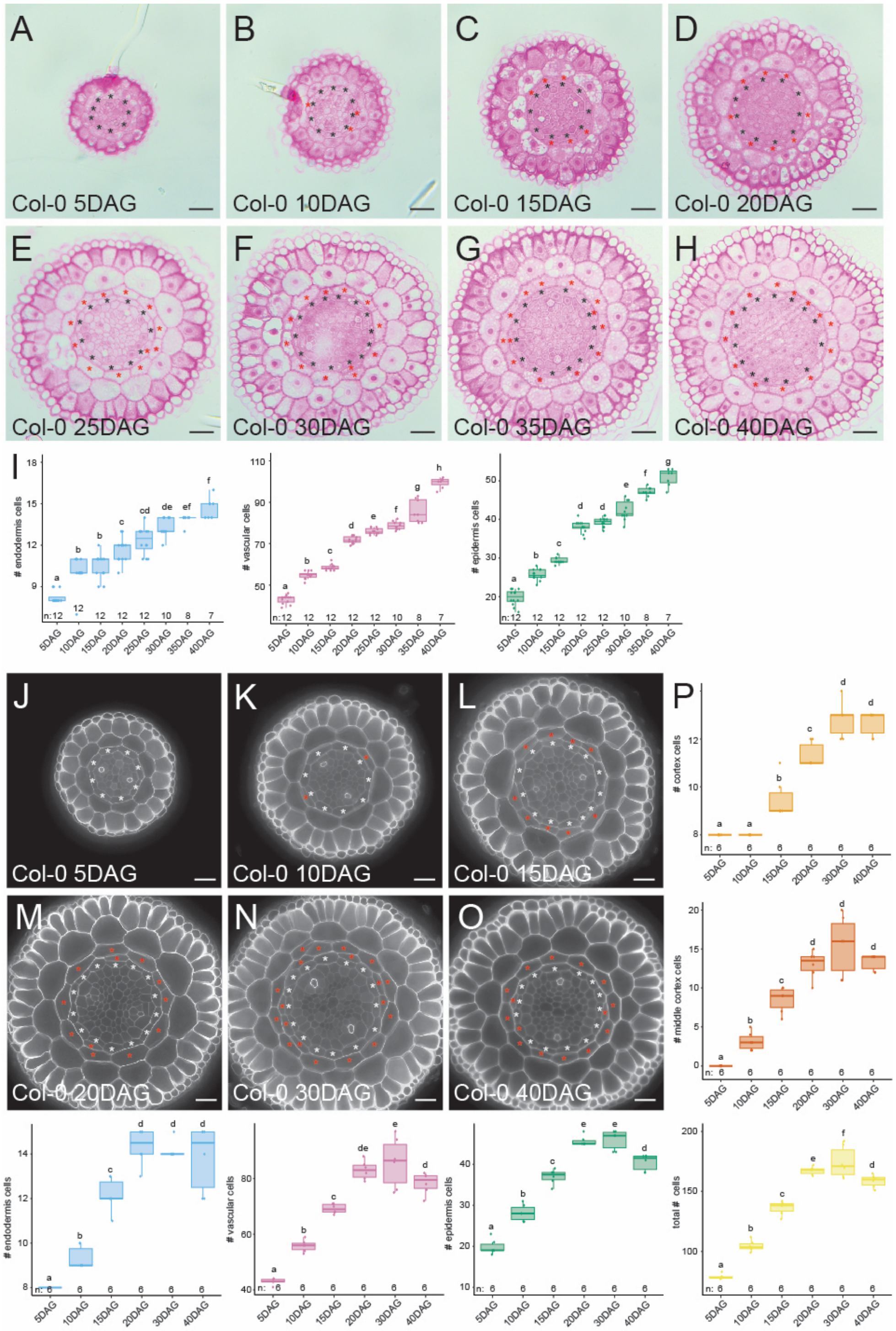

Fig. S22: Morphological changes in the root apical meristem over time
(**A-H**) Histochemical cross sections through the root meristem of seedlings grown on control medium in *in vitro* conditions from 5 to 40 DAG for indicated genotypes. Scale bar is 25 µm. (**I**) Quantification of the cell numbers in (A-H) (see also Fig. 3M-T). (**J-O**) Confocal optical cross sections of root meristems of Col-0 plants grown in soil for the indicated time and counterstained with calcofluor white (white). (**P**) Quantification of the cell numbers in J-O. Scale bar is 20 µm. Black asterisks indicate the endodermis cells; red asterisks middle cortex cells.

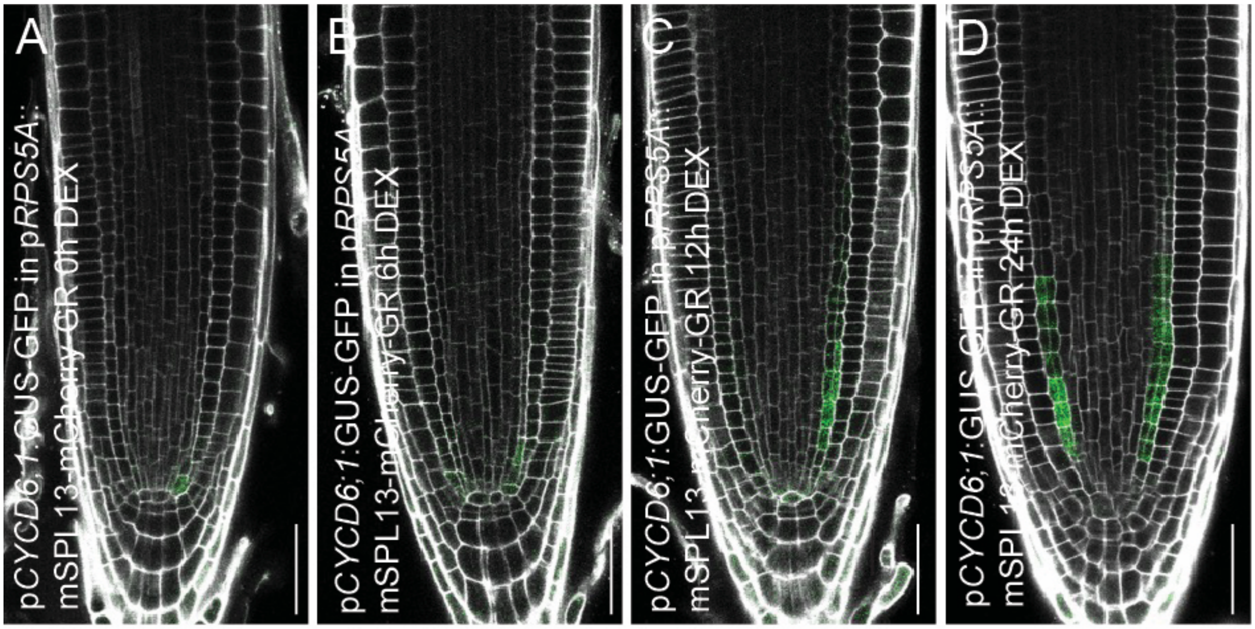

Fig. S23: Pathways acting downstream of SPL13
(**A**-**D**) Confocal images of 6 DAG root meristems expressing p*CYCD6;1*::GUS-GFP in a p*RPS5A*::mSPL13-mCherry-GR background, induced with 10 µM DEX for the indicated time and counterstained with propidium iodide (white). Scale bars are 50 µm.

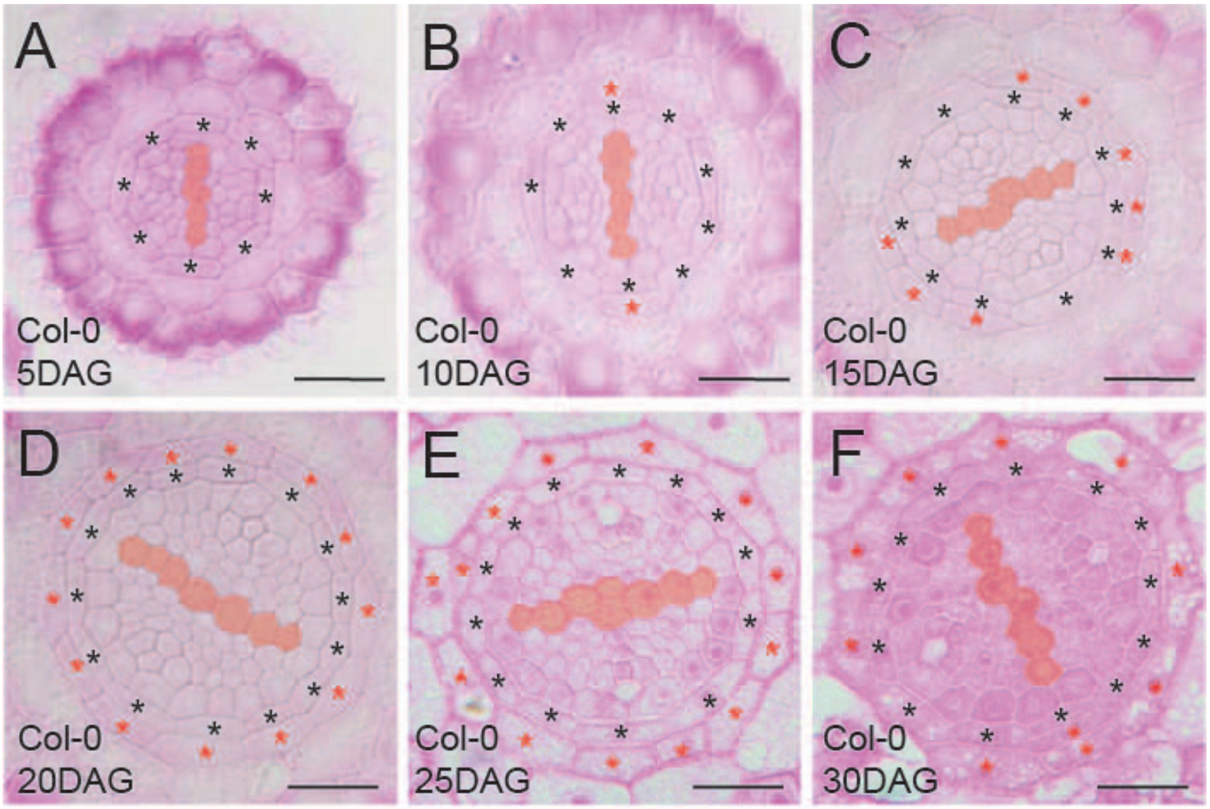

Fig. S24. Middle cortex formation in the root apical meristem over time (A-F).
Note the first pair of divisions occur at the endodermis cells at the xylem poles at 10 DAG and then extend around the entire root circumference at 20 DAG. At later stages, such as at 25 and 30 DAG, a second middle cortex cell layer will be generated. Black asterisks indicate the endodermis cells while red asterisks indicate cells contributing to the middle cortex layer. Orange highlighted cells are the xylem cells.

Data S1. (separate file) A summary of BY2 cell phenotype with compounds treatment

Data S2. (separate file) Compound synthesis overview

Data S3. (separate file) Differentially expressed genes in the coral7 treatment dataset

Data S4. (separate file) Primers and constructs used in this study

Data S5. (separate file) Data and statistical analyses
